# Quality-of-Life Outcomes and Toxicity Profile Among Patients With Localized Prostate Cancer After Radical Prostatectomy Treated With Stereotactic Body Radiation: The SCIMITAR Multicenter Phase 2 Trial

**DOI:** 10.1016/j.ijrobp.2022.08.041

**Published:** 2022-08-23

**Authors:** Ting Martin Ma, Leslie K. Ballas, Holly Wilhalme, Ankush Sachdeva, Natalie Chong, Sahil Sharma, Tiffany Yang, Vincent Basehart, Robert E. Reiter, Christopher Saigal, Karim Chamie, Mark S. Litwin, Matthew B. Rettig, Nicholas G. Nickols, Stephanie M. Yoon, Lauren Smith, Yu Gao, Michael L. Steinberg, Minsong Cao, Amar U. Kishan

**Affiliations:** * Department of Radiation Oncology, University of California, Los Angeles, California; † Department of Radiation Oncology, Cedars-Sinai Medical Center, Los Angeles, California; ‡ Department of Medicine Statistics Core, Division of General Internal Medicine and Health Services Research; § Department of Urology; ║ Department of Health Policy and Management, Fielding School of Public Health; ¶ School of Nursing; # Department of Medicine, University of California Los Angeles, Los Angeles, California

## Abstract

**Purpose:**

Postoperative radiation therapy (RT) is an underused standard-of-care intervention for patients with prostate cancer and recurrence/adverse pathologic features after radical prostatectomy. Although stereotactic body RT (SBRT) is a well-studied and convenient option for definitive treatment, data on the postprostatectomy setting are extremely limited. The purpose of this study was to evaluate short-term physician-scored genitourinary (GU) and gastrointestinal (GI) toxicities and patient-reported outcomes after postprostatectomy SBRT.

**Methods and Materials:**

The SCIMITAR trial was a phase 2, dual-center, open-label, single-arm trial that enrolled patients with postoperative prostate-specific antigen >0.03 ng/mL or adverse pathologic features. Coprimary endpoints were 4-year biochemical recurrence–free survival, physician-scored acute and late GU and GI toxicities by the Common Terminology Criteria for Adverse Events (version 4.03) scale, and patient-reported quality-of-life (QOL) outcomes, as represented by the Expanded Prostate Cancer Index-26 and the International Prostate Symptom Score. Patients received SBRT 30 to 34 Gy/5 fractions to the prostate bed ± bed boost ± pelvic nodes with computed tomography (CTgRT) or magnetic resonance imaging guidance (MRgRT) in a nonrandomized fashion. Physician-scored toxicities and patient-reported QOL outcomes were collected at baseline and at 1, 3, and 6 months of follow-up. Univariable and multivariable analyses were performed to evaluate predictors of toxicities and QOL outcomes.

**Results:**

One hundred participants were enrolled (CTgRT, n = 69; MRgRT, n = 31). The median follow-up was 29.5 months (CTgRT: 33.3 months, MRgRT: 22.6 months). The median (range) prostate bed dose was 32 (30-34) Gy. Acute and late grade 2 GU toxicities were both 9% while acute and late grade 2 GI toxicities were 5% and 0%, respectively. Three patients had grade 3 toxicity (n = 1 GU, n = 2 GI). No patient receiving MRgRT had grade 3 GU or grade ≥2 GI toxicity. Compared with CTgRT, MRgRT was associated with a 30.5% (95% confidence interval, 11.6%-49.5%) reduction in any-grade acute GI toxicity (*P* = .006). MRgRT was independently associated with improved any-grade GI toxicity and improved bowel QOL.

**Conclusions:**

Postprostatectomy SBRT was well tolerated at short-term follow-up. MRgRT may decrease GI toxicity. Longer toxicity and/or efficacy follow-up and randomized studies are needed.

## Introduction

For patients with biochemical recurrence after radical prostatectomy and those at high risk of biochemical recurrence, postoperative radiation therapy (RT) directed to the prostate bed and/or pelvic lymph nodes is recommended, with early salvage RT generally preferred.^1–9^ However, all forms of postoperative RT remain underused.^10,11^ This may in part be due to the protracted course of therapy, as conventional fractionation (1.8 Gy/fraction over 30-33 daily fractions) remains standard. This places both logistical and financial burdens on patients.^12^

For definitive RT, moderately hypofractionated RT (>2.4 Gy/fraction) and ultrahypofractionated RT (>5 Gy/fraction) are considered standard of care.^5,13–17^ Stereotactic body RT (SBRT) is a form of ultrahypofractionated RT that uses sophisticated radiation planning and delivery technologies to deliver ≤5 treatments. Potential SBRT benefits include leveraging radiobiology (with prostate cancer cells thought to experience greater death with high doses per fraction^18,19^), increased patient convenience, greater access to care, and lower health care costs.^20–23^ Whereas moderately hypofractionated postprostatectomy RT has shown favorable results in several phase 2 studies and 1 phase 3 study,^24–35^ postprostatectomy SBRT has only been evaluated in 2 small single-institution phase 1 studies.^36,37^

Despite an acceptable toxicity profile, concerns regarding the highly deformable and mobile prostate bed clinical target volume (which is adjacent to the bladder, rectum, and vesicourethral anastomosis), have precluded further study in the phase 2 setting. However, now a standard option in definitive treatment of intact prostate cancer, further exploration of postprostatectomy SBRT is warranted, particularly given the aforementioned advantages and the context of widespread underutilization. Patient-reported quality of life (QOL) may be affected by SBRT and needs to be preserved before SBRT can be adopted in the postprostatectomy setting. Moreover, the emerging technology of magnetic resonance imaging (MRI)–guided RT (MRgRT) offers several theoretical advantages over standard computed tomography (CT)–guided RT (CTgRT), including enhanced prostate bed visualization, precise visualization of the boundary between the prostate bed and surrounding organs at risk (OARs), the ability to track organ motion in real time, and the capacity to perform online adaptive planning.^38^

This multicenter phase 2 study was designed to evaluate postprostatectomy SBRT with prostate bed doses of 30 to 34 Gy in 5 fractions in terms of oncologic efficacy, physician-scored toxicity, and patient-reported outcomes. We also prespecified an exploratory comparison of toxicity profiles for patients treated with CTgRT versus MRgRT.

## Methods and Materials

### Study design

The Stereotactic Intensity Modulated Radiotherapy After Radical Prostatectomy (SCIMITAR) trial was a dual-center (University of California, Los Angeles [UCLA] and University of Southern California [USC], Los Angeles, California, USA) phase 2 single-arm trial activated in February 2018 (NCT03541850) that evaluated the toxicity, QOL, and treatment efficacy of postoperative SBRT for prostate cancer. The study was approved by the institutional research boards of the participating centers. All participants were provided written informed consent forms before trial enrollment in a manner that was consistent with the Declaration of Helsinki.^39^ Participation was entirely voluntary with no compensation or incentive offered.

### Study endpoints

The primary endpoint of the study was the efficacy of SBRT in the postoperative setting, defined as 4-year biochemical recurrence–free survival (BCRFS). A coprimary endpoint was physician-scored toxicity, represented by the rates of acute (<90 days post-SBRT) and late (≥90 days after SBRT) genitourinary (GU) and gastrointestinal (GI) toxicity per the Common Terminology Criteria for Adverse Events (CTCAE), version 4.03. Physicians were not blinded to the radiation platform assignment (ie, CTgRT vs MRgRT). A second coprimary endpoint was patient-reported outcome profiles, as represented by changes in the urinary incontinence, urinary irritative/obstruction, bowel, and sexual function domains of the Expanded Prostate Cancer Index-26 (EPIC-26)^40^ QOL instrument and longitudinal changes in the International Prostate Symptom Score (IPSS).^41^ A comparison of the toxicity and QOL profiles between patients treated with CTgRT versus MRgRT was a prespecified exploratory analysis. This publication focuses on the toxicity and patient-reported QOL-related primary endpoints and exploratory endpoint.

### Eligibility

Eligible patients must have had a history of clinical localized adenocarcinoma of the prostate treated with radical prostatectomy. Additionally, patients must have had at least one of the following: (1) adverse pathologic features at the time of prostatectomy (positive surgical margin, pathologic T3/T4 disease, pathologic Gleason score 8-10 disease or presence of tertiary Gleason grade 5 disease), (2) rising prostate-specific antigen (PSA) on at least 2 consecutive measurements, with both >0.03 ng/mL, or (3) a Decipher genomic classifier score^42^ >0.45. A CT scan, MRI of the pelvis and a bone scan within 120 days before enrollment were required. Positron emission tomography was strongly encouraged.

### Postoperative SBRT

Before the commissioning of an MRI linear accelerator at UCLA in March 2020, all patients were treated with CTgRT using a NovalisTx or TrueBeam (Varian, Inc, Palo Alto, CA). Subsequent to that, all patients treated at UCLA were offered treatment on the 0.35T MRI-guided MRIdian (ViewRay, Inc, Mountain View, CA); of these, 4 of 37 (11%) declined due to claustrophobia (n = 2) or pacemaker (n = 2). All USC patients were treated with CTgRT. Pelvic nodal RT and 6 months of androgen-deprivation therapy (ADT) were used at the discretion of the treating physician. Specific bladder and rectum preparation protocols, described previously, were in place for simulation and for each fraction.^43,44^ A planning CT was performed with patients immobilized with a custom vacuum lock bag. Patients treated with MRgRT underwent an additional 0.35T MRI simulation as previously described.^45^ Clinical target volumes (CTV) of the prostate bed (CTV_PB_) and pelvic lymph node volume (CTV_N_, when treated) were delineated in accordance with the Radiation Therapy Oncology Group (RTOG) consensus guideline.^46^ If gross tumor in the prostate bed or the pelvic lymph nodes was visible on preradiation imaging, it was contoured as gross tumor volume (GTV_boost_).

CTV_PB_ was expanded isotopically by 5 mm (CTgRT) or 3 mm (MRgRT) to form the corresponding planning target volumes (PTV_PB_); expansions for CTV_N_ and GTV_boost_ were generally 5 and 3 mm, respectively, regardless of platform. Plans were designed to deliver 30 to 34 Gy, 25 Gy, and 35 to 40 Gy in 5 fractions to PTV_PB_, PTV_N_, and PTV_boost_, respectively, such that 95% of each PTV received prescription dose, unless doing so would lead to violations of OAR constraints (Appendix E1 Trial Protocol). Sample CTgRT and MRgRT plans are shown in Figs. E1 and E2. RT was delivered every other day. For CTgRT volumetric modulated arc therapy was used with a kilovoltage cone beam CT acquired before each treatment to verify anatomy. For MRgRT, 13 to 17 static gantry intensity modulated RT fields were used, and a set-up MRI was acquired before each treatment. During MRgRT delivery, sagittal cine MRI images were acquired at 4 frames per second to track the anterior rectal wall motion in real time. A gating boundary of 3 mm around the anterior rectal wall was set such that if >10% of the volume moved outside this window, beam delivery was automatically paused. Online adaptive RT was delivered in a minority of MRgRT fractions (4 fractions or 2.5%) owing to staffing restrictions during the COVID-19 pandemic in the setting of unclear clinical benefit.

### Assessments

Physician-scored toxicity and patient-reported outcomes were collected at baseline, at 1 and 3 months after treatment completion, at every 3 months for the first year after treatment, and then every 6 months for a minimum of 4 years after treatment. PSA was drawn every 3 months for the first year, then every 6 months until 4 years after SBRT. Beyond 4 years, patients were assessed on a yearly basis.

### Statistical analysis

Descriptive analysis was conducted for all patients, with medians, interquartile ranges, and ranges calculated for continuous variables and proportions calculated for categorical variables. Patient responses on the EPIC-26 questionnaires were scored as per scoring manual and were summarized and graphed by mean and 95% confidence interval (CI) of change in scores at each follow-up visit compared with the baseline. Minimally clinically important difference values of 6, 5, 4, and 10 points for the urinary incontinence domain, urinary irritative/obstructive domain, bowel domain, and sexual domain, respectively, were used in accordance with prior literature.^47^ χ^2^ tests were used to assess the association of SBRT platform and frequency of toxicity by grade. The Mann-Whitney test was used to compare baseline toxicity grade frequencies between the SBRT platforms. Wilcoxon matched-pairs signed rank test was used to compare EPIC-26 subdomain and IPSS scores at various time points to the baseline.

Univariable analyses were performed to evaluate associations between the selected variables (Tables E10–17) and physician-scored GU, GI, and/or sexual toxicity within 6 months of SBRT. Logistic regression was used to develop multivariable prediction models for any-grade GI and GU toxicity and clinically relevant (1 × minimally clinically important difference) change in EPIC-26 bowel domain within the first 6 months. Covariates were selected by a combination of clinical relevance and/or significance on univariable analyses. The analyses reported were specified a priori. All analyses were performed using SAS version 9.4 (SAS Institute, Cary, NC). Statistical significance was set at a 2-sided *P* value of <.05.

## Results

### Patients

**Fig. 1.**
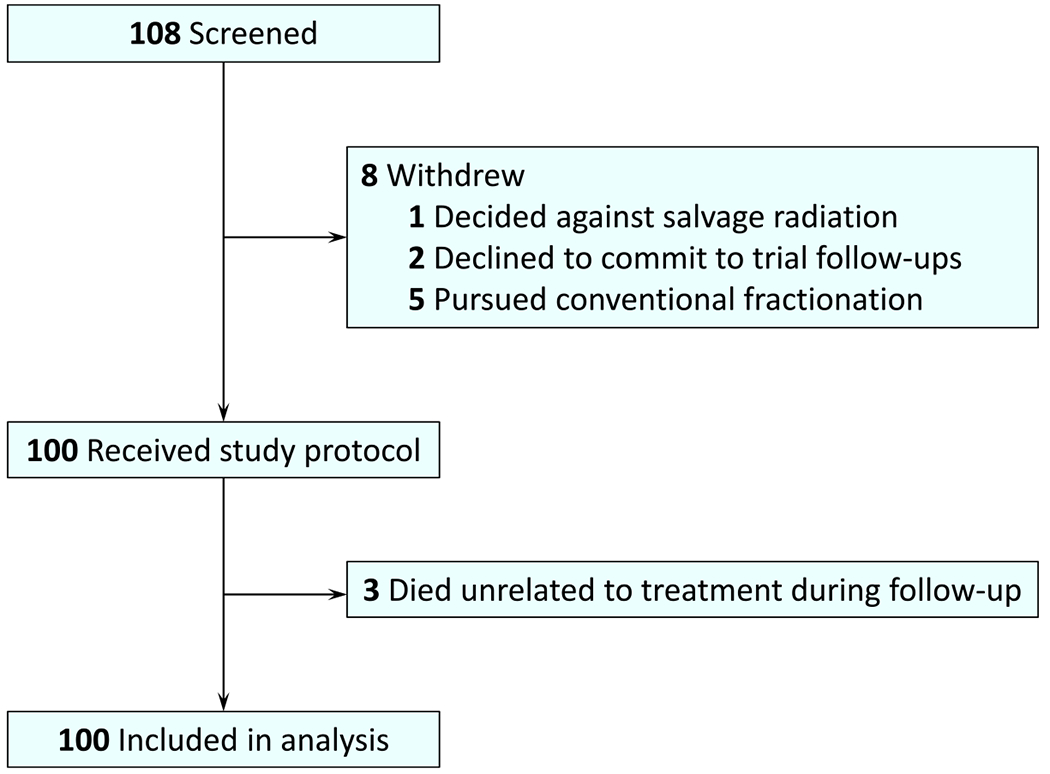
Flow diagram of patient accrual and analysis.

From February 2018 to March 2021, 108 patients were screened and enrolled in the SCIMITAR trial at UCLA (100 patients) and USC (8 patients). Eight patients withdrew from the study before starting treatment, thus leaving 100 patients (median age, 69 years; range, 50-82) who completed the study treatment (Fig. 1). Baseline demographic and clinical characteristics as well as treatment parameters are shown in Table 1. Sixty-nine (69%) patients underwent CTgRT while 31 (31%) received MRgRT. The median pre-SBRT PSA was 0.3 ng/mL (range, 0.0-9.3 ng/mL) and 76% of patients received advanced imaging. The median prostate bed dose was 32 Gy (range, 30-34 Gy). Twenty-seven (27%) patients received a prostate bed boost, 27 (27%) received elective pelvic nodal RT, and 5 (5%) received a boost to gross pelvic nodes. The median follow-up was 29.5 months (33.3 months for CTgRT and 22.6 months for MRgRT). Three patients died during the follow-up period, all unrelated to prostate cancer treatment (n = 1 each from congestive heart failure, myocardial infarction, and intracranial hemorrhage).

### Physician-scored CTCAE toxic effects

**Fig. 2.**
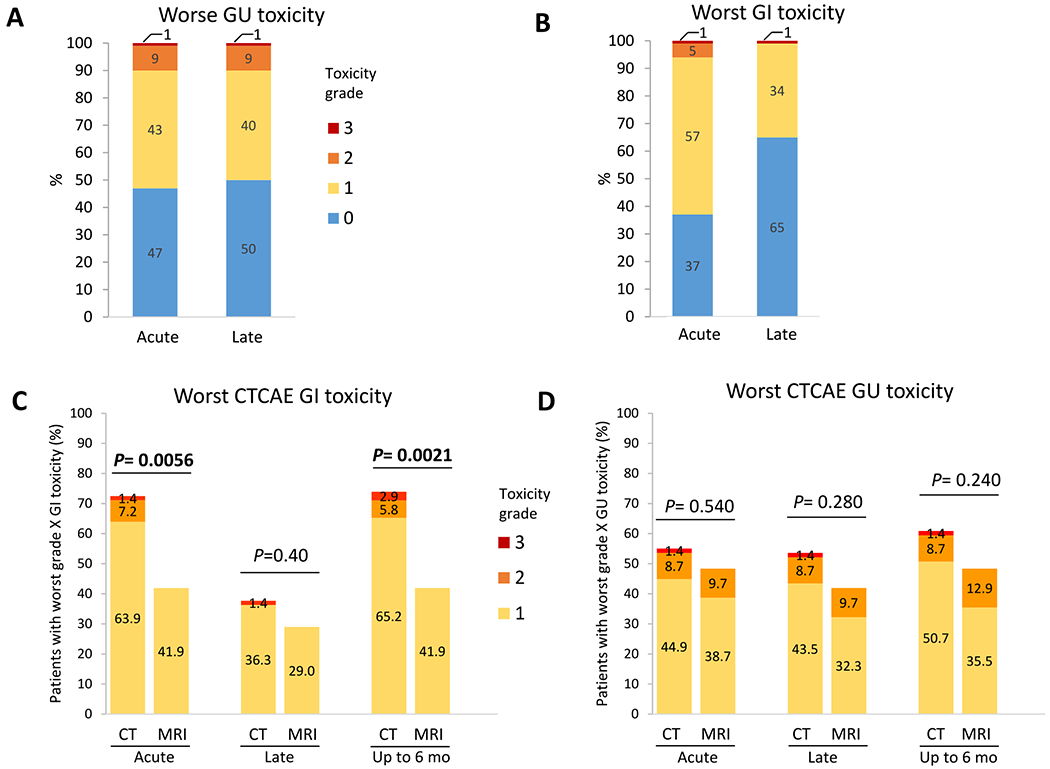
(A-D) Physician-scored acute and late genitourinary (GU) and gastrointestinal (GI) toxicities and breakdown by radiation delivery platforms. Physician-score toxicities were graded according to the Common Terminology Criteria for Adverse Events (CTCAE) version 4.03. *P* values in (C) and (D) apply to between-platform comparisons (computed tomography– [CT] vs magnetic resonance imaging (MRI)–guided stereotactic body radiation therapy [SBRT]) of any-grade toxicities at the specified period. *P* values were calculated with the χ^2^ test, and those <.05 are bolded. Acute toxicities are side effects possibly, probably, or definitely related to radiation treatment within 90 days of SBRT, whereas late toxicities are those occurring 90 to 180 days after SBRT.

Physician-scored acute and late GU and GI toxicities are shown in Fig. 2A–B. Detailed breakdown by time points and symptoms is shown in Tables E1 and E2. The majority of worst acute toxicity events were grade 1 for GU and GI toxicity, at 43% and 57%, respectively. Late grade 1 GU and GI toxicity rates were 40% and 34%, respectively. Worst acute grade 2 GU and GI toxicity rates were 9% and 5%, respectively. The incidences of late grade 2 GU and GI toxicities were 9% and 0%, respectively. No grade ≥2 GI toxicity events were seen in patients treated with MRgRT. Grade 3 toxicity occurred in 3 patients, with one experiencing grade 3 acute and late GU toxicity and one each with grade 3 acute and late GI toxicity, all treated with CTgRT.

One patient (1%) developed grade 3 hematuria due to radiation cystitis 1 month after SBRT, which persisted with medical management and was ultimately successfully treated with hyperbaric oxygen therapy. One patient (1%) had severe grade 3 acute GI toxicity (diarrhea) 1 week after the completion of SBRT, which resolved with conservative medical management. A third patient, who was on immunosuppressive medications, developed grade 2 radiation proctitis 6 months after SBRT. A colonoscopy was performed at the 9-month mark, which revealed ulcerations that were aggressively biopsied by a specialist not affiliated with either trial institution. This showed histoplasmosis, rather than radiation effect, and the patient received a diagnosis of disseminated histoplasmosis, which he was estimated to have acquired due to occupational exposure. After extensive multidisciplinary review, this was scored as a grade 3 GI toxicity with respect to SBRT in the interest of conservatively estimating toxicity.

A comparison of the CTgRT and MRgRT with respect to physician-scored toxicity is shown in Fig. 2C–D, Tables E3 to E8, and Fig. E3. Compared with CTgRT, MRgRT was associated with significantly lower rates of any-grade acute GI toxicity (41.9% vs 72.5%, *P* = .006, or an estimated absolute reduction of 30.5% [95% CI, 11.6%-49.5%]) and significantly lower rates of any-grade GI toxicity of up to 6 months after SBRT (41.9% vs 73.9%, *P* = .002, or an estimated absolute reduction of 32% [95% CI, 12.9%-51.1%]). Late any-grade GI toxicity was 37.7% for CTgRT and 29.0% for MRgRT (*P* = .4). Though MRgRT was associated with numerically lower rates of acute (48.4% vs 55.0%, *P* = .54), late (42.0% vs 53.6%, *P* = .28) and up to 6 months (48.4% vs 60.8%, *P* = .24) any-grade GU toxicity, none of these differences were statistically significant.

### Patient-reported outcomes and QOL

**Fig. 3.**
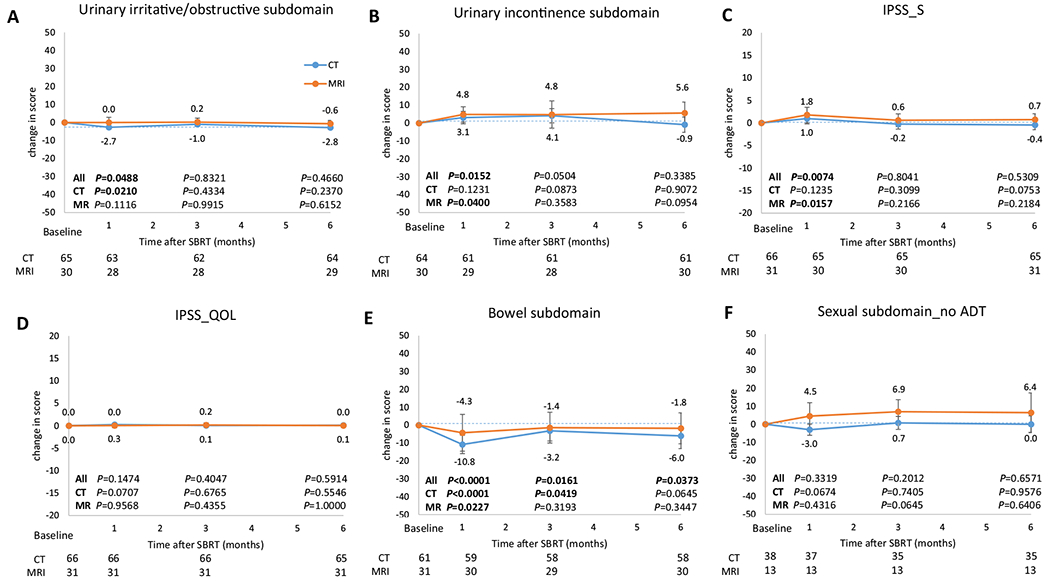
Changes from baseline in Expanded Prostate Cancer Index composite (EPIC-26) subdomains and International Prostate Symptom Score (IPSS). (A, B, E, F) Derived from the EPIC-26 questionnaire. (C, D) Derived from the IPSS questionnaire. Data represent subdomain median scores, with error bars showing 95% confidence intervals. Scores are change from baseline, with 0 representing no change. IPSS_S is the sum score of the first 7 questions (incomplete emptying, frequency, intermittency, urgency, weak stream, and nocturia) in the IPSS questionnaire. Higher numbers denote worse urinary symptoms. IPSS_QOL is the score of the quality-of-life question related to urinary symptoms in the IPSS questionnaire. A score of 0 is “delighted” and 6 is “terrible.” *P* values comparing the scores at baseline and at corresponding time points (1, 3, and 6 months after stereotactic body radiation therapy [SBRT]) in the overall cohort and computed tomography (CT)–guided (CTgRT) and magnetic resonance imaging (MRI)–guided SBRT (MRgRT) groups are shown inside the graphs. Bolded *P* values denote statistically significant differences compared with baseline. *P* values were calculated using the Wilcoxon matched-pairs signed rank test. The number of patients who completed the questionnaire at each time point are recorded below each graph. *Abbreviation:* ADT = androgen deprivation therapy.

Figure 3 and Table E9 show changes in EPIC-26 and IPSS scores over time while Fig. E4 shows the proportion of patients with a clinically relevant deterioration in EPIC-26 domains over time. There was a consistent decline in EPIC-26 urinary irritative/obstructive domain score (mean, −2.9; 95% CI, −4.9 to −0.9; *P* = .049) and increase in IPSS sum scores (IPSS_S) (mean, 1.2; 95% CI, 0.2-2.2; *P* = .007) 1 month after SBRT; both normalized by 3 months post-SBRT. No statistically significant deterioration in the urinary irritative/obstructive domain was seen at any time in patients treated with MRgRT, and no significant changes in urinary incontinence or the IPSS QOL summary scale were seen with either platform. A statistically significant decline in the EPIC-26 bowel domain score was observed at 1 month (mean, −8.6; 95% CI, −12.4 to −4.8; *P* < .0001), 3 months (mean, −2.6; 95% CI, −4.8 to −0.4; *P* = .02) and 6 months (mean, −4.6; 95% CI, −8.4 to −0.8; *P* = .04). However, the proportion of patients with clinically relevant deterioration improved from 1 month to 6 months, with the clinically relevant deterioration resolving earlier in men receiving MRgRT. EPIC-26 sexual domain scores were unchanged over time in patients without ADT.

### Predictors of physician-scored toxicities and patient-reported outcomes

We performed univariable (Tables E10–E17) and multivariate analyses (Table 2) to evaluate predictors of toxicity and QOL outcomes. In the univariable analysis, baseline IPSS score (*P* = .010), elective nodal RT (*P* < .001) and baseline urinary pads use (*P* = .02) were significantly associated with any-grade GU toxicities within 6 months of SBRT. In the multivariable analysis, elective nodal RT (odds ratio [OR], 10.30; 95% CI, 2.56-41.43; *P* = .001) and baseline urinary pads use (OR, 2.78; 95% CI, 1.02-7.61; *P* = .046) continued to be significant predictors. CTgRT (*P* = .002), elective nodal RT (*P* = .03), prostate bed boost (*P* = .04), and baseline EPIC-26 bowel domain score (*P* = .02) were significantly associated with any-grade GI toxicity within the first 6 months in the univariable analysis while CTgRT was the only significant predictor (OR, 3.71; 95% CI, 1.38-9.99; *P* = .010) in the multivariable analysis. Similarly, CTgRT (OR, 3.02; 95% CI, 1.12-8.17; *P* = .03) and baseline EPIC-26 bowel score (OR, 1.08; 95% CI, 1.00-1.16; *P* = .047) were significant predictors of clinically relevant decline in EPIC-26 bowel scores within 6 months of SBRT.

## Discussion

The present study, which is the largest prospective study of postprostatectomy SBRT, demonstrates low rates of GU and GI toxicity through 6 months of follow-up and suggests the benefits of MRgRT although the comparison is nonrandomized. Urinary QOL metrics declined at 1 month but were indistinguishable from baseline at 3 months. Whereas a detectable decrease in bowel QOL persisted through 6 months, the proportion of patients with clinically relevant declines improved over time. MRgRT was independently associated with lower any-grade GI toxicity and was less clinically relevant to a decline in bowel QOL on multivariable analysis. Additionally, no grade ≥2 acute GI toxicity or grade 3 acute GU toxicity events were seen in patients treated with MRgRT.

The acute toxicity rates in our study compare favorably to published hypofractionation series,^25,28,29,33^ which report acute grade 2 GU and GI toxicity rates of CTCAE criteria that are approximately 9% to 13% and 9% to 18%, respectively. These are similar to the grade 2 acute GU and GI toxicity rates of 9% and 5% in the present study. The present results also compare favorably with the acute grade ≥2 GU toxicity rates of 0% to 8% and grade ≥2 GI toxicity rates of 33% to 58% reported for patients receiving similar doses on the 2 prior phase 1 SBRT studies,^36,37^ although these studies all used smaller (nonconsensus) clinical treatment volumes without nodal RT or boost doses to gross disease.

The apparent benefits of MRgRT in this setting can likely be attributed to narrower PTV margins (3 mm instead of 5 mm with CTgRT). Notably, standard PTV margins in the context of CTgRT (with moderate hypofractionation) are typically up to 7 mm; the 5 mm margins used for CTgRT on the SCIMITAR protocol are thus also narrow. The further drop to 3 mm with MRgRT was considered safe because of 2 key features of the device: improved visualization of soft tissue with MRI for initial treatment alignment, and realtime treatment gating based on tracking of the anterior rectal wall. The latter may effectively avoid overdosing the rectum when it migrates into the higher dose area due to changes in rectal distention during the course of treatment delivery, and may ultimately also avoid underdosing the clinical target volume.

Notably, the SCIMITAR trial was designed before the publication of the SAKK 09/10 trial, which failed to show a benefit in freedom from biochemical progression with dose-escalation in the salvage setting.^48,49^ Thus, while SCIMITAR was designed to detect a potential improvement in 4-year BCRFS with functional dose-escalation based on ultrahypofractionation, it would no longer be expected to show an oncologic benefit. Nonetheless, we will continue to monitor efficacy and toxicity, with a second planned analysis once the 100th patient has 2-year PRO data available.

## Limitations

This study has several limitations. First, the treatment platform was not randomly allocated and the majority of patients treated with CTgRT were treated earlier in the trial, when planning and treatment experience were lower. Second, the study was limited to 2 tertiary centers, limiting generalizability. Third, physicians were not blinded to the assignment of the RT platform when assessing toxicities. It would have been impossible to blind the patient to the intervention. However, significant differences favoring the MRgRT group were also observed in patient-reported outcomes, which provide a bias-free-as-possible glimpse at true toxicity. Fourth, the rate of online adaptive RT was low, mainly due to the COVID-19 pandemic prioritizing short treatment time and skeleton staffing. The benefit of MRgRT may have been more pronounced had adaptive RT been used.^50^ This concept will be explored in the recently activated phase II EXCALIBUR study (NCT04915508), which will exclusively use MRgRT. Fifth, the follow-up time was short and longer follow-up is needed to truly assess late toxicity. The length of follow-up was also shorter in patients treated with MRgRT compared with those treated with CTgRT. However, all patients in both groups had at least 6 months of follow-up. Finally, as a single arm study, direct comparisons to longer fractionation cannot be made; the randomized phase II SHORTER trial (NCT04422132) is comparing 55 Gy in 20 fractions versus 32.5 Gy in 5 fractions.

## Conclusions

In this multicenter phase 2 trial, postprostatectomy SBRT was well tolerated within the first 6 months as evaluated by both physician-scored toxicity and patient-reported outcomes. MRgRT was associated with significantly decreased GI toxicity and better-preserved bowel QOL compared with CTgRT. Long-term follow-up and randomized trials comparing conventional fractionation or moderate hypofractionation to SBRT are needed to further characterize the safety and efficacy of this technique.

## Supplementary Material

Supplementary Material

## Figures and Tables

**Table 1:** Baseline characteristics and treatment parameters

	Overall cohort (n = 100)	CTgRT (n = 69)	MRgRT (n = 31)	*P* value
Age (y), median (range)	69 (50-82)	69 (52-79)	68 (50-82)	.8573
Race				.4070
Black	9 (9.0%)	5 (7.2%)	4 (12.9%)	
White	85 (85.0%)	60 (87.0%)	25 (80.6%)	
Asian	3 (3.0%)	2 (2.9%)	1 (3.2%)	
Other	2 (2.0%)	2 (2.9%)	0 (0%)	
Unknown	1 (1.0%)	0 (0%)	1 (3.2%)	
PSA at initial diagnosis (ng/mL), median (range)	8.6 (2.7-78.9)	8.0 (2.7-78.3)	9.8 (3.1-78.9)	.2094
Pathologic Gleason GG				.5138
I	2 (2.0%)	2 (2.9%)	0 (0%)	
II	35 (35.0%)	22 (31.9%)	13 (41.9%)	
III	32 (32.0%)	21 (30.4%)	11 (35.5%)	
IV	15 (15.0%)	13 (18.8%)	2 (6.5%)	
V	14 (14.0%)	10 (14.5%)	4 (12.9%)	
Treatment effect*	2 (2.0%)	1 (1.4%)	1 (3.2%)	
Pathologic T stage and adverse pathologic features				
pT2	44 (44.0%)	33 (47.8%)	11 (35.5%)	.0130
pT3a (ECE)	33 (33.0%)	16 (23.2%)	17 (54.8%)	
pT3b (SVI)	22 (22.0%)	19 (27.5%)	3 (9.7%)	
pT4	1 (1.0%)	1 (1.4%)	0 (0%)	
Bladder neck involvement	10 (10.0%)	7 (10.1%)	3 (9.7%)	1.0
Positive margin	39 (39.0%)	26 (37.7%)	13 (41.9%)	.6867
Tertiary grade 5	17 (17%)	14 (20.2%)	3 (9.7%)	.2555
Baseline sexual function				.2938
Very good/good	16 (16.0%)	13 (18.8%)	3 (9.7%)	
Fair	14 (14.0%)	11 (15.9%)	3 (9.7%)	
Poor/very poor	70 (70.0%)	45 (65.2%)	25 (80.6%)	
Baseline daily pads use				.1669
None	62 (62.0%)	43 (62.3%)	19 (61.3%)	
1	19 (19.0%)	16 (23.2%)	3 (9.7%)	
2	11 (11.0%)	5 (7.2%)	6 (19.4%)	
≥3	8 (8.0%)	5 (7.2%)	3 (9.7%)	
Advanced imaging				.0006
PSMA PET/CT	44 (44.0%)	34 (49.3%)	10 (32.3%)	
^18^F-Fluciclovine PET/CT	32 (32.0%)	14 (20.3%)	18 (58.1%)	
None	24 (24.0%)	21 (30.4%)	3 (9.7%)	
Pre-SBRT PSA				
Mean	0.8	0.7	0.8	.9926
Median (range)	0.3 (0.0-9.3)	0.3 (0.0-6.1)	0.3 (0.1-9.3)	
0-0.2	38 (38.0%)	26 (37.7%)	12 (38.7%)	
0.21-0.49	30 (30.0%)	18 (26.1%)	12 (38.7%)	
0.50-1.0	13 (13.0%)	11 (15.9%)	2 (6.5%)	
>1.0	19 (19.0%)	14 (20.3%)	5 (16.1%)	
Time from prostatectomy to SBRT (mo), median (range)	22.8 (3.8-184.1)	20.3 (3.9-184.1)	25.4 (3.8-178.8)	.6050
SBRT parameters				
Prostate bed dose (Gy), median (range)	32 (30-34)	32 (30-34)	32 (30-34)	.4930
Prostate bed boost use	27 (27.0%)	18 (26.1%)	9 (29.0%)	.7590
Prostate bed boost dose (Gy), median (range)	40 (36.25-40)	40 (36.25-40)	40 (36.25-40)	.0933
Elective nodal RT use	27 (27.0%)	24 (34.8%)	3 (9.7%)	.0136
Elective nodal RT dose (Gy), median (range)	25 (25-25)	25 (25-25)	25 (25-25)	1.0
Gross node boost use	5 (5.0%)	5 (7.2%)	0 (0%)	.3204
Gross node boost dose (Gy), median (range)	40 (35-40)	40 (35-40)	-	-
Concurrent ADT				
ADT use	41 (41.0%)	25 (36.2%)	16 (51.6%)	.1481
Duration (mo), median (range)	6 (1-8)	6 (1-6)	6 (4-8)	.1720

Data are presented as n (%) unless otherwise indicated.

*Abbreviations:* ADT = androgen deprivation therapy; CT = computed tomography; CTgRT = CT-guided radiation therapy; ECE = extraprostatic extension; GG = grade group; MRgRT = MRI-guided radiation therapy; MRI = magnetic resonance imaging; PET = positron emission tomography; PSA = prostate-specific antigen; PSMA = prostate-specific membrane antigen; SBRT = stereotactic body radiation therapy; SVI = seminal vesicle invasion.

* No Gleason score was reported in the surgical pathology specimen due to treatment effect from neoadjuvant androgen deprivation therapy. If a patient underwent both a PSMA PET/CT and a fluciclovine F18 PET/CT, only PSMA PET/CT was counted. *P* values apply to the comparison between CTgRT and MRgRT cohorts, calculated using the Wilcoxon rank sum test for continuous variables and the χ^2^ test or Fisher exact test when appropriate for categorical variables.

**Table 2:** Multivariable analysis of predictors of any CTCAE grade GU, GI, and clinically relevant deterioration in EPIC-26 bowel domain score within 6 months of SBRT

Variable	Any CTCAE grade GU toxicity			Any CTCAE grade GI toxicity			1 × MCID in EPIC-26 bowel score		
	OR	95% CI	*P* value	OR	95% CI	*P* value	OR	95% CI	*P* value
RT platform (CT-versus MRI-guided SBRT)	1.18	0.44-3.16	.7475	3.71	1.38-9.99	**0.0095**	3.02	1.12-8.17	**.0290**
Elective nodal RT (yes versus no)	10.30	2.56-41.43	**.0010**	3.09	0.86-11.12	0.0840	0.65	0.23-1.81	.4077
Prostate bed boost (yes versus no)	-	-	-	0.37	0.12-1.15	0.0858	1.54	0.52-4.58	.4326
Time from RP to SBRT (1-mo increase)	1.00	0.99-1.01	.7033	-	-	-	-	-	-
Baseline pad use (yes versus no)	2.78	1.02-7.61	**.0462**	-	-	-	-	-	-
Baseline IPSS score (1-unit increase)	1.12	1.00-1.25	.0544	-	-	-	-	-	-
Baseline EPIC-26 bowel score (1-unit increase)	-	-	-	0.95	0.89-1.02	0.1428	1.08	1.00-1.16	**.0474**

Values in boldface are statistically significant.

*Abbreviations:* CI = confidence interval; CTCAE = Common Terminology Criteria for Adverse Events; EPIC-26 = Expanded Prostate Cancer Index-26; GI = gastrointestinal; GU = genitourinary; IPSS = International Prostate Symptom Score; MCID = minimal clinically important difference; OR = odds ratio; RP = radical prostatectomy; RT = radiation therapy; SBRT = stereotactic body RT.
