# Supplementary material for: Quality-of-Life Outcomes and Toxicity Profile Among Patients With Localized Prostate Cancer After Radical Prostatectomy Treated With Stereotactic Body Radiation: The SCIMITAR Multicenter Phase 2 Trial
Source: Int J Radiat Oncol Biol Phys. Author manuscript; Available in PMC 2024 Sep 10. (PMC11386273; doi:10.1016/j.ijrobp.2022.08.041)
Supplement: Supplementary Material [file NIHMS2013760-supplement-Supplementary_Material.pdf]

**Supplementary Material**

**Supplementary Tables**

*Table E1.* Clinician-reported toxicity, classified according to Common Terminology Criteria for Adverse Events

*Table E2.* Whole cohort (CT and MRI, n=100) highest grade acute and late treatment-related toxicities

*Table E3.* Acute (≤3 months) highest grade treatment-related toxicities by radiation delivery platforms

*Table E4.* Late (>3 months) highest grade treatment-related toxicities by radiation delivery platforms

*Table E5.* Summary of CTCAE composite GU toxicity

*Table E6.* Summary of CTCAE composite GI toxicity

*Table E7.* Summary of CTCAE composite sexual toxicity

*Table E8.* Summary of CTCAE composite other toxicity

*Table E9.* Descriptive Analysis of Change in Expanded Prostate Cancer Index-26 (EPIC-26) Quality of Life Instrument and International Prostate Symptom Scores (IPSS) in All Available Patients

*Table E10.* Univariable Analysis of GU Toxicities >=2 within 6 months

*Table E11.* Univariable Analysis of Any GU Toxicities within 6 months

*Table E12.* Univariable Analysis of Grade >=2 GI toxicity within first 6 months

*Table E13.* Univariable Analysis of Any grade GI toxicity within first 6 months

*Table E14.* Univariable Analysis of Clinically detectable change (1x MCID) in EPIC urinary incontinence domain within first 6 months

*Table E15.* Univariable Analysis of Clinically detectable change (1x MCID) in EPIC urinary irritative/obstructive domain within first 6 months

*Table E16.* Univariable Analysis of Clinically detectable change (1x MCID) in EPIC bowel domain within first 6 months

*Table E17.* Univariable Analysis of Clinically detectable change (1x MCID) in EPIC sexual domain without ADT within first 6 months

**Supplementary Figures**

*Figure E1.* Isodose distribution for a patient treated by CT-guided SBRT to 32 Gy in 5 fractions to the prostate bed only.

*Figure E2.* Isodose distribution for a patient treated by MRI-guided SBRT to 32 Gy in 5 fractions to the prostate bed only*.*

*Figure E3.* Physician scored genitourinary, gastrointestinal and sexual toxicity over time by radiation delivery platforms.

*Figure E4*. Percentage of clinically relevant deterioration in urinary irritative/obstructive, urinary incontinence, bowel and sexual domain of the Expanded Prostate Cancer Index composite (EPIC-26) questionnaire over time.

**Appendix**

*Appendix E1.* Trial Protocol

**Table E1. Clinician-reported toxicity, classified according to Common Terminology Criteria for Adverse Events**

| | CT-guided SBRT (n=69) | | | | | | | | | | | | | | MRI-guided SBRT (n=31) | | | | | | | | | | | | | |
|---|---|---|---|---|---|---|---|---|---|---|---|---|---|---|---|---|---|---|---|---|---|---|---|---|---|---|---|---|
| | Grade 0 | | Grade 1 | | Grade 2 | | Grade 3 | | Grade ≥1 | | Grade ≥2 | | Grade ≥3 | | Grade 0 | | Grade 1 | | Grade 2 | | Grade 3 | | Grade ≥1 | | Grade ≥2 | | Grade ≥3 | |
| | n | % | n | % | n | % | n | % | n | % | n | % | n | % | n | % | n | % | n | % | n | % | n | % | n | % | n | % |
| **GU toxicity** | | | | | | | | | | | | | | | | | | | | | | | | | | | | |
| Baseline | 66 | 95.7 | 3 | 4.3 | 0 | 0 | 0 | 0 | 3 | 4.3 | 0 | 0 | 0 | 0 | 30 | 96.8 | 1 | 3.2 | 0 | 0 | 0 | 0 | 1 | 3.2 | 0 | 0 | 0 | 0 |
| End of RT | 56 | 81.2 | 13 | 18.8 | 1 | 1.4 | 1 | 1.4 | 13 | 18.8 | 2 | 2.9 | 1 | 1.4 | 24 | 77.4 | 6 | 19.4 | 1 | 3.2 | 0 | 0 | 7 | 22.6 | 1 | 3.2 | 0 | 0 |
| 1 mo FU | 43 | 62.3 | 24 | 34.8 | 4 | 5.8 | 1 | 1.4 | 26 | 37.7 | 5 | 7.2 | 1 | 1.4 | 18 | 58.1 | 12 | 38.7 | 2 | 6.5 | 0 | 0 | 13 | 41.9 | 2 | 6.5 | 0 | 0 |
| 3 mo FU | 36 | 52.2 | 31 | 44.9 | 6 | 8.7 | 1 | 1.4 | 33 | 47.8 | 7 | 10.1 | 1 | 1.4 | 18 | 58.1 | 13 | 41.9 | 2 | 6.5 | 0 | 0 | 13 | 41.9 | 2 | 6.5 | 0 | 0 |
| 6 mo FU | 35 | 50.7 | 32 | 46.4 | 6 | 8.7 | 1 | 1.4 | 34 | 49.3 | 7 | 10.1 | 1 | 1.4 | 18 | 58.1 | 13 | 41.9 | 3 | 9.7 | 0 | 0 | 13 | 41.9 | 3 | 9.7 | 0 | 0 |
| **GI toxicity** | | | | | | | | | | | | | | | | | | | | | | | | | | | | |
| Baseline | 69 | 100 | 0 | 0 | 0 | 0 | 0 | 0 | 0 | 0 | 0 | 0 | 0 | 0 | 31 | 100 | 0 | 0 | 0 | 0 | 0 | 0 | 0 | 0 | 0 | 0 | 0 | 0 |
| End of RT | 58 | 84.1 | 10 | 14.5 | 2 | 2.9 | 0 | 0 | 11 | 15.9 | 2 | 2.9 | 0 | 0 | 26 | 83.9 | 5 | 16.1 | 0 | 0 | 0 | 0 | 5 | 16.1 | 0 | 0 | 0 | 0 |
| 1 mo FU | 41 | 59.4 | 25 | 36.2 | 4 | 5.8 | 1 | 1.4 | 28 | 40.6 | 5 | 7.2 | 1 | 1.4 | 21 | 67.7 | 10 | 32.3 | 0 | 0 | 0 | 0 | 10 | 32.3 | 0 | 0 | 0 | 0 |
| 3 mo FU | 47 | 68.1 | 22 | 31.9 | 1 | 1.4 | 0 | 0 | 22 | 31.9 | 1 | 1.4 | 0 | 0 | 22 | 71.0 | 9 | 29.0 | 0 | 0 | 0 | 0 | 9 | 29.0 | 0 | 0 | 0 | 0 |
| 6 mo FU | 49 | 71.0 | 20 | 29.0 | 1 | 1.4 | 1 | 1.4 | 20 | 29.0 | 1 | 1.4 | 1 | 1.4 | 24 | 77.4 | 7 | 22.6 | 0 | 0 | 0 | 0 | 7 | 22.6 | 0 | 0 | 0 | 0 |
| **Sexual toxicity** | | | | | | | | | | | | | | | | | | | | | | | | | | | | |
| Baseline | 68 | 98.6 | 1 | 1.4 | 0 | 0 | 0 | 0 | 1 | 1.4 | 0 | 0 | 0 | 0 | 31 | 100 | 0 | 0 | 0 | 0 | 0 | 0 | 0 | 0 | 0 | 0 | 0 | 0 |
| End of RT | 68 | 98.6 | 1 | 1.4 | 0 | 0 | 0 | 0 | 1 | 1.4 | 0 | 0 | 0 | 0 | 30 | 96.8 | 1 | 3.2 | 0 | 0 | 0 | 0 | 1 | 3.2 | 0 | 0 | 0 | 0 |
| 1 mo FU | 68 | 98.6 | 1 | 1.4 | 0 | 0 | 0 | 0 | 1 | 1.4 | 0 | 0 | 0 | 0 | 31 | 100 | 0 | 0 | 0 | 0 | 0 | 0 | 0 | 0 | 0 | 0 | 0 | 0 |
| 3 mo FU | 68 | 98.6 | 1 | 1.4 | 0 | 0 | 0 | 0 | 1 | 1.4 | 0 | 0 | 0 | 0 | 31 | 100 | 0 | 0 | 0 | 0 | 0 | 0 | 0 | 0 | 0 | 0 | 0 | 0 |
| 6 mo FU | 68 | 98.6 | 1 | 1.4 | 0 | 0 | 0 | 0 | 1 | 1.4 | 0 | 0 | 0 | 0 | 31 | 100 | 0 | 0 | 0 | 0 | 0 | 0 | 0 | 0 | 0 | 0 | 0 | 0 |

GU: genitourinary; GI: gastrointestinal. Toxicities were graded according to Common Terminology Criteria for Adverse Events (CTCAE) version 4.03. SBRT, Stereotactic Body Radiation Therapy

**Table E2. Whole cohort (CT and MRI, n=100) highest grade acute and late treatment-related toxicities**

| | **Acute (≤3 months) toxicity (n=100)** | | | | | | **Late(>3 months) toxicity (n=100)** | | | | | |
|---|---|---|---|---|---|---|---|---|---|---|---|---|
| | Grade 1 | | Grade 2 | | Grade 3 | | Grade 1 | | Grade 2 | | Grade 3 | |
| Adverse event | n | % | n | % | n | % | n | % | n | % | n | % |
| **Genitourinary** | | | | | | | | | | | | |
| Any GU toxicity* | 51 | 51 | 9 | 9 | 1 | 1 | 48 | 48 | 9 | 9 | 1 | 1 |
| Frequency and/or urgency | 43 | 43 | 5 | 5 | 0 | 0 | 44 | 44 | 4 | 4 | 0 | 0 |
| Dysuria | 9 | 9 | 0 | 0 | 0 | 0 | 6 | 6 | 0 | 0 | 0 | 0 |
| Nocturia | 13 | 13 | 0 | 0 | 0 | 0 | 15 | 15 | 0 | 0 | 0 | 0 |
| Weak stream | 3 | 3 | 0 | 0 | 0 | 0 | 3 | 3 | 0 | 0 | 0 | 0 |
| Pelvic pain | 2 | 2 | 1 | 1 | 0 | 0 | 1 | 1 | 1 | 1 | 0 | 0 |
| Hematuria | 1 | 1 | 0 | 0 | 1 | 1 | 1 | 1 | 0 | 0 | 1 | 1 |
| Incontinence | 8 | 8 | 5 | 5 | 0 | 0 | 6 | 6 | 6 | 6 | 0 | 0 |
| Prostatitis | 1 | 1 | 0 | 0 | 0 | 0 | 0 | 0 | 0 | 0 | 0 | 0 |
| Urinary tract infection | 2 | 2 | 0 | 0 | 0 | 0 | 1 | 1 | 0 | 0 | 0 | 0 |
| **Gastrointestinal** | | | | | | | | | | | | |
| Any GI toxicity* | 61 | 61 | 5 | 5 | 1 | 1 | 35 | 35 | 1 | 1 | 1 | 1 |
| Frequent stools | 14 | 14 | 0 | 0 | 0 | 0 | 10 | 10 | 0 | 0 | 0 | 0 |
| Proctitis | 1 | 1 | 0 | 0 | 0 | 0 | 1 | 1 | 1 | 1 | 0 | 0 |
| Rectal bleeding/hematochezia | 12 | 12 | 0 | 0 | 0 | 0 | 6 | 6 | 0 | 0 | 1 | 1 |
| Hemorrhoid | 1 | 1 | 0 | 0 | 0 | 0 | 0 | 0 | 0 | 0 | 0 | 0 |
| Flatulence/Bloating | 3 | 3 | 0 | 0 | 0 | 0 | 4 | 4 | 0 | 0 | 0 | 0 |
| Constipation | 6 | 6 | 0 | 0 | 0 | 0 | 1 | 1 | 0 | 0 | 0 | 0 |
| Nausea/anorexia/dyspepsia | 3 | 3 | 0 | 0 | 0 | 0 | 2 | 2 | 0 | 0 | 0 | 0 |
| Rectal urgency | 16 | 16 | 0 | 0 | 0 | 0 | 7 | 7 | 0 | 0 | 0 | 0 |
| Rectal pain | 9 | 9 | 0 | 0 | 0 | 0 | 6 | 6 | 1 | 1 | 0 | 0 |
| Fecal incontinence | 2 | 2 | 0 | 0 | 0 | 0 | 2 | 2 | 0 | 0 | 0 | 0 |
| Loose stools/diarrhea | 37 | 37 | 5 | 5 | 1 | 1 | 12 | 12 | 1 | 1 | 0 | 0 |
| Abdominal pain | 5 | 5 | 0 | 0 | 0 | 0 | 3 | 3 | 0 | 0 | 0 | 0 |
| Tenesmus | 6 | 6 | 0 | 0 | 0 | 0 | 6 | 6 | 0 | 0 | 0 | 0 |
| Fistula | 0 | 0 | 0 | 0 | 0 | 0 | 0 | 0 | 0 | 0 | 1 | 1 |
| **Sexual** | | | | | | | | | | | | |
| Any sexual toxicity* | 2 | 2 | 0 | 0 | 0 | 0 | 1 | 1 | 0 | 0 | 0 | 0 |
| Erectile dysfunction | 2 | 2 | 0 | 0 | 0 | 0 | 1 | 1 | 0 | 0 | 0 | 0 |
| **Other events** | | | | | | | | | | | | |
| Any other toxicity* | 25 | 25 | 0 | 0 | 1 | 1 | 20 | 20 | 0 | 0 | 1 | 1 |
| Fatigue | 22 | 22 | 0 | 0 | 0 | 0 | 19 | 19 | 0 | 0 | 0 | 0 |
| Hip/back/joint/muscle pain | 4 | 4 | 0 | 0 | 0 | 0 | 3 | 3 | 0 | 0 | 0 | 0 |
| Stress/Depression | 2 | 2 | 0 | 0 | 0 | 0 | 3 | 3 | 0 | 0 | 0 | 0 |
| Anemia | 0 | 0 | 0 | 0 | 1 | 1 | 0 | 0 | 0 | 0 | 1 | 1 |

GU: genitourinary; GI: gastrointestinal. Toxicities were graded according to Common Terminology Criteria for Adverse Events (CTCAE) version 4.03.

* "Any XXX toxicity" indicates the highest-grade adverse event in that domain for all patients --- patients may have experienced >1 category of adverse event.

**Table E3. Acute (≤3 months) highest grade treatment-related toxicities by radiation delivery platforms**

| | CT-guided SBRT (n=69) | | | | | | MRI-guided SBRT (n=31) | | | | | |
|---|---|---|---|---|---|---|---|---|---|---|---|---|
| | Grade 1 | | Grade 2 | | Grade 3 | | Grade 1 | | Grade 2 | | Grade 3 | |
| Adverse event | n | % | n | % | n | % | n | % | n | % | n | % |
| **Genitourinary** | | | | | | | | | | | | |
| Any GU toxicity* | 37 | 53.6 | 6 | 8.7 | 1 | 1.4 | 14 | 45.2 | 3 | 9.7 | 0 | 0 |
| Frequency and/or urgency | 33 | 47.8 | 2 | 2.9 | 0 | 0 | 10 | 32.3 | 3 | 9.7 | 0 | 0 |
| Dysuria | 8 | 11.6 | 0 | 0 | 0 | 0 | 1 | 3.2 | 0 | 0 | 0 | 0 |
| Nocturia | 8 | 11.6 | 0 | 0 | 0 | 0 | 5 | 16.1 | 0 | 0 | 0 | 0 |
| Weak stream | 3 | 4.3 | 0 | 0 | 0 | 0 | 0 | 0 | 0 | 0 | 0 | 0 |
| Pelvic pain | 1 | 1.4 | 0 | 0 | 0 | 0 | 1 | 3.2 | 1 | 3.2 | 0 | 0 |
| Hematuria | 1 | 1.4 | 0 | 0 | 1 | 1.4 | 0 | 0 | 0 | 0 | 0 | 0 |
| Incontinence | 8 | 11.6 | 5 | 7.2 | 0 | 0 | 0 | 0 | 0 | 0 | 0 | 0 |
| Prostatitis | 1 | 1.4 | 0 | 0 | 0 | 0 | 0 | 0 | 0 | 0 | 0 | 0 |
| Urinary tract infection | 1 | 1.4 | 0 | 0 | 0 | 0 | 1 | 3.2 | 0 | 0 | 0 | 0 |
| **Gastrointestinal** | | | | | | | | | | | | 0 |
| Any GI toxicity* | 48 | 69.6 | 5 | 7.2 | 1 | 1.4 | 13 | 41.9 | 0 | 0 | 0 | 0 |
| Frequent stools | 13 | 18.8 | 0 | 0 | 0 | 0 | 1 | 3.2 | 0 | 0 | 0 | 0 |
| Proctitis | 0 | 0 | 0 | 0 | 0 | 0 | 1 | 3.2 | 0 | 0 | 0 | 0 |
| Rectal bleeding/hematochezia | 12 | 17.4 | 0 | 0 | 0 | 0 | 0 | 0 | 0 | 0 | 0 | 0 |
| Hemorrhoid | 1 | 1.4 | 0 | 0 | 0 | 0 | 0 | 0 | 0 | 0 | 0 | 0 |
| Flatulence/Bloating | 2 | 2.9 | 0 | 0 | 0 | 0 | 1 | 3.2 | 0 | 0 | 0 | 0 |
| Constipation | 6 | 8.7 | 0 | 0 | 0 | 0 | 0 | 0 | 0 | 0 | 0 | 0 |
| Nausea/anorexia/dyspepsia | 2 | 2.9 | 0 | 0 | 0 | 0 | 1 | 3.2 | 0 | 0 | 0 | 0 |
| Rectal urgency | 14 | 20.3 | 0 | 0 | 0 | 0 | 2 | 6.5 | 0 | 0 | 0 | 0 |
| Rectal pain | 7 | 10.1 | 0 | 0 | 0 | 0 | 2 | 6.5 | 0 | 0 | 0 | 0 |
| Fecal incontinence | 1 | 1.4 | 0 | 0 | 0 | 0 | 1 | 3.2 | 0 | 0 | 0 | 0 |
| Loose stools/diarrhea | 26 | 37.7 | 5 | 7.2 | 1 | 1.4 | 11 | 35.5 | 0 | 0 | 0 | 0 |
| Abdominal pain | 5 | 7.2 | 0 | 0 | 0 | 0 | 0 | 0 | 0 | 0 | 0 | 0 |
| Tenesmus | 5 | 7.2 | 0 | 0 | 0 | 0 | 1 | 3.2 | 0 | 0 | 0 | 0 |
| Fistula | 0 | 0 | 0 | 0 | 0 | 0 | 0 | 0 | 0 | 0 | 0 | 0 |
| **Sexual** | | | | | | | | | | | | 0 |
| Any sexual toxicity* | 1 | 1.4 | 0 | 0 | 0 | 0 | 1 | 3.2 | 0 | 0 | 0 | 0 |
| Erectile dysfunction | 1 | 1.4 | 0 | 0 | 0 | 0 | 1 | 3.2 | 0 | 0 | 0 | 0 |
| **Other events** | | | | | | | | | | | | 0 |
| Any other toxicity* | 19 | 27.5 | 0 | 0 | 1 | 1.4 | 6 | 19.4 | 0 | 0 | 0 | 0 |
| Fatigue | 17 | 24.6 | 0 | 0 | 0 | 0 | 5 | 16.1 | 0 | 0 | 0 | 0 |
| Hip/back/joint/muscle pain | 2 | 2.9 | 0 | 0 | 0 | 0 | 2 | 6.5 | 0 | 0 | 0 | 0 |
| Stress/Depression | 2 | 2.9 | 0 | 0 | 0 | 0 | 0 | 0 | 0 | 0 | 0 | 0 |
| Anemia | 0 | 0 | 0 | 0 | 1 | 1.4 | 0 | 0 | 0 | 0 | 0 | 0 |

GU: genitourinary; GI: gastrointestinal. Toxicities were graded according to Common Terminology Criteria for Adverse Events (CTCAE) version 4.03.

* "Any XXX toxicity" indicates the highest-grade adverse event in that domain for all patients --- patients may have experienced >1 category of adverse event.

**Table E4. Late (>3 months) highest grade treatment-related toxicities by radiation delivery platforms**

| | CT-guided SBRT (n=69) | | | | | | MRI-guided SBRT (n=31) | | | | | |
|---|---|---|---|---|---|---|---|---|---|---|---|---|
| | Grade 1 | | Grade 2 | | Grade 3 | | Grade 1 | | Grade 2 | | Grade 3 | |
| Adverse event | n | % | n | % | n | % | n | % | n | % | n | % |
| **Genitourinary** | | | | | | | | | | | | |
| Any GU toxicity* | 35 | 50.7 | 6 | 8.7 | 1 | 1.4 | 13 | 41.9 | 3 | 9.7 | 0 | 0 |
| Frequency and/or urgency | 35 | 50.7 | 2 | 2.9 | 0 | 0 | 9 | 29.0 | 2 | 6.5 | 0 | 0 |
| Dysuria | 5 | 7.2 | 0 | 0 | 0 | 0 | 1 | 3.2 | 0 | 0 | 0 | 0 |
| Nocturia | 10 | 14.5 | 0 | 0 | 0 | 0 | 5 | 16.1 | 0 | 0 | 0 | 0 |
| Weak stream | 3 | 4.3 | 0 | 0 | 0 | 0 | 0 | 0 | 0 | 0 | 0 | 0 |
| Pelvic pain | 1 | 1.4 | 0 | 0 | 0 | 0 | 0 | 0 | 1 | 3.2 | 0 | 0 |
| Hematuria | 1 | 1.4 | 0 | 0 | 1 | 1.4 | 0 | 0 | 0 | 0 | 0 | 0 |
| Incontinence | 6 | 8.7 | 5 | 7.2 | 0 | 0 | 0 | 0 | 1 | 3.2 | 0 | 0 |
| Prostatitis | 0 | 0 | 0 | 0 | 0 | 0 | 0 | 0 | 0 | 0 | 0 | 0 |
| Urinary tract infection | 0 | 0 | 0 | 0 | 0 | 0 | 1 | 3.2 | 0 | 0 | 0 | 0 |
| **Gastrointestinal** | | | | | | | | | | | | |
| Any GI toxicity* | 26 | 37.7 | 1 | 1.4 | 1 | 1.4 | 9 | 29.0 | 0 | 0 | 0 | 0 |
| Frequent stools | 10 | 14.5 | 0 | 0 | 0 | 0 | 0 | 0 | 0 | 0 | 0 | 0 |
| Proctitis | 0 | 0 | 1 | 1.4 | 0 | 0 | 1 | 3.2 | 0 | 0 | 0 | 0 |
| Rectal bleeding/hematochezia | 6 | 8.7 | 0 | 0 | 1 | 1.4 | 0 | 0 | 0 | 0 | 0 | 0 |
| Hemorrhoid | 0 | 0 | 0 | 0 | 0 | 0 | 0 | 0 | 0 | 0 | 0 | 0 |
| Flatulence/Bloating | 3 | 4.3 | 0 | 0 | 0 | 0 | 1 | 3.2 | 0 | 0 | 0 | 0 |
| Constipation | 1 | 1.4 | 0 | 0 | 0 | 0 | 0 | 0 | 0 | 0 | 0 | 0 |
| Nausea/anorexia/dyspepsia | 1 | 1.4 | 0 | 0 | 0 | 0 | 1 | 3.2 | 0 | 0 | 0 | 0 |
| Rectal urgency | 6 | 8.7 | 0 | 0 | 0 | 0 | 1 | 3.2 | 0 | 0 | 0 | 0 |
| Rectal pain | 4 | 5.8 | 1 | 1.4 | 0 | 0 | 2 | 6.5 | 0 | 0 | 0 | 0 |
| Fecal incontinence | 1 | 1.4 | 0 | 0 | 0 | 0 | 1 | 3.2 | 0 | 0 | 0 | 0 |
| Loose stools/diarrhea | 6 | 8.7 | 1 | 1.4 | 0 | 0 | 6 | 19.4 | 0 | 0 | 0 | 0 |
| Abdominal pain | 3 | 4.3 | 0 | 0 | 0 | 0 | 0 | 0 | 0 | 0 | 0 | 0 |
| Tenesmus | 5 | 7.2 | 0 | 0 | 0 | 0 | 1 | 3.2 | 0 | 0 | 0 | 0 |
| Fistula | 0 | 0 | 0 | 0 | 1 | 1.4 | 0 | 0 | 0 | 0 | 0 | 0 |
| **Sexual** | | | | | | | | | | | | |
| Any sexual toxicity* | 1 | 1.4 | 0 | 0 | 0 | 0 | 0 | 0 | 0 | 0 | 0 | 0 |
| Erectile dysfunction | 1 | 1.4 | 0 | 0 | 0 | 0 | 0 | 0 | 0 | 0 | 0 | 0 |
| **Other events** | | | | | | | | | | | | |
| Any other toxicity* | 16 | 23.2 | 0 | 0 | 1 | 1.4 | 4 | 12.9 | 0 | 0 | 0 | 0 |
| Fatigue | 15 | 21.7 | 0 | 0 | 0 | 0 | 4 | 12.9 | 0 | 0 | 0 | 0 |
| Hip/back/joint/muscle pain | 2 | 2.9 | 0 | 0 | 0 | 0 | 1 | 3.2 | 0 | 0 | 0 | 0 |
| Stress/Depression | 3 | 4.3 | 0 | 0 | 0 | 0 | 0 | 0 | 0 | 0 | 0 | 0 |
| Anemia | 0 | 0 | 0 | 0 | 1 | 1.4 | 0 | 0 | 0 | 0 | 0 | 0 |

GU: genitourinary; GI: gastrointestinal. Toxicities were graded according to Common Terminology Criteria for Adverse Events (CTCAE) version 4.03.

* "Any XXX toxicity" indicates the highest-grade adverse event in that domain for all patients --- patients may have experienced >1 category of adverse event.

**Table E5. Summary of CTCAE composite GU toxicity**

| | CT-guided SBRT (n=69) | | MRI-guided SBRT (n=31) | | Statistical comparisons |
|---|---|---|---|---|---|
| **CTCAE genitourinary (GU) composite toxicity** | n | % | n | % | |
| **Baseline** | | | | | |
| Grade 0 | 66 | 95.7 | 30 | 96.8 | |
| Grade 1 | 3 | 4.3 | 1 | 3.2 | p=1.0, Mann-Whitney test comparing grade frequencies |
| Grade 2 | 0 | 0 | 0 | 0 | |
| Grade 3 | 0 | 0 | 0 | 0 | |
| | | | | | **Comparison of Grade X+** |
| **Worst, acute** | | | | | |
| Grade 0 | 31 | 44.9 | 16 | 51.6 | |
| Grade 1 | 31 | 44.9 | 12 | 38.7 | p=0.54 (chi-square) |
| Grade 2 | 6 | 8.7 | 3 | 9.7 | p=0.94 (chi-square) |
| Grade 3 | 1 | 1.4 | 0 | 0 | |
| **Worst, acute, exceeding baseline** | | | | | |
| Baseline not exceeded | 33 | 47.8 | 16 | 51.6 | |
| Grade 1 | 29 | 42.0 | 12 | 38.7 | p=0.73 (chi-square) |
| Grade 2 | 6 | 8.7 | 3 | 9.7 | p=0.94 (chi-square) |
| Grade 3 | 1 | 1.4 | 0 | 0 | |
| **Worst, late** | | | | | |
| Grade 0 | 32 | 46.4 | 18 | 58.1 | |
| Grade 1 | 30 | 43.5 | 10 | 32.3 | p=0.28 (chi-square) |
| Grade 2 | 6 | 8.7 | 3 | 9.7 | p=0.94 (chi-square) |
| Grade 3 | 1 | 1.4 | 0 | 0 | |
| **Worst, late, exceeding baseline** | | | | | |
| Baseline not exceeded | 34 | 49.3 | 18 | 58.1 | |
| Grade 1 | 28 | 40.6 | 10 | 32.3 | p=0.42 (chi-square) |
| Grade 2 | 6 | 8.7 | 3 | 9.7 | p=0.94 (chi-square) |
| Grade 3 | 1 | 1.4 | 0 | 0 | |
| **Worst, up to 6 months** | | | | | |
| Grade 0 | 27 | 39.1 | 16 | 51.6 | |
| Grade 1 | 35 | 50.7 | 11 | 35.5 | p=0.24 (chi-square) |
| Grade 2 | 6 | 8.7 | 4 | 12.9 | p=0.68 (chi-square) |
| Grade 3 | 1 | 1.4 | 0 | 0 | |
| **Worst, up to 6 months, exceeding baseline** | | | | | |
| Baseline not exceeded | 29 | 42.0 | 16 | 51.6 | |
| Grade 1 | 33 | 47.8 | 11 | 35.5 | p=0.37 (chi-square) |
| Grade 2 | 6 | 8.7 | 4 | 12.9 | p=0.68 (chi-square) |
| Grade 3 | 1 | 1.4 | 0 | 0 | |

CTCAE, Common Terminology Criteria for Adverse Events; GU, genitourinary.

**Table E6. Summary of CTCAE composite GI toxicity**

| | CT-guided SBRT (n=69) | | MRI-guided SBRT (n=31) | | Statistical comparisons |
|---|---|---|---|---|---|
| **CTCAE gastrointestinal (GI) composite toxicity** | n | % | n | % | |
| **Baseline** | | | | | |
| Grade 0 | 69 | 100 | 31 | 100 | |
| Grade 1 | 0 | 0 | 0 | 0 | P=1.0, Mann-Whitney test comparing grade frequencies |
| Grade 2 | 0 | 0 | 0 | 0 | |
| Grade 3 | 0 | 0 | 0 | 0 | |
| | | | | | **Comparison of Grade X+** |
| **Worst, acute** | | | | | |
| Grade 0 | 19 | 27.5 | 18 | 58.1 | |
| Grade 1 | 44 | 63.8 | 13 | 41.9 | **Difference 30.5%, 95% CI 11.6%-49.5%, p=0.0056 (chi-square)** |
| Grade 2 | 5 | 7.2 | 0 | 0 | p=0.090 (chi-square) |
| Grade 3 | 1 | 1.4 | 0 | 0 | p=0.50 (chi-square) |
| **Worst, acute, exceeding baseline** | | | | | |
| Baseline not exceeded | 19 | 27.5 | 18 | 58.1 | |
| Grade 1 | 44 | 63.8 | 13 | 41.9 | **Difference 30.5%, 95% CI 11.6%-49.5%, p=0.0056 (chi-square)** |
| Grade 2 | 5 | 7.2 | 0 | 0 | p=0.090 (chi-square) |
| Grade 3 | 1 | 1.4 | 0 | 0 | p=0.50 (chi-square) |
| **Worst, late** | | | | | |
| Grade 0 | 43 | 62.3 | 22 | 71.0 | |
| Grade 1 | 25 | 36.2 | 9 | 29.0 | p=0.40 (chi-square) |
| Grade 2 | 0 | 0 | 0 | 0 | p=0.50 (chi-square) |
| Grade 3 | 1 | 1.4 | 0 | 0 | p=0.50 (chi-square) |
| **Worst, late, exceeding baseline** | | | | | |
| Baseline not exceeded | 43 | 62.3 | 22 | 71.0 | |
| Grade 1 | 25 | 36.2 | 9 | 29.0 | p=0.40 (chi-square) |
| Grade 2 | 0 | 0 | 0 | 0 | p=0.50 (chi-square) |
| Grade 3 | 1 | 1.4 | 0 | 0 | p=0.50 (chi-square) |
| **Worst, up to 6 months** | | | | | |
| Grade 0 | 18 | 26.1 | 18 | 58.1 | |
| Grade 1 | 45 | 65.2 | 13 | 41.9 | **Difference 32.0%, 95% CI 12.9%-51.1%, p=0.0021 (chi-square)** |
| Grade 2 | 4 | 5.8 | 0 | 0 | p=0.09 (chi-square) |
| Grade 3 | 2 | 2.9 | 0 | 0 | p=0.34 (chi-square) |
| **Worst, up to 6 months, exceeding baseline** | | | | | |
| Baseline not exceeded | 18 | 26.1 | 18 | 58.1 | |
| Grade 1 | 45 | 65.2 | 13 | 41.9 | **Difference 32.0%, 95% CI 12.9%-51.1%, p=0.0021 (chi-square)** |
| Grade 2 | 4 | 5.8 | 0 | 0 | p=09 (chi-square) |
| Grade 3 | 2 | 2.9 | 0 | 0 | p=0.34 (chi-square) |

CTCAE, Common Terminology Criteria for Adverse Events; GI, gastrointestinal.

**Table E7. Summary of CTCAE composite sexual toxicity**

| | CT-guided SBRT (n=69) | | MRI-guided SBRT (n=31) | | Statistical comparisons |
|---|---|---|---|---|---|
| **CTCAE sexual composite toxicity** | n | % | n | % | |
| **Baseline** | | | | | |
| Grade 0 | 68 | 98.6 | 31 | 100 | |
| Grade 1 | 1 | 1.4 | 0 | 0 | p=1.0, Mann-Whitney test comparing grade frequencies |
| Grade 2 | 0 | 0 | 0 | 0 | |
| Grade 3 | 0 | 0 | 0 | 0 | |
| | | | | | **Comparison of Grade X+** |
| **Worst, acute** | | | | | |
| Grade 0 | 68 | 98.6 | 30 | 96.8 | |
| Grade 1 | 1 | 1.4 | 1 | 3.2 | p=0.56 (chi-square) |
| Grade 2 | 0 | 0 | 0 | 0 | |
| Grade 3 | 0 | 0 | 0 | 0 | |
| **Worst, acute, exceeding baseline** | | | | | |
| Baseline not exceeded | 69 | 100 | 30 | 96.8 | |
| Grade 1 | 0 | 0 | 1 | 3.2 | p=0.13 (chi-square) |
| Grade 2 | 0 | 0 | 0 | 0 | |
| Grade 3 | 0 | 0 | 0 | 0 | |
| **Worst, late** | | 0 | | 0 | |
| Grade 0 | 68 | 98.6 | 31 | 100 | |
| Grade 1 | 0 | 0 | 0 | 0 | |
| Grade 2 | 0 | 0 | 0 | 0 | |
| Grade 3 | 0 | 0 | 0 | 0 | |
| **Worst, late, exceeding baseline** | | | | | |
| Baseline not exceeded | 69 | 100 | 31 | 100 | |
| Grade 1 | 0 | 0 | | 0 | |
| Grade 2 | 0 | 0 | | 0 | |
| Grade 3 | 0 | 0 | | 0 | |
| **Worst, up to 6 months** | | | | | |
| Grade 0 | 68 | 98.6 | 30 | 96.8 | |
| Grade 1 | 1 | 1.4 | 0 | 0 | p=0.50 (chi-square) |
| Grade 2 | 0 | 0 | 0 | 0 | |
| Grade 3 | 0 | 0 | 0 | 0 | |
| **Worst, up to 6 months, exceeding baseline** | | | | | |
| Baseline not exceeded | 69 | 100 | 30 | 96.8 | |
| Grade 1 | 0 | 0 | 1 | 3.2 | p=0.13 (chi-square) |
| Grade 2 | 0 | 0 | 0 | 0 | |
| Grade 3 | 0 | 0 | 0 | 0 | |

CTCAE, Common Terminology Criteria for Adverse Events

**Table E8. Summary of CTCAE composite other toxicity**

| | CT-guided SBRT (n=69) | | MRI-guided SBRT (n=31) | | Statistical comparisons |
|---|---|---|---|---|---|
| **CTCAE other composite toxicity** | n | % | n | % | |
| **Baseline** | | | | | |
| Grade 0 | 68 | 98.6 | 30 | 96.8 | p=1.0, Mann-Whitney test comparing grade frequencies |
| Grade 1 | 1 | 1.4 | 1 | 3.2 | |
| Grade 2 | 0 | 0 | 0 | 0 | |
| Grade 3 | 0 | 0 | 0 | 0 | |
| | | | | | **Comparison of Grade X+** |
| **Worst, acute** | | | | | |
| Grade 0 | 50 | 72.5 | 25 | 80.6 | |
| Grade 1 | 18 | 26.1 | 6 | 19.4 | p=0.38 (chi-square) |
| Grade 2 | 0 | 0 | 0 | 0 | p=0.50 (chi-square) |
| Grade 3 | 1 | 1.4 | 0 | 0 | p=0.50 (chi-square) |
| **Worst, acute, exceeding baseline** | | | | | |
| Baseline not exceeded | 51 | 73.9 | 26 | 83.9 | |
| Grade 1 | 17 | 24.6 | 5 | 16.1 | p=0.27 (chi-square) |
| Grade 2 | 0 | 0 | 0 | 0 | p=0.50 (chi-square) |
| Grade 3 | 1 | 1.4 | 0 | 0 | p=0.50 (chi-square) |
| **Worst, late** | | | | | |
| Grade 0 | 53 | 76.8 | 27 | 87.1 | |
| Grade 1 | 15 | 21.7 | 4 | 12.9 | p=0.23 (chi-square) |
| Grade 2 | 0 | 0 | 0 | 0 | p=0.50 (chi-square) |
| Grade 3 | 1 | 1.4 | 0 | 0 | p=0.50 (chi-square) |
| **Worst, late, exceeding baseline** | | | | | |
| Baseline not exceeded | 53 | 76.8 | 27 | 87.1 | |
| Grade 1 | 15 | 21.7 | 4 | 12.9 | p=0.23 (chi-square) |
| Grade 2 | 0 | 0 | 0 | 0 | p=0.50 (chi-square) |
| Grade 3 | 1 | 1.4 | 0 | 0 | p=0.50 (chi-square) |
| **Worst, up to 6 months** | | | | | |
| Grade 0 | 50 | 72.5 | 25 | 80.6 | |
| Grade 1 | 18 | 26.1 | 6 | 19.4 | p=0.38 (chi-square) |
| Grade 2 | 0 | 0 | 0 | 0 | p=0.50 (chi-square) |
| Grade 3 | 1 | 1.4 | 0 | 0 | p=0.50 (chi-square) |
| **Worst, up to 6 months, exceeding baseline** | | | | | |
| Baseline not exceeded | 50 | 72.5 | 26 | 83.9 | |
| Grade 1 | 18 | 26.1 | 5 | 16.1 | p=0.38 (chi-square) |
| Grade 2 | 0 | 0 | 0 | 0 | p=0.50 (chi-square) |
| Grade 3 | 1 | 1.4 | 0 | 0 | p=0.50 (chi-square) |

Other toxicities refer to toxicities other than GU, GI or sexual. Examples include fatigue, hip/back/joint/muscle pain, stress/depression and anemia. CTCAE, Common Terminology Criteria for Adverse Events

**Table E9. Descriptive Analysis of Change in Expanded Prostate Cancer Index-26 (EPIC-26) Quality of Life Instrument and International Prostate Symptom Scores (IPSS) in All Available Patients[a]**

| | Baseline[b] | | | 1 Month[c] | | | 3 Months[c] | | | 6 Months[c] | | |
|---|---|---|---|---|---|---|---|---|---|---|---|---|
| **Scale** | No. | Mean | SD | No. | Mean | SD | No. | Mean | SD | No. | Mean | SD |
| **EPIC-26** | | | | | | | | | | | | |
| Urinary Irritative/Obstructive | 95 | 90.53 | 10.47 | 91 | -2.88 | 9.74 | 90 | -0.20 | 8.43 | 93 | -2.15 | 12.24 |
| Urinary Incontinence | 94 | 67.61 | 27.88 | 90 | 3.64 | 13.16 | 89 | 4.31 | 17.32 | 91 | 1.28 | 17.23 |
| Bowel | 92 | 96.46 | 7.85 | 89 | -8.60 | 18.14 | 87 | -2.59 | 10.44 | 88 | -4.58 | 18.09 |
| Sexual[d] | 65 | 35.93 | 29.75 | 50 | -1.07 | 11.16 | 48 | 2.41 | 11.46 | 48 | 1.72 | 15.94 |
| **IPSS** | | | | | | | | | | | | |
| IPSS_S[e] | 97 | 6.21 | 5.03 | 95 | 1.24 | 4.80 | 95 | 0.03 | 4.41 | 96 | -0.06 | 4.15 |
| IPSS_QOL[f] | 97 | 1.78 | 1.58 | 95 | 0.19 | 1.22 | 95 | 0.09 | 1.25 | 96 | 0.08 | 1.35 |

[a] Decreasing number of available patients with subsequent times after baseline are die to missed questionnaires, loss to follow-up and death.

[b] The numbers represent the absolute score in each domain at baseline.

[c] The numbers represent *change* in scores in respective domains at the specified time points compared to the baseline.

[d] In patients not receiving concurrent androgen deprivation therapy

[e] Summary of the first 7 questions of the IPSS questionnaire (incomplete emptying, frequency, intermittency, urgency, weak stream and nocturia). Higher number means worse urinary symptoms.

[f] Score of the quality of life question related to urinary symptoms in the IPSS questionnaire. A score of 0 is “delighted” and 6 is “terrible”.

No., number; CI, confidence interval; SD, standard deviation; QOL, quality of life; EPIC-26, Expanded Prostate Cancer Index-26; IPSS, International Prostate Symptom Score

## Table E10. Univariable Analysis of GU Toxicities >=2 within 6 months

| Variable<br>Statistic or Category | <2 GU Toxicities<br>(n=89) | >=2 GU Toxicities<br>(n=11) | P Value |
|---|---|---|---|
| RT Platform | | | |
| CT | 62 (69.7%) | 7 (63.6%) | 0.7346 |
| MRI | 27 (30.3%) | 4 (36.4%) | . |
| Age | | | |
| n | 89 | 11 | 0.2243 |
| Mean (SD) | 67.3 (6.38) | 65.5 (6.77) | . |
| Median | 69.0 (64.00, 71.00) | 65.0 (62.00, 69.00) | . |
| Min, Max | 49, 82 | 53, 78 | . |
| Baseline IPSS | | | |
| n | 86 | 11 | 0.0103 |
| Mean (SD) | 5.7 (4.73) | 10.1 (5.82) | . |
| Median | 4.5 (2.00, 8.00) | 10.0 (6.00, 15.00) | . |
| Min, Max | 0, 19 | 0, 21 | . |
| Elective nodal RT | | | |
| No | 67 (75.3%) | 6 (54.5%) | 0.1614 |
| Yes | 22 (24.7%) | 5 (45.5%) | . |
| Prostate bed boost | | | |
| No | 65 (73.0%) | 8 (72.7%) | 1.0000 |
| Yes | 24 (27.0%) | 3 (27.3%) | . |
| ADT Use | | | |
| No | 55 (61.8%) | 4 (36.4%) | 0.1191 |
| Yes | 34 (38.2%) | 7 (63.6%) | . |
| Baseline pad use | | | |
| No | 56 (65.1%) | 3 (27.3%) | 0.0219 |
| Yes | 30 (34.9%) | 8 (72.7%) | . |
| Missing | 3 | | . |
| Time from RP to SBRT | | | |
| n | 89 | 11 | 0.0101 |
| Mean (SD) | 44.028 (45.9251) | 26.588 (51.1745) | . |
| Median | 25.085 (11.4080, 53.1620) | 8.121 (6.0160, 20.3180) | . |

## Table E10. Univariable Analysis of GU Toxicities >=2 within 6 months

| Variable<br>Statistic or Category | <2 GU Toxicities<br>(n=89) | >=2 GU Toxicities<br>(n=11) | P Value |
|---|---|---|---|
| Min, Max | 3.91, 184.11 | 3.85, 178.85 | . |

Note: P Values obtained using Fishers exact test for categorical variables and Wilcoxon Rank-sum test for continous variables

## Table E11. Univariable Analysis of Any GU Toxicities within 6 months

| Variable<br>Statistic or Category | No GU Toxicities<br>(n=43) | Any GU Toxicities<br>(n=57) | P Value |
|---|---|---|---|
| RT Platform | | | |
| CT | 27 (62.8%) | 42 (73.7%) | 0.2436 |
| MRI | 16 (37.2%) | 15 (26.3%) | . |
| Age | | | |
| n | 43 | 57 | 0.6385 |
| Mean (SD) | 66.8 (6.10) | 67.4 (6.68) | . |
| Median | 67.0 (64.00, 70.00) | 69.0 (63.00, 72.00) | . |
| Min, Max | 52, 82 | 49, 81 | . |
| Baseline IPSS | | | |
| n | 40 | 57 | 0.0099 |
| Mean (SD) | 4.7 (3.50) | 7.3 (5.64) | . |
| Median | 3.0 (2.50, 7.50) | 7.0 (2.00, 10.00) | . |
| Min, Max | 0, 14 | 0, 21 | . |
| Elective nodal RT | | | |
| No | 39 (90.7%) | 34 (59.6%) | 0.0005 |
| Yes | 4 (9.3%) | 23 (40.4%) | . |
| Prostate bed boost | | | |
| No | 34 (79.1%) | 39 (68.4%) | 0.2350 |
| Yes | 9 (20.9%) | 18 (31.6%) | . |
| ADT Use | | | |
| No | 29 (67.4%) | 30 (52.6%) | 0.1360 |
| Yes | 14 (32.6%) | 27 (47.4%) | . |
| Baseline pad use | | | |
| No | 30 (75.0%) | 29 (50.9%) | 0.0166 |
| Yes | 10 (25.0%) | 28 (49.1%) | . |
| Missing | 3 | | . |
| Time from RP to SBRT | | | |
| n | 43 | 57 | 0.7225 |
| Mean (SD) | 43.689 (50.7919) | 40.919 (43.5537) | . |
| Median | 22.553 (10.0270, 48.7560) | 22.948 (8.2850, 52.4050) | . |

## Table E11. Univariable Analysis of Any GU Toxicities within 6 months

| Variable<br>Statistic or Category | No GU Toxicities<br>(n=43) | Any GU Toxicities<br>(n=57) | P Value |
|---|---|---|---|
| Min, Max | 4.67, 184.11 | 3.85, 178.85 | . |

Note: P values obtained using Chi-square test for categorical variables and Student's t-test for continuous variables with the exception of time from RP to SBRT which was analyzed using Wilcoxon rank-sum test.

**Table E12. Univariable Analysis of Grade >=2 GI toxicity within first 6 months**

| Variable<br>Statistic or Category | No Grade >=2 GI toxicity within first 6 months<br>(n=94) | Grade >=2 GI toxicity within first 6 months<br>(n=6) | P Value |
|---|---|---|---|
| RT Platform | | | |
| CT | 63 (67.0%) | 6 (100.0%) | 0.1729 |
| MRI | 31 (33.0%) | 0 (0.0%) | . |
| Age | | | |
| n | 94 | 6 | 0.9652 |
| Mean (SD) | 67.1 (6.46) | 67.5 (6.16) | . |
| Median | 68.0 (63.00, 71.00) | 66.5 (64.00, 73.00) | . |
| Min, Max | 49, 82 | 59, 76 | . |
| Elective nodal RT | | | |
| No | 71 (75.5%) | 2 (33.3%) | 0.0439 |
| Yes | 23 (24.5%) | 4 (66.7%) | . |
| Prostate bed boost | | | |
| No | 68 (72.3%) | 5 (83.3%) | 1.0000 |
| Yes | 26 (27.7%) | 1 (16.7%) | . |
| ADT Use | | | |
| No | 55 (58.5%) | 4 (66.7%) | 1.0000 |
| Yes | 39 (41.5%) | 2 (33.3%) | . |
| Time from RP to SBRT | | | |
| n | 94 | 6 | 0.3721 |
| Mean (SD) | 40.651 (45.0953) | 64.970 (66.7967) | . |
| Median | 21.649 (8.7450, 48.7560) | 44.219 (16.5040, 96.8550) | . |
| Min, Max | 3.85, 178.85 | 3.91, 184.11 | . |
| Baseline EPIC bowel domain score | | | |
| n | 87 | 5 | 0.3980 |
| Mean (SD) | 96.300 (8.0373) | 99.167 (1.8634) | . |
| Median | 100.000 (95.8330, 100.0000) | 100.000 (100.0000, 100.0000) | . |
| Min, Max | 54.17, 119.80 | 95.83, 100.00 | . |

Note: P Values obtained using Fisher's exact test for categorical variables and Wilcoxon rank-sum test for continous variables

## Table E13. Univariable Analysis of Any grade GI toxicity within first 6 months

| Variable<br>Statistic or Category | No Any grade GI toxicity within first 6 months<br>(n=36) | Any grade GI toxicity within first 6 months<br>(n=64) | P Value |
|---|---|---|---|
| RT Platform | | | |
| CT | 18 (50.0%) | 51 (79.7%) | 0.0021 |
| MRI | 18 (50.0%) | 13 (20.3%) | . |
| Age | | | |
| n | 36 | 64 | 0.7706 |
| Mean (SD) | 66.9 (6.34) | 67.3 (6.50) | . |
| Median | 68.0 (63.00, 70.50) | 68.5 (64.00, 71.00) | . |
| Min, Max | 53, 82 | 49, 81 | . |
| Elective nodal RT | | | |
| No | 31 (86.1%) | 42 (65.6%) | 0.0268 |
| Yes | 5 (13.9%) | 22 (34.4%) | . |
| Prostate bed boost | | | |
| No | 22 (61.1%) | 51 (79.7%) | 0.0446 |
| Yes | 14 (38.9%) | 13 (20.3%) | . |
| ADT Use | | | |
| No | 20 (55.6%) | 39 (60.9%) | 0.5994 |
| Yes | 16 (44.4%) | 25 (39.1%) | . |
| Time from RP to SBRT | | | |
| n | 36 | 64 | 0.9771 |
| Mean (SD) | 47.494 (57.0747) | 39.081 (39.6661) | . |
| Median | 20.910 (9.7480, 55.8740) | 24.016 (8.7290, 49.3640) | . |
| Min, Max | 4.67, 178.85 | 3.85, 184.11 | . |
| Baseline EPIC bowel domain score | | | |
| n | 32 | 60 | 0.0171 |
| Mean (SD) | 97.656 (6.8537) | 95.816 (8.3172) | . |
| Median | 100.000 (100.0000, 100.0000) | 100.000 (91.6670, 100.0000) | . |
| Min, Max | 70.83, 100.00 | 54.17, 119.80 | . |

Note: P Values obtained using Chi-square test for categorical variables and Student's t-test test for continous variables except for EPIC score and time from RP to SBRT which was analyzed using Wilcoxon rank-sum

**Table E14. Univariable Analysis of Clinically detectable change (1x MCID) in EPIC urinary incontinence domain within first 6 months**

| Variable<br>Statistic or Category | No Clinically detectable change (1x MCID) in EPIC urinary incontinence domain within first 6 months<br>(n=60) | Clinically detectable change (1x MCID) in EPIC urinary incontinence domain within first 6 months<br>(n=33) | P Value |
|---|---|---|---|
| RT Platform | | | |
| CT | 38 (63.3%) | 25 (75.8%) | 0.2201 |
| MRI | 22 (36.7%) | 8 (24.2%) | . |
| Age | | | |
| n | 60 | 33 | 0.6255 |
| Mean (SD) | 67.0 (6.94) | 67.7 (5.81) | . |
| Median | 68.0 (63.00, 71.00) | 69.0 (64.00, 72.00) | . |
| Min, Max | 49, 82 | 54, 78 | . |
| Elective nodal RT | | | |
| No | 46 (76.7%) | 22 (66.7%) | 0.2980 |
| Yes | 14 (23.3%) | 11 (33.3%) | . |
| Prostate bed boost | | | |
| No | 44 (73.3%) | 24 (72.7%) | 0.9497 |
| Yes | 16 (26.7%) | 9 (27.3%) | . |
| ADT Use | | | |
| No | 36 (60.0%) | 21 (63.6%) | 0.7305 |
| Yes | 24 (40.0%) | 12 (36.4%) | . |
| Baseline Pad Use | | | |
| No | 35 (58.3%) | 21 (63.6%) | 0.6171 |
| Yes | 25 (41.7%) | 12 (36.4%) | . |
| Missing | | | . |
| Time from RP to SBRT | | | |
| n | 60 | 33 | 0.6733 |
| Mean (SD) | 37.311 (43.6415) | 39.252 (41.7428) | . |
| Median | 20.532 (8.5480, 44.7950) | 19.989 (10.1260, 52.4050) | . |
| Min, Max | 3.91, 184.11 | 4.67, 178.85 | . |
| Baseline EPIC urinary incontinence domain score | | | |
| n | 60 | 33 | 0.3718 |

**Table E14. Univariable Analysis of Clinically detectable change (1x MCID) in EPIC urinary incontinence domain within first 6 months**

| Variable<br>Statistic or Category | No Clinically detectable change (1x MCID) in EPIC urinary incontinence domain within first 6 months (n=60) | Clinically detectable change (1x MCID) in EPIC urinary incontinence domain within first 6 months (n=33) | P Value |
|---|---|---|---|
| Mean (SD) | 64.821 (31.0838) | 72.341 (20.8574) | . |
| Median | 66.750 (38.6250, 96.8750) | 73.000 (58.5000, 91.7500) | . |
| Min, Max | 0.00, 100.00 | 16.50, 100.00 | . |

Note: P Values obtained using Chi-square test for categorical variables and Student's t-test test for continous variables except for time and EPIC score which was analyzed using Wilcoxon rank-sum

**Table E15. Univariable Analysis of Clinically detectable change (1x MCID) in EPIC urinary irritative/obstructive domain within first 6 months**

| Variable<br>Statistic or Category | No Clinically detectable change (1x MCID) in EPIC urinary irritative/obstructive domain within first 6 months<br>(n=47) | Clinically detectable change (1x MCID) in EPIC urinary irritative/obstructive domain within first 6 months<br>(n=47) | P Value |
|---|---|---|---|
| RT Platform | | | |
| CT | 29 (61.7%) | 35 (74.5%) | 0.1843 |
| MRI | 18 (38.3%) | 12 (25.5%) | . |
| Age | | | |
| n | 47 | 47 | 0.7898 |
| Mean (SD) | 67.5 (6.22) | 67.1 (6.88) | . |
| Median | 69.0 (63.00, 71.00) | 68.0 (63.00, 72.00) | . |
| Min, Max | 49, 81 | 53, 82 | . |
| Elective nodal RT | | | |
| No | 38 (80.9%) | 31 (66.0%) | 0.1022 |
| Yes | 9 (19.1%) | 16 (34.0%) | . |
| Prostate bed boost | | | |
| No | 37 (78.7%) | 31 (66.0%) | 0.1665 |
| Yes | 10 (21.3%) | 16 (34.0%) | . |
| ADT Use | | | |
| No | 33 (70.2%) | 24 (51.1%) | 0.0574 |
| Yes | 14 (29.8%) | 23 (48.9%) | . |
| Baseline Pad Use | | | |
| No | 30 (63.8%) | 26 (55.3%) | 0.4005 |
| Yes | 17 (36.2%) | 21 (44.7%) | . |
| Missing | | | . |
| Time from RP to SBRT | | | |
| n | 47 | 47 | 0.5655 |
| Mean (SD) | 38.080 (40.5030) | 42.461 (50.1226) | . |
| Median | 22.981 (12.0000, 48.2960) | 18.805 (8.5480, 52.4050) | . |
| Min, Max | 5.65, 184.11 | 3.91, 178.85 | . |

**Table E15. Univariable Analysis of Clinically detectable change (1x MCID) in EPIC urinary irritative/obstructive domain within first 6 months**

| Variable<br>Statistic or Category | No Clinically detectable change (1x MCID) in EPIC urinary irritative/obstructive domain within first 6 months<br>(n=47) | Clinically detectable change (1x MCID) in EPIC urinary irritative/obstructive domain within first 6 months<br>(n=47) | P Value |
|---|---|---|---|
| Baseline EPIC urinary irritative/obstructive domain score | | | |
| n | 47 | 47 | 0.8171 |
| Mean (SD) | 90.293 (10.6520) | 90.957 (10.4101) | . |
| Median | 93.750 (87.5000, 100.0000) | 93.750 (87.5000, 100.0000) | . |
| Min, Max | 62.50, 100.00 | 50.00, 100.00 | . |

Note: P Values obtained using Chi-square test for categorical variables and Student's t-test test for continous variables except for time and EPIC score which was analyzed using Wilcoxon rank-sum

**Table E16. Univariable Analysis of Clinically detectable change (1x MCID) in EPIC bowel domain within first 6 months**

| Variable<br>Statistic or Category | No Clinically detectable change (1x MCID) in EPIC bowel domain within first 6 months (n=40) | Clinically detectable change (1x MCID) in EPIC bowel domain within first 6 months (n=51) | P Value |
|---|---|---|---|
| RT Platform | | | |
| CT | 22 (55.0%) | 38 (74.5%) | 0.0513 |
| MRI | 18 (45.0%) | 13 (25.5%) | . |
| Age | | | |
| n | 40 | 51 | 0.0970 |
| Mean (SD) | 68.2 (6.78) | 65.9 (6.18) | . |
| Median | 69.0 (63.50, 73.50) | 67.0 (63.00, 70.00) | . |
| Min, Max | 53, 82 | 49, 78 | . |
| Elective nodal RT | | | |
| No | 29 (72.5%) | 37 (72.5%) | 0.9959 |
| Yes | 11 (27.5%) | 14 (27.5%) | . |
| Prostate bed boost | | | |
| No | 31 (77.5%) | 39 (76.5%) | 0.9079 |
| Yes | 9 (22.5%) | 12 (23.5%) | . |
| ADT Use | | | |
| No | 26 (65.0%) | 30 (58.8%) | 0.5478 |
| Yes | 14 (35.0%) | 21 (41.2%) | . |
| Time from RP to SBRT | | | |
| n | 40 | 51 | 0.3293 |
| Mean (SD) | 44.601 (50.0174) | 37.107 (43.3717) | . |
| Median | 24.214 (10.7010, 50.9590) | 17.589 (8.2850, 48.2960) | . |
| Min, Max | 5.39, 184.11 | 3.85, 178.85 | . |
| Baseline EPIC bowel domain score | | | |
| n | 40 | 51 | 0.1187 |
| Mean (SD) | 94.583 (9.7694) | 97.856 (5.7152) | . |
| Median | 100.000 (91.6670, 100.0000) | 100.000 (95.8330, 100.0000) | . |

**Table E16. Univariable Analysis of Clinically detectable change (1x MCID) in EPIC bowel domain within first 6 months**

| Variable<br>Statistic or Category | No Clinically detectable change (1x MCID) in EPIC bowel domain within first 6 months<br>(n=40) | Clinically detectable change (1x MCID) in EPIC bowel domain within first 6 months<br>(n=51) | P Value |
|---|---|---|---|
| Min, Max | 54.17, 100.00 | 83.33, 119.80 | . |

Note: P Values obtained using Chi-square test for categorical variables and Student's t-test test for continous variables except for EPIC score and time from RP to SBRT which was analyzed using Wilcoxon rank-sum

**Table E17. Univariable Analysis of Clinically detectable change (1x MCID) in EPIC sexual domain without ADT within first 6 months**

| Variable<br>Statistic or Category | No Clinically detectable change (1x MCID) in EPIC sexual domain without ADT within first 6 months (n=58) | Clinically detectable change (1x MCID) in EPIC sexual domain without ADT within first 6 months (n=26) | P Value |
|---|---|---|---|
| RT Platform | | | |
| CT | 39 (67.2%) | 18 (69.2%) | 0.8568 |
| MRI | 19 (32.8%) | 8 (30.8%) | . |
| Age | | | |
| n | 58 | 26 | 0.5021 |
| Mean (SD) | 66.9 (7.19) | 67.9 (4.82) | . |
| Median | 68.0 (63.00, 71.00) | 68.5 (64.00, 72.00) | . |
| Min, Max | 49, 82 | 58, 77 | . |
| Elective nodal RT | | | |
| No | 43 (74.1%) | 18 (69.2%) | 0.6410 |
| Yes | 15 (25.9%) | 8 (30.8%) | . |
| Prostate bed boost | | | |
| No | 44 (75.9%) | 17 (65.4%) | 0.3195 |
| Yes | 14 (24.1%) | 9 (34.6%) | . |
| ADT Use | | | |
| No | 40 (69.0%) | 10 (38.5%) | 0.0085 |
| Yes | 18 (31.0%) | 16 (61.5%) | . |
| ED meds/device | | | |
| No | 29 (52.7%) | 16 (66.7%) | 0.2498 |
| Yes | 26 (47.3%) | 8 (33.3%) | . |
| Missing | 3 | 2 | . |
| Time from RP to SBRT | | | |
| n | 58 | 26 | 0.5262 |
| Mean (SD) | 39.073 (46.3487) | 49.084 (53.9328) | . |
| Median | 20.367 (8.5480, 47.2110) | 22.636 (8.7120, 57.2380) | . |
| Min, Max | 3.91, 184.11 | 5.00, 173.95 | . |
| Baseline EPIC sexual function domain score | | | |
| n | 58 | 26 | <.0001 |

**Table E17. Univariable Analysis of Clinically detectable change (1x MCID) in EPIC sexual domain without ADT within first 6 months**

| Variable<br>Statistic or Category | No Clinically detectable change (1x MCID) in EPIC sexual domain without ADT within first 6 months (n=58) | Clinically detectable change (1x MCID) in EPIC sexual domain without ADT within first 6 months (n=26) | P Value |
|---|---|---|---|
| Mean (SD) | 26.043 (26.1381) | 52.301 (30.6967) | . |
| Median | 16.667 (8.3330, 38.8330) | 46.583 (22.1670, 83.3330) | . |
| Min, Max | 0.00, 100.00 | 16.67, 100.00 | . |

Note: P Values obtained using Chi-square test for categorical variables and Student's t-test test for continous variables except for time and EPIC score which was analyzed using Wilcoxon rank-sum

**Figure E1**

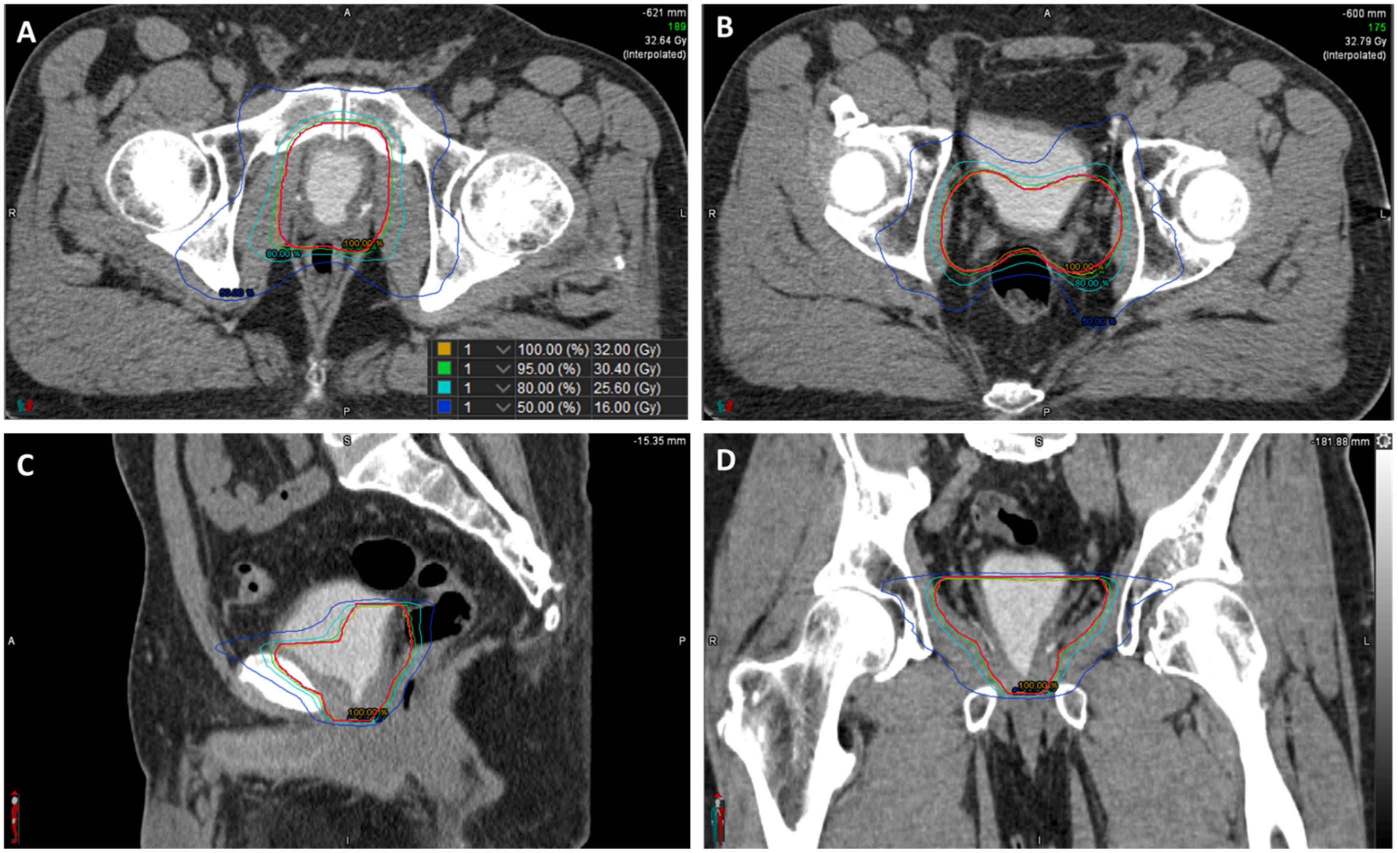


**Figure E1. Isodose distribution for a patient treated by CT-guided SBRT to 32 Gy in 5 fractions to the prostate bed only.** Representative images in the axial plane below the pubic symphysis (A), axial plane above the pubic symphysis (B), sagittal plane (C) and coronal plane (D) are shown. Prostate bed PTV contour is shown in red. A 5mm isotropic expansion from the CTV was used to from the PTV. Isodose lines corresponds to 100% dose (32 Gy, yellow), 95% dose (30.4 Gy, green), 80% dose (25.6 Gy, cyan) and 50% dose (16 Gy, dark blue). PTV, planning target volume.

**Figure E2**

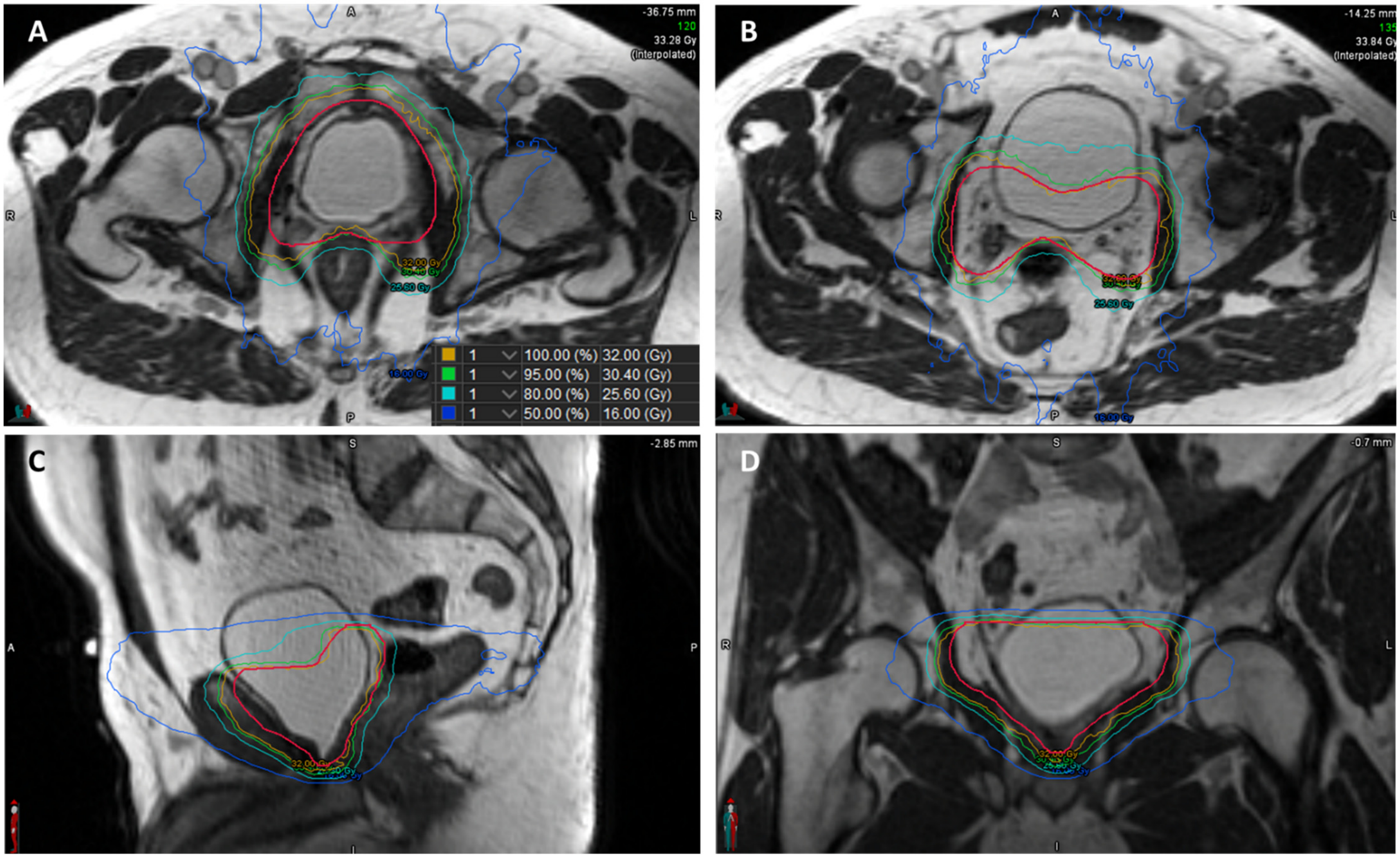


**Figure E2. Isodose distribution for a patient treated by MRI-guided SBRT to 32 Gy in 5 fractions to the prostate bed only.** Representative images in the axial plane below the pubic symphysis (A), axial plane above the pubic symphysis (B), sagittal plane (C) and coronal plane (D) are shown. Prostate bed PTV contour is shown in red. A 3mm isotropic expansion from the CTV was used to from the PTV. Isodose lines corresponds to 100% dose (32 Gy, yellow), 95% dose (30.4 Gy, green), 80% dose (25.6 Gy, cyan) and 50% dose (16 Gy, dark blue). PTV, planning target volume.

**Figure E3**

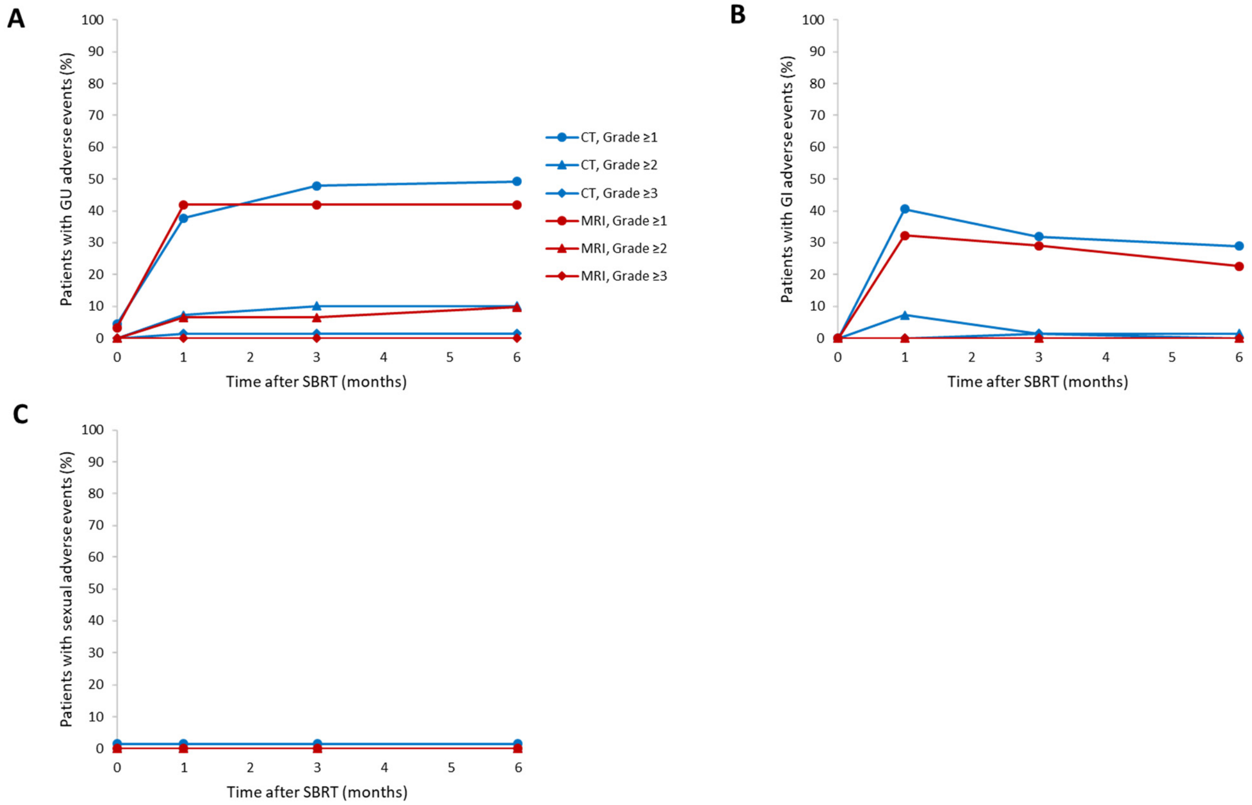


**Figure E3. Physician scored genitourinary, gastrointestinal and sexual toxicity over time by radiation delivery platforms.** Physician-score toxicities were graded according to Common Terminology Criteria for Adverse Events version 4.03. SBRT, stereotactic radiation therapy.

## Figure E4

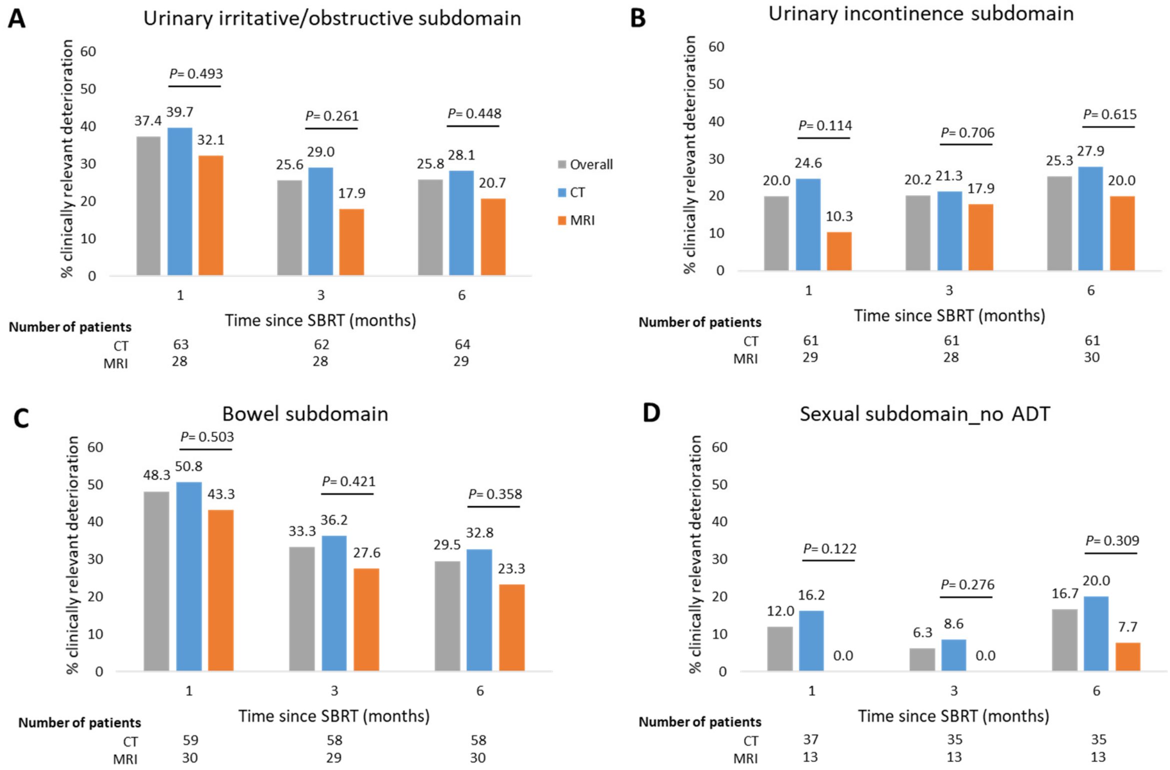


**Figure E4. Percentage of clinically relevant deterioration in urinary irritative/obstructive, urinary incontinence, bowel and sexual domain of the Expanded Prostate Cancer Index composite (EPIC-26) questionnaire over time.** For panel D, only patients without concurrent androgen deprivation therapy were included in the analysis. P values apply to between-platform comparisons (CT vs. MRI-guided SBRT) of percentage of patients with clinically relevant deterioration at the specified time point. Clinically relevant deterioration is defined as change from baseline in domain score greater than minimally clinically important difference (MCID), which is 5, 6, 4 and 10 points for panels A, B, C and D, respectively. ADT, androgen deprivation therapy; SBRT, stereotactic radiation therapy.

**Organizing Study Center:**

UCLA Jonsson Comprehensive Cancer Center
Department of Radiation Oncology
200 UCLA Medical Plaza, Suite B-265
Los Angeles, CA 90095
(310) 825-9775

**Principal Investigator:**

Amar U. Kishan, MD
Associate Professor
Department of Radiation Oncology
University of California, Los Angeles

**SCIMITAR** (**S**tereota**C**tic **I**ntensity **M**odulated Rad**I**o**T**herapy **A**fter **R**adical Prostatectomy)
PI: Amar U. Kishan, M.D.

**Co-Investigators:**

Minsong Cao, PhD
Professor
Department of Radiation Oncology
University of California, Los Angeles

Holly Wilhalme, MS
Principal Statistician
Division of General Internal Medicine and Health Services Research
University of California, Los Angeles

Karim Chamie, MD
Associate Professor
Department of Urology
University of California, Los Angeles

Christopher Saigal, MD
Professor
Department of Urology
University of California, Los Angeles

Leslie Ballas, MD
Associate Professor
Department of Radiation Oncology
USC/Norris Comprehensive Cancer Center

Michael L. Steinberg, MD
Professor and Chair
Department of Radiation Oncology
University of California, Los Angeles

Mark S. Litwin, MD
Professor and Chair
Department of Urology
University of California, Los Angeles

Robert E. Reiter, MD
Professor
Department of Urology
University of California, Los Angeles

Nicholas G. Nickols, MD
Assistant Professor
Department of Radiation Oncology
University of California, Los Angeles

**Protocol Overview**

| **SBRT prostate bed*** | **SBRT pelvic nodes (optional)*** | **ADT** (**optional)** | **Follow-up Schedule** | **Tests at each Follow-up (**as described in the Study Calendar) |
|---|---|---|---|---|
| 34 Gy in 5 fractions of 6.8 Gy | 25 Gy in 5 fractions of 5 Gy | 6 months | q 3 months year 1<br>q 6 months year 2-5<br>q yearly thereafter | PSA, Testosterone<br>CTCAE toxicity scores<br>EPIC-26 scores |

**Study Design**: Prospective interventional Study
**Sample size**: 100 patients
**Outcomes**: 4-year biochemical recurrence-free survival, acute and late physician-scored toxicity, patient-reported quality of life outcomes
**Treatment**: SBRT

Prostate Bed PTV (with optional inclusion of pelvic nodes, and optional boost to gross nodules)

**Target Prescription Doses:***

| | |
|---|---|
| Prostate Bed PTV ($PTV_{PB}$): | 34 Gy in 5 fractions, 6.8 Gy per fraction** |
| Pelvic Nodal PTV ($PTV_N$): | 25 Gy in 5 fractions, 5 Gy per fraction (optional elective nodal irradiation) |
| Boost PTV ($PTV_{boost}$): | 40 Gy in 5 fractions, 8 Gy per fraction (optional boost to gross disease, if present) |

* Treatments preferably delivered every other day or may be given consecutive days, with treatment within a period of time may not have exceeded 14 days.

**depending on patient anatomy and ability to meet pre-specific dose constraints, dose range between 30.0-34.0 Gy in 5 fractions will be allowed pending discussion with the study principal investigators

**RT Technique**: image-guided SBRT via IMRT or VMAT, with daily and intra-fraction image-guidance with CBCT on LINAC or on-board MRI on ViewRay

**Androgen Deprivation Therapy**: If performed, collect data up to 6 months duration (which can include neo-adjuvant hormone therapy) consisting of total androgen deprivation (LHRH or GnRH agonist or antagonist + oral anti-androgen)

**SCIMITAR** (**S**tereota**C**tic **I**ntensity **M**odulated Rad**I**o**T**herapy **A**fter **R**adical Prostatectomy)
PI: Amar U. Kishan, M.D.

**INDEX**

**PROTOCOL SYNOPSIS**

**SCHEMA**

**1.0 OBJECTIVES AND ENDPOINTS**

**2.0 BACKGROUND**

**3.0 PATIENT SELECTION**

**4.0 REGISTRATION PROCESS**

**5.0 TREATMENT PLAN**

**6.0 ANDROGEN DEPRIVATION THERAPY**

**7.0 PATIENT ASSESSMENTS AFTER TREATMENT**

**8.0 STUDY CALENDAR**

**9.0 DATA REPORTING AND REGULATORY CONSIDERATIONS**

**10.0 STATISTICAL CONSIDERATIONS**

**11.0 COST CONSIDERATIONS**

**12.0 REFERENCES**

**APPENDIX:** **Informed Consent**
**EPIC /IPSS short form**
**8.0 Study calendar**

## SYNOPSIS

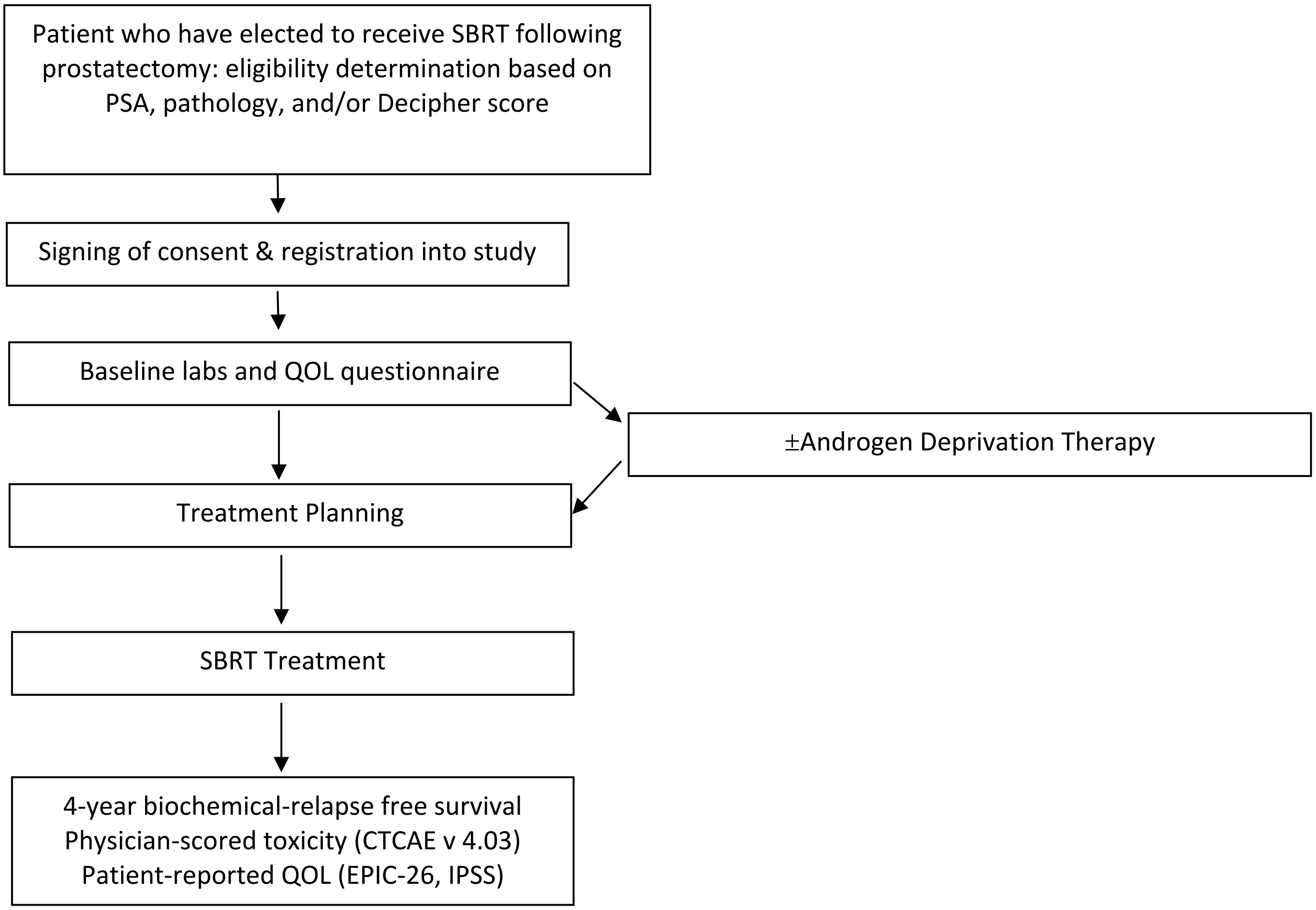
Patient who have elected to receive SBRT following prostatectomy: eligibility determination based on PSA, pathology, and/or Decipher score
Signing of consent & registration into study
Baseline labs and QOL questionnaire
±Androgen Deprivation Therapy
Treatment Planning
SBRT Treatment
4-year biochemical-relapse free survival
Physician-scored toxicity (CTCAE v 4.03)
Patient-reported QOL (EPIC-26, IPSS)

# CLINICAL STUDY SCHEMA

## STUDY DESIGN

Single-arm prospective interventional study assessing the 4-year biochemical recurrence-free survival following adjuvant or salvage SBRT. Physician-scored toxicity and patient-reported quality of life will also be evaluated.

## NUMBER OF PATIENTS

We plan to enroll 100 patients to assess the efficacy of standard SBRT regimen. The rate of accrual is expected to be in the range of 20 patients per year.

## INTERVENTION AND MODE OF DELIVERY

Standard of care image-guided SBRT, either LINAC-based (IMRT or VMAT) or MRI-based (View Ray platform).

## DURATION OF INTERVENTION AND EVALUATION

SBRT, 5 fractions, with androgen deprivation therapy of 6 months duration (optional), follow-up of 4 years.

## STATISTICAL METHODS/SAMPLE SIZE JUSTIFICATION

1.) The study is designed to test the hypothesis that a stereotactic body radiotherapy (SBRT) regimen delivers a higher biologically effective dose fractionated radiotherapy. We project that, on the basis of existing clinical data regarding the incremental benefit of dose-escalation, the SBRT regimen would afford a statistically significant increase in the 4-year biochemical recurrence-free survival from 56% to 72%, corresponding to a hazard ratio of 1.77[1,2]. As detailed in section 11.2 of the protocol, an overall sample size of 60 patients will provide 84.7% power to detect a hazard ratio of 1.77 with a one-sided, one-sample log rank test, at a 0.05 significance level. Given the same estimated HR of 1.77, the expanded sample size of 100 patients will provide a power of 97.0% with a one-sided, one-sample logrank test at a 0.05 significance level. If we stipulated that the historical 4-year BCRFS might have increased to 60% (versus 56%), the estimated HR would be 1.56, and we would have 83.9% power to detect this hazard ratio with a one-sided, one-sample logrank test at a 0.05 significance level provided a sample size of n=100 patients.

2.) The study will also quantify the rate of early and late genitourinary (GU) or gastrointestinal toxicity and compare with historical control rates for post-operative radiotherapy using conventional fractionation. The reference toxicity rate will be an acute grade ≥2 GU toxicity rate (Common Terminology Criteria for Adverse Events version 4.03) of 20%, which is based off the toxicity rate in the 70 Gy arm of the recently published SAKK 09/10 trial[3]. With the assumed true toxicity rate of 20%, a two-sided 95% confidence interval has a margin of error of +/- 11%, which is narrow enough to provide sufficient precision for the estimate of toxicity rate. With a sample size of 100, the two-sided 95% confidence interval has a margin of error of +/- 8.5%.

**FUNDING, REGULATORY, AND FEASIBILITY ISSUES**

The treating department will have the capability, equipment, and expertise to perform image-guided SBRT. Androgen deprivation, clinical evaluation and labs, and radiographic follow-up when necessary will be performed according to standard of care.

Viewray Systems, Inc., will provide additional funding for this trial.

**PATIENT ACCEPTABILTY/ETHICS AND CONSENT ISSUES**

Only patients able to give informed consent will be eligible for the study.

## 1.0 OBJECTIVES AND ENDPOINTS

**PRIMARY OBJECTIVE(S):**

1. To determine the efficacy of postoperative SBRT at a dose of 30-34 Gy in five fractions, as compared with historical control efficacy rates in patients who received fractionated postoperative radiotherapy

2. To observe any toxicity of postoperative SBRT, both via physician-scored and patient-reported metrics.

**SECONDARY OBJECTIVE(S):**

1. To observe the proportion of SBRT fractions for which on-line adaptive radiotherapy is required due to changes in organ-at-risk anatomy, in the subset of patients treated with MRI-guided radiotherapy.

2. To gather biomarkers that may elucidate predictors of increased efficacy or increased toxicity.

**EXPLORATORY OBJECTIVE(S):**

1. To compare toxicity profiles (both physician-scored and patient-reported) between patients treated utilizing a standard linear accelerator versus an MRI-guided linear accelerator platform.

**PRIMARY ENDPOINT(S):**

1. Efficacy of SBRT in the postoperative setting, defined as four-year biochemical recurrence-free survival (BCRFS). Biochemical recurrence (BCR) is defined as serum PSA rising from the post-treatment nadir to a level of 0.2 ng/mL or more with a confirmatory second test, initiation of salvage androgen deprivation therapy, or continued rise in PSA after SBRT.

2. Physician-scored toxicity, represented by the rates of acute (early, within 90 days of SBRT) and late (90 or more days after SBRT) genitourinary and gastrointestinal toxicity based on the Common Terminology Criteria for Adverse Events version 4.03 (CTCAE v 4.03) criteria. These will be analyzed at the following time points with respect to SBRT delivery: 3 months (acute), two years, and four years.

3. Patient-reported toxicity outcomes, as represented by changes in the urinary incontinence, urinary obstruction, bowel, sexual function, and hormone/vitality domains on the Expanded Prostate Cancer Index-26 (EPIC-26) quality of life instrument. International Prostate Symptom Scores (IPSS) will also be obtained as measures of patient-reported toxicity. These will be analyzed at the following time points with respect to SBRT delivery: 3 months (acute), two years, and four years.

**SECONDARY ENDPOINT(S):**

1. The proportion of SBRT fractions for which on-line adaptive radiotherapy was utilized in the subset of patient treated with MRI-guided radiotherapy.

## 2.0 BACKGROUND

### 2.1 The Rationale for Postoperative Radiotherapy

Consideration of postoperative radiotherapy (RT) following radical prostatectomy (RP) for prostate cancer (PCa) is recommended by the American Urological Association, American Society for Radiation Oncology, and National Comprehensive Cancer Center in the setting of adverse pathologic features (APF; typically pT3 disease and/or positive margins) or biochemical recurrence (BCR)[4,5]. The highest level evidence in support of these recommendations are the results of three randomized trials investigating adjuvant radiotherapy (ART) vs.

observation for patients with APFs, which uniformly demonstrated improved BCR-free survival (BCRFS) with ART; one of the trials reported an overall survival benefit as well[6-8]. Notably, the trials did not uniformly require rigorous postoperative PSA analysis to identify patients who might have already experienced a BCR at randomization, and salvage therapy was not standardized. In fact, significant data suggest that salvage radiotherapy (SRT) following BCR is effective, particularly if delivered at lower PSA thresholds[1,9,10], and the optimal timing of postoperative RT remains controversial[11,12]. Three highly anticipated trials—RADICALS-RT, GETUG-17, and RAVES—are currently randomizing patients between ART and SRT. Despite the effectiveness of both ART and SRT, however, postoperative RT in general is underutilized. Indeed, two recently published National Cancer Database (NCDB) analyses demonstrate that only 10-20% of patients with APF receive postoperative radiotherapy (RT)[13,14]. The lack of utilization may be related to provider biases, challenges in engaging patients, and concerns about the balance between efficacy and toxicity[15,16]. Regarding the latter point, updated results of a large, multi-institutional cohort of 2,460 patients undergoing SRT found an overall 5-year BCRFS rate of only 56%, suggesting a need for improved efficacy[17]. While some of this improvement may come from optimized patient selection (e.g., treating patients at lower PSA levels[17,18]), emerging data suggests that dose-escalation may improve BCRFS as well[2]. Recently, the use of the Decipher genomic classifier has been supported by the National Comprehensive Cancer Network to guide post-operative treatment decisions. Specifically, it has been shown that In the setting of localized prostate cancer, the risk groups elucidated by this score have been shown to more accurately predict clinically meaningful endpoints such as distant metastases when compared to traditional risk stratification frameworks that predict the less meaningful outcome of biochemical recurrence.[19] In the post-prostatectomy setting specifically, Decipher has been shown to independently improve prognostication of patients.[20] Moreover, in patients with high Decipher scores, adjuvant rather than salvage RT has been recommended, owing to the higher cumulative incidence of metastases in high-risk patients who wait for their PSA to become detectable.[21]

### 2.2 The Case for Dose-Escalation in Postoperative Radiotherapy

Dose-escalated RT in the definitive setting has been shown to offer a biochemical relapse-free survival (BCRFS) benefit in multiple randomized trials[22-25], and a recent meta-analysis has demonstrated a survival benefit to dose-escalation[26]. Level 1 evidence is not available to support does-escalation in the postoperative setting, but a compelling hypothesis for the merit of dose-escalation exists, as does level 2a evidence[2]. Indeed, a secondary analysis of the randomized SWOG 8794 trial evaluating ART vs. observation found that the predominant pattern of failure in the observation cohort was local rather than distant (24% vs 16%), and the authors suggested that dose-escalation may help further increase local control[27]. The 5-year progression rate of 50% after SRT reported in a large multi-institutional series similarly suggests an insufficient dose[1]. King conducted a meta-analysis of 71 studies including 10,034 patients and identified that the dose of SRT was a statistically significant independent predictor of BCRFS[2]. The dose-response curve was well-modeled by a sigmoidal curve with a projected dose to control 50% of tumors ($TCD_{50}$) of 65.8 Gy; a 2.0% improvement in BCRFS was projected for each single Gy increase in dose. Interestingly, the dose-response curve derived from the results of multiple randomized trials for dose-escalation

in the definitive setting was found to yield a $TCD_{50}$ of 65.9 Gy and a 2.6% per Gy slope—remarkably similar to the curve for SRT. Additionally, multi-institutional study of 2,460 patients treated with SRT across 10 institutions found that an SRT dose of >66 Gy was significantly associated with improved recurrence-free survival[17]. Studying a smaller subset of 1108 patients from this larger cohort who were at higher risk for local recurrence vs. distant recurrence (margin-positive, pre-SRT PSA≤2 ng/mL), Pisansky *et al.* reached a similar conclusion about the association between doses >66.0 Gy and recurrence-free survival. Taken together, these data suggest that a dose equivalent to at least 70 Gy in 2 Gy fractions should be considered to optimize local control in the SRT setting.

The primary concern with dose-escalation, particularly in the postoperative setting, is increased toxicity. Randomized data in this regard are minimal. The ongoing SAKK 09/10 trial randomized patients with BCR after RP (defined as two consecutive increases in PSA with final PSA >0.1 ng/mL, or three consecutive increases) and PSA ≤ 2 ng/mL to 64 Gy vs. 70 Gy in 2 Gy fractions[3]. Toxicity endpoints included acute and late gastrointestinal (GI) and genitourinary (GU) toxicity per the National Cancer Institute Common Terminology Criteria for Adverse Events version 4.0 (CTCAE v4.0). Overall, the rates of acute grade 1, 2, and 3 GU toxicities were 49.7%, 13.0%, and 0.6% in the 64 Gy arm, and 48.6%, 16.6%, and 1.7% in the 70 Gy arm, respectively (with no significant difference between arms). The rate of acute grade 1, 2, and 3 GI toxicities were 36.7%, 16.0%, and 0.6% in the 64 Gy arm, and 49.1%, 15.4%, and 2.3% in the 70 Gy arm, respectively (with no significant difference between arms). Data regarding late toxicity from the SAKK 09/10 trial is not yet available. However, a prospective series from Ghent Hospital described favorable toxicity results among 135 patients receiving 75 Gy in 37 fractions[28]. All patients had pre-SRT PSAs of 0.2 ng/mL or more. The authors used an in-house toxicity scoring system and reported a 28% rate of acute grade 2 GU toxicity and 3% rate of grade 3 GU toxicity. No acute grade 3 GI toxicity was seen, while grade 2 GI toxicity was seen in 15% of patients. For late toxicity, 31% had late grade 2 GU toxicity and 6% had late grade 3 GU toxicity (all strictures); 13% had late grade 2 GI toxicity and 3% had late grade 3 GI toxicity (rectal bleeding and anal pain). Overall, these data imply that dose-escalated postoperative RT is likely to be well-tolerated.

### 2.3 Radiobiological Basis for Hypofractionated Radiotherapy for Prostate Cancer

In contrast to most other solid malignancies, PCa is considered to be relatively more sensitive to large doses of radiation per fraction of RT. This can be numerically quantified by the "α/β" ratio, which is a term that can describe the shape of the survival curve generated by the linear quadratic model of radiobiology and be used to quantify how sensitive a cell is to dose per fraction (the larger β is relative to α, the smaller the α/β ratio is, and the more a given cell type is sensitive to dose per faction).[29] In a landmark study, Brenner & Hall first quantified the α/β ratio for PCa based on clinical outcomes and found it to be 1.5[30]. This value stands in contrast to the much higher values of 8-12 generally used to describe the radiation response properties of most solid tumors and rapidly dividing normal tissues, and is even lower than the values typically used to describe late-responding tissues (α/β~3-5).The underlying biology of why PCa responds differently to large dose fraction size is not yet fully understood, but is likely related to its unique biology, such as very long doubling times and low proportion of actively cycling cells.[30]

Numerous studies have since confirmed that PCa does indeed possess a low α/β, for example a systematic review and analysis combining the clinical outcomes after various hypofractionated RT schedules for PCa over 2,800 patients compared to conventionally fractionated regimens among over 11,000 patients confirmed that prostate cancer has a low α/β of 1.0 to 1.7[31,32] and a separate pooled analysis of 5,969 patients yielded α/β of 1.4Gy.[33] These radiobiological discussions are not merely hypothetical—indeed, emerging clinical evidence from randomized clinical trials of modestly hypofractionated RT versus conventionally fractionated RT suggest similar efficacy with hypofractionation.[34-39] In the most recently reported study, Catton *et al*. found the 5-year biochemical/clinical recurrence disease-free survival rate to be 85% among patients with intermediate-risk PCa who were randomized to receive either 78 Gy in 2 Gy fractions or 60 Gy in 3 Gy fractions[39]. The investigators

also found no differences in the rates of Radiation Oncology Therapy Group (RTOG) late grade ≥ 3 GU and GI toxicity.

Thus, assuming the α/β of PCa is truly in the range of 1.5-2 Gy, as heavily implied by isoeffective tumor control in the aforementioned randomized trials, the therapeutic window of radiotherapy for PCa might be increased by pursuing effective dose-escalation with a regimen than remains isotoxic to a conventional regimen, or toxicity might be lowered with a regimen that remains isoeffective. As described above, the efficacy of SRT leaves room for significant improvement, and therefore hypofractionated postoperative RT constitutes a logical means of achieving dose-escalation without significantly worsening toxicity.

## 2.4 The Role of Hypofractionation in Postoperative Radiotherapy

Several investigators have studied hypofractionated RT in the context of SRT (see Table 1 for a comprehensive review). In an early experience, Kruser *et al*. reported a favorable toxicity profile in a cohort of 108 consecutive patients treated to 65 Gy in 2.5 Gy fractions at a single institution[40,41]. Acute RTOG grade 2 GU toxicity was noted in 5.5% of patients, and acute grade 3 GU toxicity in 1%. Late grade 2 GU toxicities were seen in 15% of patients, with only 3% having persistent late grade 2 toxicity at last follow-up; no grade 3 toxicity was seen. GI toxicity was scored on an institutional scoring system, and the rate of acute grade 2 GI toxicity was 14%. Late grade 2 GI toxicity was seen in 4% of patients, and only one patient had grade 2 toxicity at last follow-up. No acute or late grade 3 GI toxicities were documented. A phase II Italian study of hypofractionated ART and SRT delivered via a simultaneous-integrated boost technique, with 62.5 Gy delivered to the prostate bed in 25 fractions, along with 45 Gy to the pelvic nodes in 25 fractions, also showed favorable acute toxicity results[42].

Two studies in particular have reported higher-than-anticipated toxicity[43,44]. In the largest such study, Cozzarini *et al*. reported the toxicity results of a phase I/II study of hypofractionated postoperative RT, in comparison to a large historical cohort of patients receiving conventionally fractionated SRT to 70.2 Gy in 1.8 Gy fractions[43]. Hypofractionated regimens included 65.8 Gy in 28 fractions (2.35 Gy/fraction, delivered as ART), 71.4-72.8 Gy in 28 fractions (2.6 Gy/fraction), and 58 Gy in 20 fractions (2.9 Gy/fraction). Patients receiving hypofractionated radiotherapy were treated with intensity modulated RT technique. Overall, the 5-year rate of late CTCAE grade 3 GU toxicity was 6.9% in patients receiving conventionally fractionated RT, versus 18.1% in those receiving hypofractionated RT. When the hypofractionated group was subdivided, the 5-year risk of late grade 3 GU toxicity was approximately 17% with doses of 65.8 Gy in 28 fractions and 58 Gy in 20 fractions, and was 20% for 71.4-72.8 Gy in 28 fractions. The risk of grade 3 GU toxicity increased significantly as fraction size increased from 1.80 Gy to 2.6 Gy. A smaller study from Duke University also demonstrated high rates of toxicity[44]. Lewis *et al*. reported a four-year actuarial rate of gross hematuria of 28% among 56 men who received 57.5-65 Gy in 2.5 Gy fractions. Acute CTCAE grade 1 and 2 GU toxicity rates were 43% and 4%, with acute grade 1 and 2 GI toxicity rates of 16% and 4%. No acute grade 3 toxicities were seen. Late grade 2 GU toxicity was seen in 39% of patients, and grade 3 in 27% (all hematuria). In contrast, the rate of grade 3 or higher GI toxicity was 3.6%. Notably, most toxicity resolved with further follow-up.

Despite these two studies, the preponderance of evidence suggests a favorable toxicity profile with hypofractionated RT in the postoperative setting (Table 1). Some have contended that the higher-than-anticipated toxicity stems from an atypical patient population (particularly for the study from Lews *et al*., as other investigators utilized the same regimen without high rates of toxicity) and/or high biologically effective doses with respect to late effects in normal tissues (particularly for the experience reported by Cozzarini *et al*.). Therefore, multiple efforts to investigate hypofractionated postoperative RT remain underway.

Table 1. Comprehensive Review of Toxicity Following Hypofractionated Postoperative Radiotherapy

| Citation | Dose Regimen | Number of Patients | Toxicity Grading Scale | Acute Toxicity | Late Toxicity |
|---|---|---|---|---|---|
| [45] | 50-55 Gy in 2-3.1 Gy fx | 203 | RTOG | | GU<br>Grade 2: 24.7-26.9%*<br>Grade 3: 13.4-15.4%<br><br>GI<br>Grade 2: 3.85-10.1%<br>Grade 3: 0% |
| [46] | 50-52.5 Gy in 2.5 Gy fx | 61 | RTOG | GU<br>Grade 3: 0<br><br>GI<br>Grade 3:0 | GU<br>Grade 2:0<br>Grade 3:0<br><br>GI<br>Grade 2:2.86%<br>Grade 3:0 |
| [40,41] | 65 Gy in 2.5 Gy fx | 108 | RTOG** | GU<br>Grade 2:6.48%<br>Grade 3: 0.93%<br><br>GI<br>Grade 2: 14%<br>Grade 3:0 | GU<br>Grade 2:15%<br>Grade 3:0<br><br>GI<br>Grade 2:4%<br>Grade 3:0 |
| [42] | 65 Gy in 2.5 Gy fx | 49 | RTOG | GU<br>Grade 2:32.6%<br>Grade 3: 0<br><br>GI<br>Grade 2: 9.6%<br>Grade 3:0 | |
| [47] | 54 Gy in 3 Gy fx | 40 | CTCAE | GU<br>Grade 2: 0<br>Grade 3: 0<br><br>GI<br>Grade 2: 17.9%<br>Grade 3:0 | |
| [38], | 65.8 Gy in 2.35 Gy fx<br>71.4–72.8 Gy in 2.55 fx<br>58 Gy in 2.9 Gy fx | 117<br>80<br>50 | CTCAE | | GU<br>Grade 3: 18.1%*** |
| [44] | 65 Gy in 2.5 Gy fx | 56 | CTCAE | GU<br>Grade 2: 4%<br>Grade 3: 0 | GU<br>Grade 2: 39%<br>Grade 3: 27% |

| | | | | | |
|---|---|---|---|---|---|
| | | | | GI<br>Grade 2: 4%<br>Grade 3:0 | GI<br>Grade 2: 8.92%<br>Grade 3:3.57% |
| 48 | 65.5-66 Gy in 2.18-36 Gy fx<br>67.5-71.4 Gy in 2.25-2.55 Gy fx | 125 | CTCAE | GU<br>Grade 2: 12.8%<br>Grade 3: 0.8%<br><br>GI<br>Grade 2: 8.8%<br>Grade 3:0 | |

*Includes patients treated with both adjuvant and salvage radiotherapy
**Late GI toxicity was scored based on the Fox Chase scoring system
***5-year rate of actuarial grade 3 genitourinary toxicity, rather than crude incidence

**2.5 The Role of Stereotactic Body Radiotherapy in the Definitive Treatment of Prostate Cancer**

As discussed above, considerable evidence suggests that the α/β ratio of PCa is between 1.5-2 Gy. In this context, "extremely hypofractionated radiotherapy", wherein a maximum of five fractions of large dose per fraction are delivered, may allow for extreme dose escalation. This constitutes the rationale for utilizing stereotactic body radiotherapy (SBRT) in the treatment of PCa[49,50]. For example, a typical SBRT regimen for PCA—40 Gy in five 8 Gy fractions—would deliver an equivalent dose in 2 Gy fractions ($EQD_2$) of 100-108 Gy to the tumor (α/β of 1.5-2); the corresponding doses to normal tissues would be 74-88 Gy for late effects (α/β of 3-5) and 60 Gy for the acute effects (α/β of 10). The efficacy and tolerability of SBRT regimens in the treatment of low- and intermediate-risk PCa has been well-established. For instance, King *et al.* recently reported outcomes for 1100 patients enrolled in separate prospective clinical trials.[51] Fifty-eight percent of patients had low-risk disease, 30% had intermediate-risk disease, and 11% had high-risk disease. With a median follow-up of 36 months, the 4-year BCRFS was 95%, 84%, and 81% for low-, intermediate-, and high-risk patients (93% overall). Among the 193 patients with a minimum follow-up of 4 years, the 4-year BCRFS was 99% and 93% for those with low- and intermediate-risk PCa, respectively.

SBRT regimens are tolerable as well, with low rates of serious toxicity. In a separate report, King *et al.* reported on Expanded Prostate Cancer Index Composite-26 (EPIC-26) quality of life (QOL) changes in 864 patients enrolled on various prospective SBRT trials who had data available at baseline and regular follow-up (median of 3 years)[52]. EPIC-26 declines in urinary, bowel, and sexual domains were minimal. A recent synthetic review of the literature compiled outcomes of 1812 patients treated with SBRT and found that the incidence of acute grade ≥3 GU and GI toxicities (by RTOG or CTCAE scales) were 0.28% and 0.17%, respectively; corresponding rates of late grade ≥3 GU and GI toxicities were 1.61% and 0.61%, respectively[50].

As a result of these promising results, the ASTRO model policy update in 2013 recognized SBRT as an alternative to conventionally fractionated radiotherapy for PCa, noting "SBRT could be considered an appropriate alternative for select patients with low to intermediate risk disease."[53] Since 2014, the National Comprehensive Cancer Network guidelines have stated that "extremely hypofractionated image-guided IMRT/SBRT regimens (6.5 Gy per fraction or greater) are an emerging treatment modality with single institutional and pooled reports of similar efficacy and toxicity to conventionally fractionated regimens. They can be considered as a cautious alternative to conventionally fractionated regimens at clinics with appropriate technology, physics, and clinical expertise [for low- and intermediate-risk PCa]."[5]

Data emerging in 2019 provide considerable evidence that SBRT should be an acceptable standard option for patients with low- and intermediate-risk disease. These include the long-term results of the randomized HYPO-RT-PC trial,[55] results from a pooled consortium of phase II studies,[56] and, most recently, the acute toxicity

results from the randomized PACE-B trial.[57] As of 2019, the NCCN guidelines do endorse SBRT as a standard of care option for patients with low- and favorable intermediate-risk disease, and note that SBRT can be performed for all other patients in the event of logistical difficulties or patient preferences.

In addition to the aforementioned theoretical radiobiological advantages to SBRT for PCa, economic considerations are also worthy of discussion, given the increasing incidence of PCa and the projected $18.53 billion annual cost of PCa care in 2020.[54,55] A recently published time-driven activity-based costing analysis of various treatment modalities for low-risk PCa estimated the average cost of a course of IMRT to be $23,565, versus $8,978 for low-dose rate brachytherapy and $11,665 for SBRT.[56] A Markov decision model also found that the average cost of SBRT was substantially lower than IMRT ($35,431 vs. $22,152), as did a Medicare claims analysis ($13,645 vs. $21,023).[57,58]

Finally, the role of SBRT in high-risk PCa is an emerging area of clinical investigation. The early toxicity results of a multi-institutional trial based out of the University of California, Los Angeles (https://clinicaltrials.gov/ct2/show/NCT02296229) are encouraging[59]. Among 61 patients with a median followup of 12 months, Kishan *et al*. reported rates of acute and late grade 2 CTCAE GU toxicity of 13.1% and 6.7%, respectively. Rates of acute and late grade 2 GI toxicity were 6.6% and 8.2%. No grade 3 acute or late toxicities were seen. Importantly, toxicity was no different in patients receiving ADT (64.4% of patients) or nodal irradiation at a dose of 25 Gy in 5 fractions (37.1% of patients).

The role of SBRT in the postoperative setting is, by comparison, not well studied. Indeed, only one prior report of SBRT in this setting exists, and only in abstract form. Sampath *et al*. reported preliminary results of 14 patients treated with escalating doses of post-prostatectomy SBRT, with three receiving 7 Gy x 5, seven receiving 7.5 Gy x 5, and four receiving 8 Gy x 5[60]. Median follow-up durations were 20 months, 7 months, and 3 months for the different cohorts. Acute CTCAE grade 2 GI events were seen in 5 patients (35.7%), and four patients (28.6%) had acute grade 1 urinary toxicity. No grade 3 or higher acute toxicity was seen, and no patients experienced grade 2 or higher late toxicity.

Given the purported low $\alpha/\beta$ ratio of PCa, the frequency of local failure even after postoperative RT, and the known radiation dose-response curve in the post-prostatectomy setting, a five-fraction SBRT offers the potential of improving the therapeutic window of ART or SRT, while simultaneously providing improved patient convenience and lowering costs to the healthcare system.

Since the initiation of our trial, two phase I reports of SBRT after radical prostatectomy have been published. In the first trial, 12 patients were treated with a dose of 7 Gy x 5.[45] There were no grade 3 or greater acute GU or GI toxicity events by the CTCAE scale. No efficacy or long-term toxicity data were available. In the second trial, 26 patients received 7-9 Gy x 5, with a median follow-up of 60 months for 35 Gy, 48 months for 40 Gy, and 33 months for 45 Gy[64]. Late grade ≥2 and ≥3 GI toxicity occurred in 11% and 0% of patients, respectively, and late grade ≥2 and ≥3 GU toxicity occurred in 38% and 15% of patients, respectively. The crude rate of BCR was 58%, but no ADT or nodal therapy was allowed. Overall, both studies strengthen the precedence for further prospective study of SBRT in the post-prostatectomy setting.

## 2.6 Post-Operative SBRT

On the basis of the efficacy and safety of SBRT in the definitive setting, as well as the rationale for hypofractionation in the postoperative setting, it is reasonable to explore the role of SBRT in the postoperative setting. A regimen of 34 Gy in 5 fractions of 6.8 Gy would yield an effective tumor $EQD_2$ of 74.8-80.63 Gy ($\alpha/\beta$ of 1.5-2); corresponding doses to normal tissues would be 57.31-66.4 Gy for late effects ($\alpha/\beta$ of 3-5) and 47.6 Gy for acute effects ($\alpha/\beta$ of 10). The effective doses to normal tissue constitute a 23-25% reduction for late effects and a 21% reduction for acute effects beyond the SBRT regimen already shown to be safe and effective in the treatment of definitive PCa. Notably, the classical dose-tolerate limit for whole organ radiation is 65 Gy for the bladder and 60 Gy for the rectum[61], and more recently refined partial-volume radiation tolerances suggest that

up to 50% of the bladder can receive 65 Gy, and up to 35% of the rectum can receive 60 Gy[62]. With the conformality achieved by the SBRT planning process, it is unlikely that these tolerances will be exceeded at the doses being prescribed. The highest toxicity rates in the previously discussed hypofractionated postoperative RT studies were seen in patients receiving 71.4-72.8 Gy in 28 fractions, and 57.5-65 Gy in 2.5 Gy fractions[43,44]. The $EQD_2$ of these regimens for late effects on normal tissue are up to 79.04-81.54 Gy and 69.64-71.5 Gy. Notably, another study used the same regimen of 65 Gy in 25 Gy fractions and found very favorable late GU toxicity profiles[40]; this could reflect lower median follow-up, or could simply suggest that the risk of late toxicity with this regimen is still uncertain. Nonetheless, the effective dose for late effects would be substantially lower with the suggested SBRT regimen than with either hypofractionated regimen associated with high toxicity.

With regards to clinical efficacy, the $EQD_2$ to PCa cells delivered by 34 Gy in 5 fractions, with a conservative $\alpha/\beta$ estimate of 2 for PCa cells, would be 74.8 Gy. Following the sigmoidal dose-response curve for postoperative RT, this would offer a 16% increase in 5-year BCRFS over the 56% 5-year BCRFS rate identified in a large cohort of patients treated with SRT to a median dose of 66 Gy[2,17].

## 2.7 The Role of Androgen Deprivation Therapy (ADT) with Postoperative Radiotherapy

A survival benefit to upfront androgen deprivation therapy (ADT) with definitive RT in the setting of clinically localized PCa has been validated in multiple randomized controlled trials[63-67]. The role of concomitant ADT with postoperative RT is less well-defined. Several retrospective studies have previously suggested a benefit. In a cohort of 122 patients receiving 64.2-67 Gy of SRT, King *et al*. identified a 5-year BCRFS survival benefit for the subset of 53 patients who received a 4-month course of combined androgen blockade (57% vs. 31%)[68]. Similarly, Ost *et al*. found that the addition of concomitant ADT (median duration of 6 months) was associated with an improved BCRFS in a cohort of 225 patients receiving 69.2 Gy of ART (HR 0.04)[69]. More recently, two high-profile randomized clinical trials have also suggested a benefit to concomitant ADT. The GETUG-16 trial enrolled 743 men with PSA >0.2 and ≤2.0 ng/mL who initially had a PSA <0.1 for at least six months after RP, and randomized them to receive SRT to 66 Gy or the same SRT with 6 months of goserelin[70]. Patients receiving ADT along with SRT had a significantly higher freedom from biochemical or clinical progression at 5 years when compared with those who received SRT alone (rates of 80% vs. 62%). Notably, the interquartile range of PSAs among patients on the study was 0.2-0.5 ng/mL, suggesting that most received what would be classified as early SRT. A more clinically relevant benefit to ADT with SRT was recently demonstrated by the recently published results of the RTOG 9601 study[71]. The investigators randomized 760 men with pT2 PCa with positive surgical margins or pT3 PCa who had PSA levels between 0.2-4.0 ng/mL to receive 64.8 Gy with or without 24 months of bicalutamide. With a median follow-up of 13 years, a significant overall survival benefit to ADT was identified (12-year actuarial overall survival of 76.3% vs. 71.3%). The interquartile range of PSAs at time of SRT was 0.4-1.1 ng/mL and the median PSA was 0.6 ng/mL, suggesting that the patients enrolled in RTOG 9601 were at higher baseline risk of clinical events than those enrolled on GETUG-16. In fact, on subgroup analysis, the overall survival benefit was only apparent in patients with PSAs of 1.5-4.0 ng/mL at the time of SRT. The ongoing RADICALS (https://clinicaltrials.gov/ct2/show/NCT00541047) and RTOG 0534 (https://clinicaltrials.gov/ct2/show/NCT00567580) trials may potentially provide further insight into the role of ADT in this setting.

Currently, patients presenting for consideration of postoperative RT should be offered a nuanced discussion of the benefits and risks of ADT, with the understanding that if ADT is indicated, a short, 6 month course is likely to be sufficient in the majority of patients presenting in the modern era with low (<0.5 ng/mL) post-operative PSA. Concomitant ADT should be strongly considered for patients with Gleason score 8 or higher disease, tertiary Gleason pattern 5 disease, evidence of seminal vesicle invasion, and/or PSA doubling times of <6 months.

## 2.8 The Role of Pelvic Nodal Irradiation with Postoperative Radiotherapy

Elective irradiation of pelvic nodal basins remains a controversial topic in the radiotherapeutic management of PCa in the definitive setting, let alone the postoperative setting[72]. Indeed, despite promising retrospective evidence of a benefit to whole pelvis RT (WPRT), the RTOG 9413 and GETUG-1 trials, which randomized patients to WPRT vs RT to the prostate alone, were both negative[73,74]. No prospective evidence is available in the setting of SRT. In a retrospective study, Spiotto *et al*. reported that within a group of 114 patients thought to have high-risk of lymph node relapse, the 72 patients who received WPRT were found to have a significantly higher BCRFS (47% vs. 21%)[75]. The investigators also found that WPRT was not associated with a benefit in patients thought to be of low-risk of pelvis nodal relapse. Moghanaki *et al*. compared outcomes between 112 patients who received WPRT with SRT and 135 who received SRT to the prostate alone; overall, they found no difference in biochemical relapse-free survival[76]. However, in the subset of patients with pre-SRT PSA of >0.4 ng/mL, WPRT was associated with a 53% reduction in the risk of biochemical progression (adjusted HR 0.47). Notably, patients in this study did not receive ADT. Finally, a recent study of 163 patients receiving SRT, of whom 29 received WPRT, found a significantly higher 4-year BCRFS following propensity score-matching for the WPRT cohort (63.1% vs. 43.4%)[77]. This benefit was driven by patients with seminal vesicle invasion, Roach score for lymph node invasion ≥ 45%[78], and number of harvested lymph nodes ≤ 5. A prior, smaller study of 46 patients, also suggested a 10-year BCRFS survival benefit among the 21 patients receiving WPRT, though it did not reach statistical significance[79]. The current RTOG 0534 (https://clinicaltrials.gov/ct2/show/NCT00567580) randomizes patients to receive prostate-only SRT ± ADT or WPRT + ADT, but preliminary results are not available.

Because the preponderance of available data suggests a potential benefit to WPRT in the context of postoperative RT in a subset of patients with higher-risk features, WPRT should be strongly considered for men with high PSAs at the time of SRT (>0.4 ng/mL), Gleason score 8 or higher disease, tertiary Gleason pattern 5 disease, evidence of seminal vesicle invasion, and/or PSA doubling times of <6 months.

## 3.0 PATIENT SELECTION

### 3.1 Eligibility criteria:

3.1.1. History of histologically confirmed, clinical localized adenocarcinoma of the prostate treated with radical prostatectomy with definitive intent

3.1.2. Presence of adverse pathologic features at the time of prostatectomy (positive surgical margin, pathologic T-stage 3-4 disease, pathologic Gleason score 8-10 disease, presence of tertiary Gleason grade 5 disease) **OR** documentation of rising prostate-specific antigen on at least two consecutive draws, with the magnitude of prostate-specific antigen exceeding 0.03 ng/mL **OR** an intermediate- or high-risk Decipher genomic classifier score.

3.1.3. CT scan and MRI of the pelvis within 120 days prior to enrollment [note: (a) if patient has medical contraindication to MRI, an exemption will be granted and enrollment can proceed; (b) for patients with PSA <1.0 ng/mL, the treatment planning CT can substitute for a diagnostic CT scan; (c) a low-field, radiation planning MRI can replace the diagnostic MRI if the patient refuses or cannot obtain a high-field MRI]

3.1.4. Bone scan within 120 days prior to enrollment; if the bone scan is suspicious, a plain x-ray and/or MRI must be obtained to rule out metastasis, and advanced imaging (e.g., $^{18}$NaF PET/CT) is strongly recommended. Advanced imaging studies (i.e. PSMA PET and Axumin scan) can supplant a bone scan if performed first. A bone scan will not be necessary for patients with PSA <1.0 ng/mL **OR** if the patient is being treated on the basis of adverse pathologic features alone (i.e., without a rise in the PSA) **OR** on the basis of high-risk Decipher genomic classifier alone.

3.1.5. Age ≥ 18

3.1.6. KPS ≥ 70 and/or ECOG ≤2

3.1.7. Ability to understand, and willingness to sign, the written informed consent

### 3.2. Non-eligibility criteria:

3.2.1. Patients with any evidence of distant metastases. Note, evidence of lymphadenopathy below the level of the renal arteries can be deemed loco regional per the discretion of the investigator.

3.2.2. Patients with pathologically-confirmed N1 prostate cancer

3.2.3. Patients with neuroendocrine or small cell carcinoma of the prostate

3.2.4. Prior cryosurgery, HIFU or brachytherapy of the prostate

3.2.5. Prior pelvic radiotherapy

3.2.6. History of Crohn's Disease, Ulcerative Colitis, or Ataxia Telangiectasia

# 4.0 REGISTRATION PROCESS

## 4.1. General guidelines

Patients seen as new patients in consultation in the radiation oncology clinic who are being evaluated for potential postoperative radiotherapy for prostate cancer options will be informed of this clinical study if eligible. IRB approved patient flyers, website content, and letters to referring physicians will be used to educate potential patients and referring physicians regarding the study goals and logistics in order to recruit interested patients who have elected to receive SBRT treatment following their prostatectomy. The decision to participate will be entirely voluntary.

## 4.2. Registration Process

Informed consent form will be given to the patient for review. Consent will be obtained after a clear and thorough discussion between the patient and the treating physician. Whenever practical and in the best interest of subjects and staff, the consent (and/or reconsent) discussion may occur remotely via telephone and/or UCLA-approved telemedicine processes. The IRB-approved consent document may be signed by the subject at his home, and returned to UCLA study investigator for counter-signature. To register a patient, the research coordinator will obtain or complete: (1) pathological documentation of adenocarcinoma of the prostate (UCLA pathology review not required); (2) signed informed consent form; (3) signed HIPAA authorization form.

Upon confirmation of eligibility and enrollment in the study, the following will be obtained: (1) Medical history, clinical examination and consultation with Radiation Oncology; (2) Signed informed consent.

Subjects will be asked to provide re-consent based on any updated risks, benefits or other new information which comes to light during the conduct of the trial which may impact their willingness to continue to participate. In instances where subjects are being followed by telephone, re-consent may be conducted by telephone using a prepared, IRB-approved re-consent script.

## 5.0 STUDY PLAN

### 5.1. Radiotherapy Treatment Platform

Enrolled patients are eligible for treatment with either a standard linear accelerator (LINAC) or an MRI-guided linear accelerator (MRIdian System™, ViewRay™, Cleveland, OH, USA). The MRI-guided device is only available for clinical use in the facility located at 200 UCLA Medical Plaza Building, B-265 (Westwood location); LINACs are available at both the Westwood location and at the facility located at 1223 16th Street, Suite 1100 (Santa Monica location). Currently, nearly all patients receiving postoperative RT are treated with LINACs. All patients who are treated at the Westwood location will be considered for treated with the MRI-guided device, unless they refuse due to claustrophobia or have another contraindication to MRI. For patients being considered for treatment with the MRI-guided device, a plan comparison will be made for LINAC-based treatment. Final choice of treatment platform will be made based on physician discretion based on evaluation of the radiation plan. Patients treated at the Santa Monica location will be treated with a LINAC.

### 5.2. Radiotherapy Simulation

Enrolled patients will undergo CT simulation and planning as per routine for post-operative prostate cancer patients. Patients will be given a bladder filling protocol with instructions to void one hour prior to simulation (and prior to each treatment), and drink at least two eight ounce glasses of water. This protocol has been shown to stabilize bladder anatomy during SBRT courses[80]. At the time of simulation, a custom vacloc bag, alpha cradle, or equivalent immobilization device will be used for patient immobilization and establishment of treatment geometry. Alternatively, the patient will be instructed to perform a Fleets enema three hours before simulation. A pelvic CT without contrast will be performed for radiotherapy simulation (i.e., treatment planning CT) with a slice thickness of 1.5 mm. Additionally, an MRI will be obtained in the treatment position on the MRI-guided linear accelerator if the patient is being considered for treatment on this device (i.e., for all patients being treated in Westwood). These procedures are considered standard-of-care for prostate radiotherapy planning.

### 5.3. Radiotherapy Contouring and Planning

The study investigator will define all relevant clinical target volumes (CTVs) on the treatment planning CT, and on the treatment planning MRI if the patient is being considered for treatment on the MRI-guided linear accelerator. If available, a diagnostic pelvic MRI will be fused with the treatment planning CT and will utilized as a supplement for anatomical contour delineation.

The prostate bed CTV ($CTV_{PB}$) will be defined according to the RTOG consensus guidelines[81]:

<u>Superiorly</u>: The $CTV_{PB}$ should extend superiorly from the level of the caudal vas deferens remnant; if this cannot be visualized, it should extend 2 cm above the pubic symphysis. The whole seminal vesicle remnant should be included for patients with pathologic T3b disease.

<u>Inferiorly</u>: The $CTV_{PB}$ should extend inferiorly to >8-12 mm inferior to the vesicourethral anastomosis (VUA). If this is difficult to visualize, the inferior border can be the level just above the penile bulb.

<u>Anteriorly</u>: Below the superior border of the pubic symphysis, the anterior border is at the posterior aspect of the pubis. Above the pubic symphysis, the anterior border should encompass the posterior 1-2 cm of the bladder wall at the minimum.

<u>Posteriorly</u>: Below the superior border of the pubic symphysis, the posterior border is the rectal wall. Above the pubic symphysis, the posterior border is the mesorectal fascia.

<u>Laterally</u>: Laterally, the $CTV_{PB}$ should extend to the levator ani below the level of pubic symphysis, and to the obturator internus above the level of the pubic symphysis.

If WPRT is to be delivered, the obturator, presacral, internal iliac, and external iliac nodal stations will be considered as targets. The nodal CTV ($CTV_N$) will be defined as per the RTOG consensus guidelines[82]. If gross disease is seen on pre-radiation imaging, then it should be contoured as gross tumor volume ($GTV_{boost}$). All volumes ($CTVP_B$, $CTV_N$, and $GTV_{boost}$) will be expanded isotropically by 3-5 mm to form corresponding planning target volumes ($PTV_{pb}$, $PTV_n$, $PTV_{boost}$) Prescription doses for the various PTVs are shown below. For all PTVs, the prescription dose will be prescribed such that 95% of the PTV receives the prescription dose.

Table 2. Target Prescription Doses

| | |
|---|---|
| $PTV_{PB}$* | 34 Gy in 5 fractions, 6.8 Gy per fraction |
| $PTV_N$** | 25 Gy in 5 fractions, 5 Gy per fraction |
| $PTV_{boost}$** | 40 Gy in 5 fractions, 8 Gy per fraction |

*Selective dose range of 30-34 Gy in 5 fractions will be considered appropriately if deemed necessary due to patient anatomy or dosimetric constraint issues related to the dose to organs at risk as outlined below in section 5.4. A dose below 34 Gy in 5 will be determined as clinically appropriate.
**Targeting of these PTVs is optional and at the recommendation of the treating physician.

### 5.4. Organs at Risk (OAR) Dose Constraints

Delineation of normal structures will include the bladder, rectum, rectal wall (anterior and posterior at level of the prostate gland), femoral heads, small bowel, and urethra (optional). The penile bulb, urogenital diaphragm area and the general areas of the posterolateral neurovascular bundles (optional) will be defined and contoured only for dose-tracking. The radiation physicist will optimize the radiation therapy treatment plan and the responsible study investigator will review it prior to approval for treatment. Dose volume histograms (DVH), and normal tissue constraint parameters specified below will be used to judge the quality of the plan and optimize doses to the PTV as well as maximally sparing of OARs.

The dose constraints below for OARs will be used to assess dosimetry. These are adapted from an existing clinical trial examining SBRT in the definitive treatment setting, taking into account a hypothetical increased radiosensitivity of the bladder in the postoperative setting (https://clinicaltrials.gov/ct2/show/NCT02296229). Doses that exceed the constraints below will be considered deviations from the protocol, and can be delivered if study investigators agree that the deviation is acceptable and unlikely to cause excessive morbidity. Doses listed are as total over 5 fractions and per fraction. In order to verify each of these limits, the organs must be contoured such that appropriate dose volume histograms can be generated.

If the pre-specified constraints cannot be met, or if the patient has unique anatomical considerations, the principal investigators will discuss the case and on a case-by-case basis, and a dose as low as 30 Gy in 5 fractions will be deemed clinically appropriate and not considered a protocol violation. If constraints cannot be met with a dose of 30 Gy in 5 fractions, then the patient will be considered ineligible for the study.

Table 3. Organ-At-Risk Dose Constraints

| **Organ-at-Risk** | **Volume** | **Dose (cGy)** |
|---|---|---|
| Anterior rectal wall (half) | Maximum point dose | No more than 105% of the prescription dose |
| Posterior rectal wall (half) | Maximum point dose | No more than 40% of the prescription dose (ideal)* |
| Rectal wall | <50% circumference | 24 Gy |
| Rectum | 45% | 27.5 Gy |
| | 30% | 32.5 Gy |
| | 25% | 33.75 Gy |
| Small intestine | Maximum point dose | 25 Gy (5.0 Gy per fraction) |

| | | |
|---|---|---|
| | Less than 10 cc | 20 Gy (4.0 Gy per fraction) |
| Bladder | Maximum point dose | No more than 105% of prescription dose |
| | 35% | 32.5 Gy |
| Penile bulb | Maximum point dose | For tracking purposes only |
| Femoral heads | Less than 10 cc cumulative (both sides) | 20 Gy (4.0 Gy per fraction) |

*This is only an ideal constraint

**5.5. Radiation Therapy Delivery: Patient set-up / Image Guidance**

Patient immobilization will be the same as at simulation (i.e., a custom vacloc bag, alpha-cradle or equivalent immobilization device should be used). The same bladder filling protocol as employed at simulation will be used again. Image-guidance will be determined on the basis of the treatment platform. For LINAC-based treatments, daily pelvic cone-beam CT must be performed to evaluate bladder and rectal distension. If the maximal rectal diameter differs at the time of SBRT from the maximal rectal diameter at the time of treatment planning CT by >1 cm, then the patient will be encouraged to evacuate the rectum. Three such attempts will be allowed. If anatomy is still unfavorable after three attempts, then SBRT will not be delivered and this will be counted as an aborted treatment. Images must be approved by an attending radiation oncologist before treatment is initiated. For MRI-based treatments, a 175 second free breathing scan will be taken utilizing the on-board MRI to establish target and OAR geometry at the time of treatment. If deemed necessary, on-line adaptive planning will be performed, wherein the relevant GTVs, CTVs, and OARs are adjusted to match current geometry and the radiation plan adjusted accordingly.

For LINAC-based treatment, the standard protocol will be for two full arcs of volumetric modulated arc radiotherapy, with a cone-beam CT image taken after the first full arc to determine if intrafractional motion has occurred. For MRI-based treatments, real-time MRI information will be available. Gating-based treatment, with gating based on rectal distention, can be employed at the discretion of the treating physician. Treatments will be delivered every other day, or consecutive days if necessary with all fractions to be delivered within a period not exceeding 14 consecutive chronologic days. For either LINAC-based or MRI-based treatments,

**5.6. Specimen Collection for Translational Research (Whenever feasible)**

The overall objective of collecting specimens for translational research is to prospectively establish a repository of biospecimens from patients to facilitate current and future hypothesis generated research. Translational research studies integrate the newest research findings into current protocols to investigate important biologic questions. For this protocol, cheek swabs, as well as blood specimens for research will be collected from each patient. Collection of these samples will occur whenever feasible and based on the best clinical decision-making of the investigator. Cheek swabs will be collected once, and blood samples at four time points: Once prior to initiating treatment, once at any time during radiation therapy, and then at approximately the 3 and 6 (Optional) month follow-up visit time points. All blood samples must be immediately refrigerated until pick up. All specimens must be labeled with protocol number, patient registration number, and date of specimen collection.

It will be highly encouraged, but not required, that patients enrolled on this study have their original prostatectomy and prostate biopsy specimens submitted for analysis using the Decipher genomic classifier. The Decipher genomic classifier has recently emerged as a Medicare-approved tissue-based prognostic tool for guiding management following RP.[83] The assay generates a score based off of the RNA expression signature of 22 marker genes related to cell proliferation, differentiation, androgen signaling, motility and immune modulation. Decipher was originally derived from a RP series comparing gene expression profiles of 192 patients with early metastases (i.e., within 4 years) versus 271 controls,[84] and has since been validated in multiple studies.[85-90]

## 6.0 ANDROGEN DEPRIVATION THERAPY

Androgen deprivation therapy (ADT) will be delivered at the discretion of the treating physician. ADT will consist of combined androgen blockade, which is comprised of (a) a luteinizing hormone-releasing hormone agonist (e.g., Lupron) or a gonadotropin-releasing hormone antagonist (e.g., Degarelix) and/or (b) an oral anti-androgen (eg. Casodex) ); use of both (a) and (b) together will be considered combined androgen blockade when the oral anti-androgen is used for a minimum of 2 months. When used, ADT will be limited to a course of 6 months (which can include neo-adjuvant hormone therapy). Concomitant ADT should be strongly considered for patients with Gleason score 8 or higher disease, tertiary Gleason pattern 5 disease, evidence of seminal vesicle invasion, and/or PSA doubling times of <6 months.

## 7.0 PATIENT ASSESSMENTS AFTER TREATMENT

Patients will be followed clinically after treatment per standard of care. The study calendar (section 9 below) includes a summary of the follow-up assessments.

### 7.1. Measures of Oncologic Efficacy

Follow-up for patients in this study will be consistent with patients managed with postoperative RT. This follow-up consists of PSA drawn every 3 months for the first year, then every 6 months until 4 years have passed since SBRT, and then once per year subsequently. Data of routine imaging (bone scan, CT or MRI) as clinically indicated will be collected on any patient who presents with any symptoms or PSA progression consistent with cancer recurrence. Recurrences will be managed according to the standard of care after primary radiotherapy for prostate cancer.

Disease Status Definitions:
After study entry, disease evaluations will be made and recorded using the following criteria:
*7.1.1. No Evidence of Disease (NED):*
No clinical or biochemical (i.e., PSA-based) evidence of disease recurrence
*7.1.2. Biochemical Recurrence:*
PSA rising above pre-SBRT level and/or continued rise in PSA
*7.1.3. Progression of Disease (PD):*
Progression of disease will be declared if one or more of the following criteria are met:
Biochemical recurrence
Initiation of salvage therapy
Clinical or radiographic evidence of metastases
*7.1.4. Local Progression:*
Local progression will be determined by imaging evidence of a recurrence within the prostate fossa, which is proven on biopsy to be recurrent PCa
*7.1.5. Distant Failure:*
Distant failure will be determined by documented nodal or skeletal metastases as evidenced by imaging
*7.1.6. Death from prostate cancer:*
Cause of death will be determined by death certificates
Disease-Free Interval Definitions:
The disease-free intervals will be measured from the date of accession to the date of documentation of progression or until the date of death (from other causes)
*7.1.7. Time to Biochemical Recurrence:*
The date of biochemical recurrence will be defined as the date of a PSA laboratory value that exceeds the pre-SBRT PSA.
*7.1.8. Time to Progression:*
This will be measured from the date of study entry to the date of whichever occurs first from the following events: biochemical failure, initiation of salvage therapy post-SBRT, clinical or radiographic evidence of tumor recurrence.
*7.1.9. Time to Local Progression:*
The time to progression will be measured from the date of study entry to the date of documented local progression as determined by imaging evidence of a recurrence followed by histologic confirmation by biopsy.
*7.1.10. Time to Distant Failure:*

The time to distant failure will be measured from the date of study entry to the date of documented regional nodal recurrence or distant disease relapse. Patients with evidence of biochemical failure, but a negative prostate biopsy, will be considered as distant failure only.

*7.1.11. Disease-Specific Survival:*

Disease-specific survival will be measured from the date of study entry to the date of death due to prostate cancer. The following will be considered as failure events in assessing disease specific survival:

- Death certified as due to prostate cancer.
- Death from other causes with active malignancy (clinical or biochemical progression).
- Death due to complications of treatment, irrespective of the status of malignancy.
- Death from other causes with previously documented relapse (either clinical or biochemical) but inactive at the time of death will not be considered in disease-specific survival, but will be analyzed.

*7.1.12. Overall Survival:*

The survival time will be measured from the date of study entry to the date of death. All patients will be followed for survival and effort will be made to document the cause of death.

**7.2 Toxicity Scoring and Reporting**

The study will not be using separate toxicity scales for acute and late radiation adverse events.

*7.2.1. Physician-scored toxicity*:

All acute and late adverse events from protocol radiation therapy will be reported and scored for severity using the NCI Common Terminology Criteria for Adverse Events (CTCAE) version 4.0.

*7.2.2. Patient-reported toxicity*:

Patient-reported quality of life measures will be collected with the Expanded Prostate Cancer Index-26 (EPIC-26) questionnaire to assess changes in the urinary incontinence, urinary obstruction, bowel, sexual function, and hormone/vitality domains. International Prostate Symptom Score (IPSS) data will also be obtained. Data will be obtained at baseline (pre-treatment), at 1 month post-SBRT, at 3 months post-SBRT, and then every 3 months for the first year after treatment, and then every 6 months for a minimum of 4 years after treatment. After 4 years have been elapsed, these will be collected on an annual basis. Visits after 3 months may be performed remotely.

**7.3. Relevant SBRT-Related Treatment Toxicities**

These are the significant and relevant toxicities that are most likely to be related to therapy.

*7.3.1. Constitutional symptoms:*

Constitutional symptoms that may be attributed to radiation therapy are rare, but include loss of appetite and fatigue. Patients will be seen on a weekly basis while they are receiving radiation therapy per standard of care. Counseling and medications may be prescribed to alleviate these symptoms while the patient is on treatment. It is expected that symptoms will improve and self-resolve 2-4 weeks after completion of therapy.

*7.3.2. Radiation proctitis:*

Radiation proctitis is due to radiation-induced inflammation of the rectum starting roughly 1 week after treatment. Patients will be seen at least on a weekly basis while they are receiving radiation therapy per standard of care. Counseling, diet and medications may be prescribed to alleviate these symptoms while the patient is on treatment. It is expected that that symptoms will improve and self-resolve within 4-8 weeks after completion of therapy.

*7.3.3. Radiation cystitis/urethritis:*

Radiation cystitis/urethritis is due to radiation-induced inflammation of the lower urinary tract (bladder/urethra) starting during or within 1-2 weeks after treatment. Patient will be seen on a weekly basis while they are receiving radiation therapy per standard of care. Counseling and medications may be prescribed to alleviate these symptoms while the patient is on treatment. It is expected that that symptoms will improve and self-resolve 4-8 weeks after completion of therapy.

## 8.0 STUDY CALENDAR

Section Moved to Appendix section - page 30

## 9. DATA REPORTING AND REGULATORY CONSIDERATIONS

### 9.1. Monitoring Plan

All participating sites will be monitored in accordance with the standards required by the UCLA DSMB approval and Data Safety Monitoring Plan. Any potential adverse events will be discussed at a monthly internal study team meeting, led by the principal investigators (AUK and CRK). In addition, adverse events will be presented at the UCLA Radiation Oncology monthly QC meeting.

### 9.2. Data Management

The research staff will be responsible for the database records of study patients. The data will be kept on the research coordinators' computers or the electronic database, under password protection, with the patient information de-identified (study patients will be referred by their coded study number). A chart with all the relevant research patient information will be maintained for each patient by the research coordinator, and will be filed in a firewall protected computer or locked drawers. Subject data may be entered into either paper or electronic case report forms. Only the research team (study coordinator, investigators, and project supporting staff) will have the password and key to the data from the study patients.

### 9.3. Confidentiality

Study data will be maintained in password protected computer files or the electronic database. Only research personnel will have access to this information. All identifiers will be removed. Specimens will be stored under the patient's coded study number. The patient's name or other public identifiers will not be included in any information shared with other investigators. The master key that will identify specific study patients to their coded study number will be kept in a separate password protected file on the research coordinator's computer. Only the study coordinator and the principal investigator will know the password to this file.

## 10.0 STATISTICAL CONSIDERATIONS

### 10.1.Study Design and Primary Endpoints

This is a single-arm prospective interventional study investigating the 4-year biochemical recurrence-free survival (BCRFS) following adjuvant or salvage SBRT. Other primary endpoints include physician-scored toxicity and patient-reported toxicity. The current study hypothesizes that utilizing SBRT to deliver 34 Gy in five fractions in the postoperative setting will allow an increase in 4-year BCRFS to 70%, compared with a historical rate of 50%.

### 10.2. Sample Size and Power Considerations

Historical data show the BCRFS time distribution is approximated reasonable well by the Weibull distribution with a shape parameter of 0.50 with a 4-year BCRFS rate of 56% in a large cohort of patients treated with a median postoperative RT dose of 66 Gy[17]. The study SBRT regimen of 34 Gy in five fractions of 6.8 Gy constitutes an equivalent dose in 2 Gy fractions of ~74 Gy, with the conservative α/β ratio estimation of 2 Gy. On the basis of the literature-derived sigmoid dose-response relationship of a 2% increase in 5-year BCRFS per Gy[2], we hypothesize that the 4-year BCRFS in patients treated with this regimen would be ~72%. This corresponds to a hazard ratio of log(0.56)/log(0.72)=1.77. With an overall sample size of 60 patients, we will achieve 84.7% power to detect a hazard ratio of 1.77 when comparing the treatment to the historical control, with a one-sided, one-sample logrank test[91], at a 0.05 significance level. We will round this up to a total of 60 patients for the purposes of trial enrollment. Patients will be accrued for a period of 4 years and follow-up continues for 4 years after the last subject is added. Given the same estimated HR of 1.77, the expanded sample size of 100 patients will provide a power of 97.0% with a one-sided, one-sample logrank test at a 0.05 significance level. If we stipulated that the historical 4-year BCRFS might have increased to 60% (versus 56%), the estimated HR would be 1.56, and we would have 83.9% power to detect this hazard ratio with a one-sided, one-sample logrank test at a 0.05 significance level provided a sample size of n=100 patients.

The study will also quantify the rate of early and late genitourinary (GU) or gastrointestinal toxicity and compare with historical control rates for post-operative radiotherapy using conventional fractionation. The reference toxicity rate will be an acute grade ≥2 GU toxicity rate (Common Terminology Criteria for Adverse Events version 4.03) of 20%, which is based off the toxicity rate in the 70 Gy arm of the recently published SAKK 09/10 trial3. With the assumed true toxicity rate of 20% and an overall sample size of 60 patients, a two-sided 95% confidence interval has a margin of error of +/- 11%, which is narrow enough to provide sufficient precision for the estimate of toxicity rate. With a sample size of 100, the two-sided 95% confidence interval has a margin of error of +/- 8.5%.

### 10.4. Analysis Plan

*10.4.1. Analysis of Primary Endpoint(s)*:

The primary efficacy endpoint is 4-year biochemical recurrence-free survival (BCRFS). Biochemical recurrence (BCR) is defined as serum PSA rising from the post-treatment nadir to a level of 0.4 ng/mL or more with a confirmatory second test, initiation of salvage androgen deprivation therapy, or continued rise in PSA after SBRT. The Kaplan-Meier product-limit estimate of the BCRFS will be estimated and presented graphically. One sample log-rank test will be used to test difference in BCRFS between intervention and historical control. The median BCRFS time will be calculated with 95% confidence interval. Summaries of the number and percentage of patients experiencing a biochemical recurrence will be provided. This endpoint will be evaluated at the following time points with respect to SBRT: 3 months (acute), two years, and four years.

Physician-scored toxicity will be represented by the rates of acute (early, within 90 days of SBRT) and late (90 or more days after SBRT)) genitourinary and gastrointestinal toxicity based on the Common Terminology Criteria for Adverse Events version 4.03 (CTCAE v 4.03) criteria. AEs and SAEs will be listed individually by patient.

Patient-reported toxicity outcomes will be evaluated by changes in the urinary incontinence, urinary obstruction, bowel, sexual function, and hormone/vitality domains on the Expanded Prostate Cancer Index-26 (EPIC-26) quality of life instrument. International Prostate Symptom Scores (IPSS) will also be obtained as measures of patient-reported toxicity. Patient-reported toxicity outcomes will be tabulated and reported. This endpoint will be evaluated at the following time points with respect to SBRT: 3 months (acute), two years, and four years.

*10.4.2. Analysis of Secondary Endpoint(s)*:

Point estimate and the corresponding 95% confidence interval will be calculated for the proportion of SBRT fractions for which on-line adaptive radiotherapy is required due to changes in organ-at-risk anatomy, in the subset of patients treated with MRI-guided radiotherapy.

*10.4.3. Analysis of Exploratory Endpoint(s)*:

Comparative analysis will be performed to assess toxicity profiles (both physician-scored and patient-reported) between patients treated utilizing a linear accelerator versus the MRI-guided device. Chi-square test or Fisher's exact test will be used for qualitative toxicity measurements, while unpaired t-test or Wilcoxon rank sum test will be used for quantitative toxicity metrics.

**10.5. Interim Analysis and Reports:**

Interim analysis: if the rate of grade 3 or higher adverse gastrointestinal or genitourinary events is higher than 20%, accrual will be halted and the study subjected to careful review. If the rate is higher than 30%, the study will be terminated.

We will follow up all patients closely and do not expect any patients to drop out of the trial except for death from another cause, so censoring due to other reasons would be unlikely. We expect to complete the accrual in 4 years. Our major primary endpoint is the 4-year biochemical recurrence-free survival. However, physician-scored toxicity is also a major endpoint of interest. In the interest of patient safety, we will conduct an interim safety analysis after 2 years, or after 30 patients have been enrolled—whichever comes first—wherein we will evaluate the actuarial rate of biochemical recurrence-free survival and examine the rate of grade 3 or higher adverse gastrointestinal or genitourinary events.

Interim reports will be prepared every six months until the results of the study are published. In general, the interim reports will contain information about patient accrual rate with projected completion dates, status of QA review and compliance rate of treatment per protocol, and the frequencies and severity of toxicity.

## 11.0 COST CONSIDERATIONS

The proposed treatment approach does not incur additional work-up, procedures, or costs.

## 12. REFERENCES

1. Stephenson AJ, Shariat SF, Zelefsky MJ, et al. Salvage radiotherapy for recurrent prostate cancer after radical prostatectomy. Jama 2004;291:1325-32.

2. King CR. The dose-response of salvage radiotherapy following radical prostatectomy: A systematic review and meta-analysis. Radiotherapy and oncology : journal of the European Society for Therapeutic Radiology and Oncology 2016;121:199-203.

3. Ghadjar P, Hayoz S, Bernhard J, et al. Acute Toxicity and Quality of Life After Dose-Intensified Salvage Radiation Therapy for Biochemically Recurrent Prostate Cancer After Prostatectomy: First Results of the Randomized Trial SAKK 09/10. Journal of clinical oncology : official journal of the American Society of Clinical Oncology 2015;33:4158-66.

4. Valicenti RK, Thompson I, Jr., Albertsen P, et al. Adjuvant and salvage radiation therapy after prostatectomy: American Society for Radiation Oncology/American Urological Association guidelines. International journal of radiation oncology, biology, physics 2013;86:822-8.

5. NCCN Clinical Practice Guidelines in Oncology: Prostate Cancer. 2015. (Accessed 11/30/2015, at http://www.nccn.org/professionals/physician_gls/pdf/prostate.pdf.)

6. Bolla M, van Poppel H, Tombal B, et al. Postoperative radiotherapy after radical prostatectomy for high-risk prostate cancer: long-term results of a randomised controlled trial (EORTC trial 22911). Lancet (London, England) 2012;380:2018-27.

7. Thompson IM, Tangen CM, Paradelo J, et al. Adjuvant radiotherapy for pathological T3N0M0 prostate cancer significantly reduces risk of metastases and improves survival: long-term followup of a randomized clinical trial. The Journal of urology 2009;181:956-62.

8. Wiegel T, Bartkowiak D, Bottke D, et al. Adjuvant radiotherapy versus wait-and-see after radical prostatectomy: 10-year follow-up of the ARO 96-02/AUO AP 09/95 trial. European urology 2014;66:243-50.

9. King CR. Adjuvant versus salvage radiotherapy for high-risk prostate cancer patients. Semin Radiat Oncol 2013;23:215-21.

10. Briganti A, Wiegel T, Joniau S, et al. Early salvage radiation therapy does not compromise cancer control in patients with pT3N0 prostate cancer after radical prostatectomy: results of a match-controlled multi-institutional analysis. European urology 2012;62:472-87.

11. Falchook AD, Chen RC. Adjuvant vs.salvage radiotherapy for patients at high risk for recurrence after radical prostatectomy. Urol Oncol 2015;33:451-5.

12. Bartkowiak D, Bottke D, Wiegel T. Adjuvant radiotherapy or early salvage radiotherapy in pT3R0 or pT3R1 prostate cancer. Curr Opin Urol 2013;23:360-5.

13. Hsu CC, Paciorek AT, Cooperberg MR, Roach M, 3rd, Hsu IC, Carroll PR. Postoperative radiation therapy for patients at high-risk of recurrence after radical prostatectomy: does timing matter? BJU international 2015;116:713-20.

14. Sineshaw HM, Gray PJ, Efstathiou JA, Jemal A. Declining Use of Radiotherapy for Adverse Features After Radical Prostatectomy: Results From the National Cancer Data Base. European urology 2015;68:768-74.

15. Kishan AU, Duchesne G, Wang PC, et al. Discord Among Radiation Oncologists and Urologists in the Postoperative Management of High-Risk Prostate Cancer. Am J Clin Oncol 2017.

16. Tesson S, Sundaresan P, Ager B, et al. Knowledge, attitudes and decision-making preferences of men considering participation in the TROG RAVES Prostate Cancer Trial (TROG 08.03). Radiotherapy and oncology : journal of the European Society for Therapeutic Radiology and Oncology 2016.

17. Tendulkar RD, Agrawal S, Gao T, et al. Contemporary Update of a Multi-Institutional Predictive Nomogram for Salvage Radiotherapy After Radical Prostatectomy. Journal of clinical oncology : official journal of the American Society of Clinical Oncology 2016.

18. Stish BJ, Pisansky TM, Harmsen WS, et al. Improved Metastasis-Free and Survival Outcomes With Early Salvage Radiotherapy in Men With Detectable Prostate-Specific Antigen After Prostatectomy for Prostate Cancer. Journal of clinical oncology : official journal of the American Society of Clinical Oncology 2016.
19. Spratt DE, Zhang J, Santiago-Jimenez M, et al. Development and Validation of a Novel Integrated Clinical-Genomic Risk Group Classification for Localized Prostate Cancer.
20. Spratt DE, Yousefi K, Deheshi S, et al. Individual Patient-Level Meta-Analysis of the Performance of the Decipher Genomic Classifier in High-Risk Men After Prostatectomy to Predict Development of Metastatic Disease.
21. Den RB, Yousefi K, Trabulsi EJ, et al. Genomic classifier identifies men with adverse pathology after radical prostatectomy who benefit from adjuvant radiation therapy.
22. Kuban DA, Tucker SL, Dong L, et al. Long-term results of the M. D. Anderson randomized dose-escalation trial for prostate cancer. International journal of radiation oncology, biology, physics 2008;70:67-74.
23. Zietman AL, Bae K, Slater JD, et al. Randomized trial comparing conventional-dose with high-dose conformal radiation therapy in early-stage adenocarcinoma of the prostate: long-term results from proton radiation oncology group/american college of radiology 95-09. Journal of clinical oncology : official journal of the American Society of Clinical Oncology 2010;28:1106-11.
24. Heemsbergen WD, Al-Mamgani A, Slot A, Dielwart MF, Lebesque JV. Long-term results of the Dutch randomized prostate cancer trial: impact of dose-escalation on local, biochemical, clinical failure, and survival. Radiotherapy and oncology : journal of the European Society for Therapeutic Radiology and Oncology 2014;110:104-9.
25. Dearnaley DP, Jovic G, Syndikus I, et al. Escalated-dose versus control-dose conformal radiotherapy for prostate cancer: long-term results from the MRC RT01 randomised controlled trial. Lancet Oncol 2014;15:464-73.
26. Kalbasi A, Li J, Berman A, et al. Dose-Escalated Irradiation and Overall Survival in Men With Nonmetastatic Prostate Cancer. JAMA oncology 2015;1:897-906.
27. Swanson GP, Hussey MA, Tangen CM, et al. Predominant treatment failure in postprostatectomy patients is local: analysis of patterns of treatment failure in SWOG 8794. Journal of clinical oncology : official journal of the American Society of Clinical Oncology 2007;25:2225-9.
28. De Meerleer G, Fonteyne V, Meersschout S, et al. Salvage intensity-modulated radiotherapy for rising PSA after radical prostatectomy. Radiotherapy and oncology : journal of the European Society for Therapeutic Radiology and Oncology 2008;89:205-13.
29. Hall EJG, A.J. Radiobiology for the Radiologist. 7th ed. Philadelphia: Lippincott Williams & Wilkins; 2011.
30. Brenner DJ, Hall EJ. Fractionation and protraction for radiotherapy of prostate carcinoma. International journal of radiation oncology, biology, physics 1999;43:1095-101.
31. Fowler JF, Toma-Dasu I, Dasu A. Is the alpha/beta ratio for prostate tumours really low and does it vary with the level of risk at diagnosis? Anticancer research 2013;33:1009-11.
32. Dasu A, Toma-Dasu I. Prostate alpha/beta revisited -- an analysis of clinical results from 14 168 patients. Acta oncologica (Stockholm, Sweden) 2012;51:963-74.
33. Miralbell R, Roberts SA, Zubizarreta E, Hendry JH. Dose-fractionation sensitivity of prostate cancer deduced from radiotherapy outcomes of 5,969 patients in seven international institutional datasets: alpha/beta = 1.4 (0.9-2.2) Gy. International journal of radiation oncology, biology, physics 2012;82:e17-24.
34. Dearnaley D, Syndikus I, Mossop H, et al. Conventional versus hypofractionated high-dose intensity-modulated radiotherapy for prostate cancer: 5-year outcomes of the randomised, non-inferiority, phase 3 CHHiP trial. The Lancet Oncology 2016;17:1047-60.
35. Lee WR, Dignam JJ, Amin MB, et al. Randomized Phase III Noninferiority Study Comparing Two Radiotherapy Fractionation Schedules in Patients With Low-Risk Prostate Cancer. Journal of clinical oncology : official journal of the American Society of Clinical Oncology 2016;34:2325-32.

36. Pollack A, Walker G, Horwitz EM, et al. Randomized trial of hypofractionated external-beam radiotherapy for prostate cancer. Journal of clinical oncology : official journal of the American Society of Clinical Oncology 2013;31:3860-8.
37. Incrocci L, Wortel RC, Alemayehu WG, et al. Hypofractionated versus conventionally fractionated radiotherapy for patients with localised prostate cancer (HYPRO): final efficacy results from a randomised, multicentre, open-label, phase 3 trial. The Lancet Oncology 2016;17:1061-9.
38. Arcangeli G, Saracino B, Gomellini S, et al. A prospective phase III randomized trial of hypofractionation versus conventional fractionation in patients with high-risk prostate cancer. International journal of radiation oncology, biology, physics 2010;78:11-8.
39. Catton CN, Lukka H, Gu CS, et al. Randomized Trial of a Hypofractionated Radiation Regimen for the Treatment of Localized Prostate Cancer. Journal of clinical oncology : official journal of the American Society of Clinical Oncology 2017:Jco2016717397.
40. Kruser TJ, Jarrard DF, Graf AK, et al. Early hypofractionated salvage radiotherapy for postprostatectomy biochemical recurrence. Cancer 2011;117:2629-36.
41. Wong GW, Palazzi-Churas KL, Jarrard DF, et al. Salvage hypofractionated radiotherapy for biochemically recurrent prostate cancer after radical prostatectomy. International journal of radiation oncology, biology, physics 2008;70:449-55.
42. Massaccesi M, Cilla S, Deodato F, et al. Hypofractionated intensity-modulated radiotherapy with simultaneous integrated boost after radical prostatectomy: preliminary results of a phase II trial. Anticancer research 2013;33:2785-9.
43. Cozzarini C, Fiorino C, Deantoni C, et al. Higher-than-expected severe (Grade 3-4) late urinary toxicity after postprostatectomy hypofractionated radiotherapy: a single-institution analysis of 1176 patients. European urology 2014;66:1024-30.
44. Lewis SL, Patel P, Song H, et al. Image Guided Hypofractionated Postprostatectomy Intensity Modulated Radiation Therapy for Prostate Cancer. International journal of radiation oncology, biology, physics 2016;94:605-11.
45. Syndikus I, Pickles T, Kostashuk E, Sullivan LD. Postoperative radiotherapy for stage pT3 carcinoma of the prostate: improved local control. The Journal of urology 1996;155:1983-6.
46. Lee LW, McBain CA, Swindell R, Wylie JP, Cowan RA, Logue JP. Hypofractionated radiotherapy as salvage for rising prostate-specific antigen after radical prostatectomy. Clinical oncology (Royal College of Radiologists (Great Britain)) 2004;16:517-22.
47. Katayama S, Striecker T, Kessel K, et al. Hypofractionated IMRT of the prostate bed after radical prostatectomy: acute toxicity in the PRIAMOS-1 trial. International journal of radiation oncology, biology, physics 2014;90:926-33.
48. Fersino S, Tebano U, Mazzola R, et al. Moderate Hypofractionated Postprostatectomy Volumetric Modulated Arc Therapy With Daily Image Guidance (VMAT-IGRT): A Mono-institutional Report on Feasibility and Acute Toxicity. Clinical genitourinary cancer 2017.
49. Kupelian P, Mehta NH, King C, Steinberg M, Finkelstein SE, Fernandez E. Stereotactic body radiation therapy for prostate cancer: Rational and reasonable. Practical radiation oncology 2015;5:188-92.
50. Kishan AUK, C.R. Stereotactic body radiotherapy for low- and intermediate-risk prostate cancer. Seminars in Radiation Oncology 2017;In press.
51. King CR, Freeman D, Kaplan I, et al. Stereotactic body radiotherapy for localized prostate cancer: pooled analysis from a multi-institutional consortium of prospective phase II trials. Radiotherapy and oncology : journal of the European Society for Therapeutic Radiology and Oncology 2013;109:217-21.

52. King CR, Collins S, Fuller D, et al. Health-related quality of life after stereotactic body radiation therapy for localized prostate cancer: results from a multi-institutional consortium of prospective trials. International journal of radiation oncology, biology, physics 2013;87:939-45.
53. ASTRO Model Policy. 2013. (Accessed August 1, 2016, at https://www.astro.org/uploadedFiles/Main_Site/Practice_Management/Reimbursement/2013HPcoding%20guidelines_SBRT_Final.pdf.)
54. Siegel R, Naishadham D, Jemal A. Cancer statistics, 2012. CA: a cancer journal for clinicians 2012;62:10-29.
55. Mariotto AB, Yabroff KR, Shao Y, Feuer EJ, Brown ML. Projections of the cost of cancer care in the United States: 2010-2020. Journal of the National Cancer Institute 2011;103:117-28.
56. Laviana AA, Ilg AM, Veruttipong D, et al. Utilizing time-driven activity-based costing to understand the short- and long-term costs of treating localized, low-risk prostate cancer. Cancer 2016;122:447-55.
57. Hodges JC, Lotan Y, Boike TP, Benton R, Barrier A, Timmerman RD. Cost-effectiveness analysis of stereotactic body radiation therapy versus intensity-modulated radiation therapy: an emerging initial radiation treatment option for organ-confined prostate cancer. Journal of oncology practice / American Society of Clinical Oncology 2012;8:e31s-7s.
58. Yu JB, Cramer LD, Herrin J, Soulos PR, Potosky AL, Gross CP. Stereotactic body radiation therapy versus intensity-modulated radiation therapy for prostate cancer: comparison of toxicity. Journal of clinical oncology : official journal of the American Society of Clinical Oncology 2014;32:1195-201.
59. Kishan AUF, D.B.; Steinberg, M.L.; Ramirez, V.; Agazaryan, N.; Ruan, D.; Cao, M.; Kupelian, P.; King, C.R. Stereotactic body radiotherapy for high-risk prostate cancer (5 treatment days): Toxicity results of a phase II trial. Journal of Clinical Oncology 2017;35.
60. Sampath SY, B.; Frankel, P.; Liu, A.; Tsai, P.; Twardowski, P.; Stein, C.; Kawachi, M.; Dandapani, S.V.; Schultheiss, T.E.; Wong, J.Y.C. Prostate Bed Stereotactic Body Radiation Therapy (PB-SBRT) for Postprostatectomy Biochemical Recurrence: First Toxicity Results of a Phase 1 Dose-Escalation Trial. American Society for Radiation Oncology; 2016; Boston, MA. p. E227–E8.
61. Emami B, Lyman J, Brown A, et al. Tolerance of normal tissue to therapeutic irradiation. International journal of radiation oncology, biology, physics 1991;21:109-22.
62. Marks LB, Yorke ED, Jackson A, et al. Use of normal tissue complication probability models in the clinic. International journal of radiation oncology, biology, physics 2010;76:S10-9.
63. Denham JW, Steigler A, Lamb DS, et al. Short-term neoadjuvant androgen deprivation and radiotherapy for locally advanced prostate cancer: 10-year data from the TROG 96.01 randomised trial. Lancet Oncol 2011;12:451-9.
64. D'Amico AV, Chen MH, Renshaw AA, Loffredo M, Kantoff PW. Androgen suppression and radiation vs radiation alone for prostate cancer: a randomized trial. JAMA 2008;299:289-95.
65. Jones CU, Hunt D, McGowan DG, et al. Radiotherapy and short-term androgen deprivation for localized prostate cancer. N Engl J Med 2011;365:107-18.
66. Bolla M, Van Tienhoven G, Warde P, et al. External irradiation with or without long-term androgen suppression for prostate cancer with high metastatic risk: 10-year results of an EORTC randomised study. Lancet Oncol 2010;11:1066-73.
67. Pilepich MV, Winter K, Lawton CA, et al. Androgen suppression adjuvant to definitive radiotherapy in prostate carcinoma--long-term results of phase III RTOG 85-31. International journal of radiation oncology, biology, physics 2005;61:1285-90.
68. King CR, Presti JC, Jr., Gill H, Brooks J, Hancock SL. Radiotherapy after radical prostatectomy: does transient androgen suppression improve outcomes? International journal of radiation oncology, biology, physics 2004;59:341-7.

69. Ost P, Cozzarini C, De Meerleer G, et al. High-dose adjuvant radiotherapy after radical prostatectomy with or without androgen deprivation therapy. International journal of radiation oncology, biology, physics 2012;83:960-5.
70. Carrie C, Hasbini A, de Laroche G, et al. Salvage radiotherapy with or without short-term hormone therapy for rising prostate-specific antigen concentration after radical prostatectomy (GETUG-AFU 16): a randomised, multicentre, open-label phase 3 trial. Lancet Oncol 2016;17:747-56.
71. Shipley WU, Seiferheld W, Lukka HR, et al. Radiation with or without Antiandrogen Therapy in Recurrent Prostate Cancer. N Engl J Med 2017;376:417-28.
72. Cozzarini C. Whole-pelvis Radiotherapy in the Radiation Treatment of Intermediate- and High-risk Prostate Cancer: How to Improve the Therapeutic Ratio of a Potentially Effective but still Unsatisfactory Treatment? European urology 2017;71:44-5.
73. Pommier P, Chabaud S, Lagrange JL, et al. Is There a Role for Pelvic Irradiation in Localized Prostate Adenocarcinoma? Update of the Long-Term Survival Results of the GETUG-01 Randomized Study. International journal of radiation oncology, biology, physics 2016;96:759-69.
74. Lawton CA, DeSilvio M, Roach M, 3rd, et al. An update of the phase III trial comparing whole pelvic to prostate only radiotherapy and neoadjuvant to adjuvant total androgen suppression: updated analysis of RTOG 94-13, with emphasis on unexpected hormone/radiation interactions. International journal of radiation oncology, biology, physics 2007;69:646-55.
75. Spiotto MT, Hancock SL, King CR. Radiotherapy after prostatectomy: improved biochemical relapse-free survival with whole pelvic compared with prostate bed only for high-risk patients. International journal of radiation oncology, biology, physics 2007;69:54-61.
76. Moghanaki D, Koontz BF, Karlin JD, et al. Elective irradiation of pelvic lymph nodes during postprostatectomy salvage radiotherapy. Cancer 2013;119:52-60.
77. Song C, Kang HC, Kim JS, et al. Elective pelvic versus prostate bed-only salvage radiotherapy following radical prostatectomy: A propensity score-matched analysis. Strahlentherapie und Onkologie : Organ der Deutschen Rontgengesellschaft [et al] 2015;191:801-9.
78. Woo S, Kaplan I, Roach M, Bagshaw M. Formula to estimate risk of pelvic lymph node metastasis from the total Gleason score for prostate cancer. The Journal of urology 1988;140:387.
79. Kim BS, Lashkari A, Vongtama R, Lee SP, Parker RG. Effect of pelvic lymph node irradiation in salvage therapy for patients with prostate cancer with a biochemical relapse following radical prostatectomy. Clinical prostate cancer 2004;3:93-7.
80. Kishan AU, Lamb JM, Jani SS, Kang JJ, Steinberg ML, King CR. Pelvic nodal dosing with registration to the prostate: implications for high-risk prostate cancer patients receiving stereotactic body radiation therapy. International journal of radiation oncology, biology, physics 2015;91:832-9.
81. Michalski JM, Lawton C, El Naqa I, et al. Development of RTOG consensus guidelines for the definition of the clinical target volume for postoperative conformal radiation therapy for prostate cancer. International journal of radiation oncology, biology, physics 2010;76:361-8.
82. Lawton CA, Michalski J, El-Naqa I, et al. RTOG GU Radiation oncology specialists reach consensus on pelvic lymph node volumes for high-risk prostate cancer. International journal of radiation oncology, biology, physics 2009;74:383-7.
83. Dalela D, Loppenberg B, Sood A, Sammon J, Abdollah F. Contemporary Role of the Decipher(R) Test in Prostate Cancer Management: Current Practice and Future Perspectives. Reviews in urology 2016;18:1-9.
84. Erho N, Crisan A, Vergara IA, et al. Discovery and validation of a prostate cancer genomic classifier that predicts early metastasis following radical prostatectomy. PloS one 2013;8:e66855.

85. Den RB, Feng FY, Showalter TN, et al. Genomic prostate cancer classifier predicts biochemical failure and metastases in patients after postoperative radiation therapy. International journal of radiation oncology, biology, physics 2014;89:1038-46.
86. Den RB, Yousefi K, Trabulsi EJ, et al. Genomic classifier identifies men with adverse pathology after radical prostatectomy who benefit from adjuvant radiation therapy. J Clin Oncol 2015;33:944-51.
87. Ross AE, Feng FY, Ghadessi M, et al. A genomic classifier predicting metastatic disease progression in men with biochemical recurrence after prostatectomy. Prostate cancer and prostatic diseases 2014;17:64-9.
88. Karnes RJ, Bergstralh EJ, Davicioni E, et al. Validation of a genomic classifier that predicts metastasis following radical prostatectomy in an at risk patient population. The Journal of urology 2013;190:2047-53.
89. Cooperberg MR, Davicioni E, Crisan A, Jenkins RB, Ghadessi M, Karnes RJ. Combined value of validated clinical and genomic risk stratification tools for predicting prostate cancer mortality in a high-risk prostatectomy cohort. Eur Urol 2015;67:326-33.
90. Freedland SJ, Choeurng V, Howard L, et al. Utilization of a Genomic Classifier for Prediction of Metastasis Following Salvage Radiation Therapy after Radical Prostatectomy. Eur Urol 2016;70:588-96.
91. Wu J. Sample size calculation for the one-sample log-rank test. Pharmaceutical statistics 2015;14:26-33.

## APPENDICES

## STUDY CALENDAR

| Procedure | Pre Study | Pre-RT Enrollment (Up to 4 months prior) | Baseline Pre-SBRT Day 1 | On Tx SBRT 5 fx | At 2 weeks[a,b] (+/- 3 days) | POST TREATMENT VISITS (+/- 8 weeks) | | | | | | | | |
|---|---|---|---|---|---|---|---|---|---|---|---|---|---|---|
| | | | | | | *1 month[a,b] | 3 month[a,b] | 6 month[a,b] | 9 month[a,b] | 12 month[a,b] | 18 month[a,b] | 24 month[a,b] | Q 6month x 4 yrs[a,b] 30M 48M 36M 54M 42M 60M | EOS Or Early Term Visit[b] |
| Informed Consent | x | | | | | | | | | | | | | |
| Demographics | x | | | | | | | | | | | | | |
| Medical History | x | | | | | | | | | | | | | |
| *SOC Physical Exam [f] | x | | | | | | | | | | | | | x |
| Toxicity assessments / Quality of Life Questionnaires | | x [f] | | | x | x | x | x | x | x | x | x | x | x |
| SOC Non-Contrast Pelvic CT scan[c] | | x | | | | | | | | | | | | |
| SOC MRI of the Pelvis | | x | | | | | | | | | | | | |
| SOC Blood Samples (PSA , Testosterone) | | x | | | | | x[d] | x[d] | x[d] | x[d] | x[d] | x[d] | x[d] | x |
| Translational saliva collection | | x [e] | x [e] | | | | | | | | | | | |
| Translational blood collection | | | X [e] | x☼ | | | x[e] | x[e] | | | | | | |
| Record Radiation Therapy - 5 fractions | | | | x | | | | | | | | | | |

*Standard of care ☼ Translational blood collection Mid-draw

a.) Visits can be conducted over the telephone, with quality of life questionnaires not mandatory. When required, initial consent and re-consenting of these subjects may occur by phone using an IRB-approved re-consent script or other document.

b.) The above visits occur as part of the patient's conventional care for patients that have agreed to undergo SBRT treatment following their prostatectomy. Although the windows is recommended, certain factors may affect adherence and will not be considered a deviation.

c.) For patients with PSA <1.0 ng/mL, the treatment planning CT can substitute for a diagnostic CT scan (in this case, the CT simulation should be within 6 weeks of radiotherapy initiation)

d.) These SOC blood samples can be drawn remotely, in the event that the patient is following up outside the UCLA system. In these cases, the lab reports should be provided to the study investigators. During post treatment period for patients not receiving hormonal therapy, testosterone monitoring will not be necessary. For patients receiving hormonal therapy, per physician discretion testosterone should be checked for the first 12-18 months, depending on testosterone recovery, but will not be mandated.

e.) These samples will occur whenever feasible

f.) May occur as late as Day 1 prior to any study treatment for patients consented remotely
